# “The system is obviously bonkers”: The APC Trap and the bind of scholarly publishing across four research intensive institutions in the U.S.

**DOI:** 10.1371/journal.pone.0351430

**Published:** 2026-07-01

**Authors:** Melissa H. Cantrell, Rachel Caldwell, Jennifer A. Mezick, Matthew Estill, Lauren B. Collister

**Affiliations:** 1 University Libraries, University of Colorado Boulder, Boulder, Colorado‌‌, United States of America; 2 University Libraries, University of Massachusetts Amherst, Amherst, Massachusetts, United States of America; 3 University Libraries, University of Tennessee Knoxville, Knoxville, Tennessee, United States of America; 4 University Library System, University of Pittsburgh, Pittsburgh, Pennsylvania‌‌, United States of America; 5 Invest in Open Infrastructure, Portland, Oregon, United States of America; Cranfield University, UNITED KINGDOM OF GREAT BRITAIN AND NORTHERN IRELAND

## Abstract

This study analyzes the cognitive dissonance displayed by authors at research intensive institutions in the United States (U.S.) regarding the scholarly publishing of Open Access (OA) publications. A qualitative analysis of 154 open-ended responses was conducted. Eight codes were identified, then each response was analyzed for the codes. The codes themselves coalesce around a phenomenon the authors of this study term the article processing charge (APC) Trap, in which privileged and well-resourced researchers in the U.S. experience highly contradictory sentiments about OA publishing. The cognitive dissonance characteristic of the APC Trap result in strong feelings of conflict as well as powerlessness. The qualitative analysis reveals the APC Trap as a deeply layered phenomenon, with manifestation dependent on positionality, including demographic features and interactions between APC Trap codes. While much has been made of the failings of the APC model of OA publishing for under-resourced researchers, this study reveals the ways this publishing model is unsustainable even for privileged researchers most well-positioned to participate.

## Introduction

This paper examines the complex attitudes and experiences of researchers regarding article processing charges (APCs) in open access (OA) publishing. The APC model’s introduction is usually attributed to BioMed Central in the year 2000 as a means to support financial sustainability for publishers who offer OA to scholarly articles in lieu of subscription revenues [1]. The problematization of the APC-based business model began shortly after its introduction. Authors initially approached APCs as a binary choice of whether to pay or not: “once authors are willing or able to pay an APC, [...] they are willing to pay them with little regard to the size of an APC” [2]. Pricing for APCs often differs widely based on discipline of the journal or location of the journal [3]. Furthermore, publishers report pricing APCs based on journal prestige rather than production costs, and a study of data from the United Kingdom shows a correlation between increases in APCs for Open Access journals and higher journal citation impact [4]. Yet, APC hyperinflation was documented at 5–10% annual increases with little impact on growth at major publishers or evidence for need to increase prices [2,3].

Beyond the pricing models, literature has consistently highlighted how APCs in general create “barriers” excluding researchers from less well-resourced institutions and countries [1,2,5,6] and reinforcing existing inequities [3,7–9]. Editors surveyed for a study of library and information science journals reported viewing APCs as barriers to inclusivity, expressing concern that APCs replicate financial inequalities and disadvantage under-resourced authors, particularly from the Global South or smaller institutions [10]. While authors at institutions with OA budgets viewed OA as complementary to other factors like reputation and editorial practices, those without OA budgets saw APCs as major deterrents to publishing and making their research widely available. In a 2021 study of researchers from India, authors with institutional support viewed OA favorably, with one researcher explaining: “Sometimes I think about open access as my first choice...because I’m coming from a low-resource country. And I know how people there suffer in finding the information.” Conversely, unsupported authors reported: “I simply won’t consider those journals because I just can’t [pay] those fees...even if it’s probably the best journal for me” [6]. While researchers value OA in principle, cost remains a major deterrent when not covered by grants or institutions [7].

This study explores the navigation of the open access publishing landscape by authors who encountered APCs. In previous work, the authors conducted a survey to understand the resources that researchers used to pay for APCs [11]. In addition to the quantitative findings, the responses to open-ended questions in the survey demonstrated cognitive dissonance among researchers who show a strong willingness to publish OA while viewing APC prices as increasingly unreasonable and unfeasible. These findings aligned with observations of the “APC effect” introduced by Klebel & Ross-Hellauer [3]. The “APC effect” describes how better-resourced institutions publish in journals with higher APCs and higher perceived prestige. In fact, institutions in wealthier countries pay 42% higher average APCs, creating stratification where less-resourced institutions are blocked from costly, prestigious publications. These charges thus have “a chilling effect on opportunity and equality for researchers from less prestigious or less wealthy institutions” [3]. While publishers offer waivers for some low- and middle-income countries, many countries like India don’t qualify despite researchers facing substantial constraints. In one study, doctors and researchers reported having plenty of clinical material and the potential to write and publish, but APCs limited their options to submit academic papers [6]. Even in a study of over 180,000 scholars at research institutions in the United States, those who are more likely to author an open access article with an associated APC were those who had greater access to resources and job security, including those who were at an advanced career stage, had federal research funding, were apparently male in gender, and employed at a prestigious institution in a STEM discipline [12].

The existing literature has documented the APC effect specifically leading to the exclusion of under-resourced researchers. The qualitative findings in this paper extend that concept. While the APC effect represents a barrier for many researchers globally, a complementary “APC Trap” binds privileged researchers who feel compelled to participate in OA publishing while simultaneously feeling powerless to change its inequities. This “APC Trap” is investigated using survey results, which include direct comments from well-resourced researchers about their experiences with APCs. The researchers surveyed report simultaneous price insensitivity and price outrage toward OA models, which results in a bind for those who feel obligated to use APC models despite strong feelings about their unjust impacts. This complex thinking is characterized by the “bind” of the APC Trap—a contradictory situation where APC “barriers” combine with professional anxieties and equity concerns. This results in a feeling of inability to change the status quo, even among the world’s most privileged and well-resourced researchers. This article explores how the APC Trap manifests in qualitative findings, including its disciplinary and institutional variation. Ultimately, the APC Trap represents a difficult bind without easy solutions. The evidence presents an ethical compromise that demonstrates the APC model is not working for *any author* if it is not working for those who have the easiest path to pay and realize the greatest benefit.

## Materials and methods

### Study sites and IRB approval‌‌

This study was reviewed by the University of Colorado Review Board and accepted under the review category Exempt (IRB Protocol Number: 23–0216). The study is drawn from a dataset collected in 2023 about publications from four (U.S.) research intensive universities (Carnegie Classification of R1): the University of Colorado Boulder (UCB), the University of Massachusetts Amherst (UMass), the University of Pittsburgh (Pitt), and the University of Tennessee Knoxville (UTK). These institutions are all public universities and sit within the upper tier of U.S. institutions by annual research and development expenditure [13]. These institutions accordingly represent the well-resourced end of the global research landscape. The four sites are geographically situated in different areas of the U.S. and have similar academic profiles with degree and research representation across disciplines with the exception to hospital-based medical research. The University of Pittsburgh has a large medical school that is included in this study. The other institutions exclude medical school researchers from the data because those medical schools report through different campuses with different administrative and budgetary structures. The study protocol underwent a multi-site IRB approval, with the University of Colorado Boulder serving as the primary IRB review institution. Co-Investigators at the other institutions submitted protocols for supplemental IRB approval, and the one Co-Investigator not affiliated with an institution of higher education submitted an Individual Investigator Agreement. The study was deemed exempt from full board review by all four institutions. Data were collected via a Qualtrics questionnaire during the recruitment period between October 31, 2023 and November 21, 2023. Consent was obtained by having respondents read and accept an informed consent statement prior to completing the survey.

### Survey design and distribution

After the data were collected, the dataset was organized by institution and grouped into OA types: Gold OA and Hybrid OA. These types were based on reporting categories in Dimensions, a proprietary, comprehensive scientific research database [14]. Each of these types was then divided into three broad academic areas: Humanities and Social Sciences (HSS), Health and Medicine (HM), and Natural Sciences and Engineering (NSE). These three disciplinary categories are based on the Fields of Research Codes included in the Dimensions dataset [14].

Six different surveys (two within each HSS, HM, NSE) were created using the Qualtrics platform, and these surveys were customized based on OA type (Gold OA or Hybrid OA). For further details about the survey, data, and methodology for quantitative analysis, see the previous paper detailing quantitative findings [11]. All surveys included the same two free-response questions analyzed for this paper. Survey-based analysis with open-ended questioning is well-suited for researching scholarly publishing practices and perceptions as an effective method to capture nuanced attitudes and decision-making processes [10]. Manual coding of free text responses allows for theme development that incorporates complex social and institutional factors which can influence publishing behaviors [7,10]. These methods align with the field’s focus on understanding motivations, barriers, and values, and provide contextual insights to inform future scholarly communication policy and practice. This study used a purposive sampling method, meaning that participants were selected based on specific criteria rather than chosen at random. This allowed the study to focus on individuals who were most relevant to the research questions – faculty authors engaged in OA publishing who were likely to have encountered an article processing charge. This approach helped ensure that the data would be useful and applicable to the goals of the research, which was to identify the ways authors at R1 institutions pay for APCs and to understand what fees they viewed as reasonable. This purposive sampling allowed for a targeted analysis of the sentiments and funding sources of well-resourced researchers within the U.S. As the biggest users and drivers of the APC model of open access publishing, the sentiments of this subset of researchers may serve as a bellwether for the overall sustainability of the model globally.

While the survey collected both quantitative and qualitative data related to faculty publishing patterns, this paper focuses on the qualitative responses to two open-ended questions included at the end of each survey. All respondents received the same open-ended questions, regardless of whether the survey was for a Hybrid OA or Gold OA journal. These questions were designed to provide space for respondents to offer additional context, express concerns, or raise issues that may not have been fully addressed in the preceding survey questions.

The first open-ended question asked, “Do you have any additional comments regarding authors’ ability to pay publishing fees for Open Access?” This question aimed to elicit personal perspectives, institutional challenges, or concerns related to APCs and the economics of OA publishing. The second question invited broader reflection: “Do you have any additional comments about the topics in this survey?” This prompt allowed participants to provide feedback on the survey content, share general insights about OA publishing, or raise related issues not explicitly covered by the survey.

### Coding procedure

Open-ended responses were consolidated into a single spreadsheet and the dataset was prepared for analysis with each respondent assigned a unique identifier and a short institution code.

Analysis followed an inductive thematic approach, in which codes were developed from the data itself rather than applied from a pre-existing framework. The response data was initially reviewed to identify recurring patterns, concepts, and sentiments that reflect respondents’ experiences, perceptions, or attitudes. Frequently mentioned themes were normalized into codes. Responses representative of specific themes are included as examples in the Code Book to guide the subsequent coding of survey responses. The codes are discussed fully in the Results section.

Following the development of eight codes, each researcher independently reviewed participant responses. For each code, a “yes” or “no” was assigned, indicating its applicability to a given response. Codes were applied to the full set of responses from a single respondent, rather than individual statements or phrases. Therefore, each response could be assigned multiple codes which reflected themes demonstrated across the entirety of the participant’s comments.

An iterative refinement process was used to ensure coding consistency and accuracy. After an initial coding review, the Code Book was refined to address any ambiguities. To measure agreement, an Excel-based formula was developed to calculate consensus on “yes” and “no” assignments. A code was definitively applied to a respondent if four or more researchers reached agreement. If there were less than four in agreement, all researchers discussed each specific case to reach a consensus, leading to further refinements of the Code Book. This iterative process of independent coding, discussion of discrepancies, and Code Book refinement continued until consensus was reached on codes applied to each participant response.

## Results

Of the 322 respondents who consented and completed the substantive portion of the survey, 154 (47.8%) provided substantive responses to at least one of the two open-ended questions. Responses such as “No comment,” “N/A,” or similar single-word dismissals were excluded as non-substantive. The remaining 168 respondents completed the quantitative portion but either left both open-ended questions blank (n = 157) or provided non-substantive text (n = 11).

The two open-ended questions drew different volumes of substantive responses. The first (“Do you have any additional comments regarding authors’ ability to pay publishing fees for open access?”) elicited substantive text from 135 respondents (87.7% of substantive responses), while the second, more general question drew substantive text from 55 respondents (35.7%). Thirty-six respondents provided substantive text to both questions. Because respondents did not appear to distinguish between the two prompts in the content of their answers, the two responses were aggregated per respondent for coding, yielding a single qualitative response per substantive respondent (n = 154).

Substantive response rates varied modestly across the four institutions, ranging from 42.5% at UTK to 54.8% at UCB, and across disciplines, ranging from 42.0% in NSE to 56.0% in HSS. Response rates differed more sharply by OA type: 50.6% of authors published in Gold OA journals provided substantive responses, compared to 34.5% of authors published in Hybrid journals. The smaller Hybrid pool (n = 55 completed surveys) warrants caution in interpretation, but the disparity is consistent with the broader pattern described in the Discussion, in which Hybrid publishing is increasingly mediated by transformative agreements that obscure APCs from individual authors.

The thematic analysis of the 154 qualitative responses identified several core motifs around which authors were fixated in relation to APCs, and which worked together to reveal a contradictory but complementary sense of obligation and exasperation regarding payment for OA publishing. This bind represents an acute cognitive dissonance – coined here as the APC Trap.

Ultimately, eight major APC Trap codes were identified. Many more themes were initially identified; however, some were not indicative of the APC Trap, and others were combined or consolidated into a single code. For example, concerns about the future of graduate students and their inability to pay, public and taxpayer concerns, and concerns for researchers with less or no grant funding are all included in the code for *Diversity or Equity*. In the original analysis, there was a ninth code, called *Presupposition of Cost*, which is described following the Code Book, but which was not included in the final analysis because of concerns that the open-ended questions may have been leading respondents towards answers that include a presupposition of costs associated with OA publishing.

Below, the eight APC Trap codes are described, along with illustrative quotes. Quotes included in the following section were selected based on how clearly they showcase each code. This Code Book may be of use for future research and development of this model.

### Code book

#### Code: Financial concern.

This code focused on specific and personal financial hardships or worries on the part of the respondent regarding payment of the APC.

*More journals are forcing open access fees if you publish with them. I publish often so it does stress me out where I’ll always come up with this money from.* (#249, UTK)

*I have ample grant and internal support; payment for me is feasible, albeit it is expensive and a drain on resources. I have colleagues with far less resources which precludes their ability to pay for open-access publishing.* (#32, Pitt)

#### Code: Diversity or equity.

This code was applied to responses indicating APCs are hurting the ability to meet diversity and/or equity goals. Equity may refer to any number of imbalances between different parties, including references to “pay-to-play” scenarios and acknowledgement that some have deeper pockets than others. Common themes in this code included the perception that those who can’t pay for open access would suffer from lower exposure and reader access to their research, as well as increased bias towards the subjects of interest for wealthier institutions.

*I just think it is inherently unfair that the public doesn’t have open access to ALL published materials, especially when the opportunity to pay for publishing is not equally distributed among faculty. We need a better system! As you can see by my answers above, I object to the fees on principle, not because of the cost. Why should I benefit from paying a fee that others’ can’t afford? Why would I want this to be a reason why my work is used/cited by others? It should be read and used/cited on its merits, not some artifact of gamesmanship or privilege.* (#25, Pitt)

*It’s a diversity and equity issue. People from historically marginalized groups are least likely to be able to pay publication fees. Plus, publishing companies make LOTS OF MONEY and do not need these fees to be this high.* (#158, UCB)

#### Code: Threshold of reasonableness.

This code was used when respondents indicated that there was an upper limit of their tolerance for APCs. This theme is broader than *Financial Concern* (which focuses on specific, personal concerns for affording fee) and can include more general statements about the state of the field and questioning the reasonableness of the current system of APCs in OA publishing. This code also applies to responses that call out the exploitative or excessive practices of publishers, implying that there are “reasonable” limits for fees and profit.

*I recognize that there is labor involved in the publishing industry, but the costs to publish are out of control. Especially when you consider the profit margins enjoyed by these publishers.* (#272, UMass)

*I want to emphasize that help from the University and the departments is greatly appreciated. The cost of publishing has gone up significantly in the last 5 years that it is very difficult to find that kind of money. The amount needed to publish is very unreasonable and I do not understand why the cost has increased. It is very frustrating to investigators like myself, who want to publish in high impact journals, but, cannot always do so.* (#227, UTK)

#### Code: Restriction of decisions.

This code indicates that respondents made choices they might not otherwise have made because of an APC – specifically, that they had to make a choice for themselves, not that unnamed other parties have their decisions restricted. Restrictions included mandates or grant requirements that restrict a possible set of publication outlets, or choosing different journals due to an APC that made a particular outlet unaffordable. This code often indicates a sense of powerlessness.

*I have a well-funded lab and access to a decent amount of discretionary funding to pay for publishing fees, but then I often struggle because the highest-impact / most valued journals have the highest publishing fees, meaning that sometimes I have to choose between a high-valued venue and a venue I can afford.* (#170, UCB)

*I felt a bit trapped into the high fees ($3090) for this journal. We had originally submitted to a different (higher impact Nature family journal, which I think could have been worth the cost for its scope and recognition. But, after review, the editorial staff suggested we transfer to a more specialized Nature family journal. At that point, it would have been too time consuming and cumbersome to pull the paper and resubmit to a completely different journal, so we went ahead. But the fees for this journal do NOT match its standing or reputation, and I could have easily submitted this article to a no cost PLoS journal if I had realized earlier how much this journal would cost.* (#194, UTK)

#### Code: Grants don’t cut it.

This code was applied to responses that specifically indicate limitations in grant funding, whether grant funds were used to pay for the APC or not. This code often referred to grant funds not being enough to pay for everything needed to meet research or academic expectations. This code was not applied to acknowledgements that others lack grant funds to cover APCs (which would be coded *Diversity or Equity*). One theme that recurred in this code was the complicated logistics of paying APCs with grants and the various hurdles and barriers that caused authors.

*People with NIH [National Institutes of Health] and NSF [National Science Foundation] money get much more and are required to publish open access. USDA is not that case and all of my funding comes from that, leaving me to use valuable discretionary funding that could be used for other important purposes (equipment, equipment maintenance, etc.)* (#274, UMass)

*There is a Catch-22 when it comes to using grant funding to cover the APC. Typically we are submitting manuscripts for publication at least a year after our research funding expires. Therefore we can’t pay the APC from grant funds unless the NIH allows us to retain that money after we close out the grant.* (#95, Pitt)

#### Code: Academic obligation/expectation.

Responses coded for *Academic Obligation/Expectation* indicate a strong expectation or obligation to publish OA for professional advancement. These respondents mention pressures surrounding promotion, publication impact, student benefits, or to conform with disciplinary norms. This code is similar to *Restriction of Choice* but often without a specific decision point. It also often indicates a feeling of being trapped by expectations around the job.

*IF journals require publishing fees, researchers should be allotted money for this since it is a requirement of my job.* (#156, UCB)

*APCs should be supported by Dept./College/University because publishing is an expectation/requirement of their job and benefits the institution’s reputation.* (#221, UTK)

#### Code: Academic labor.

This code references responses about academic work (including peer review) that is often done without compensation, while a journal or publisher profits from APCs. These responses include references to rewards (such as a waived fee in exchange for labor) and volunteerism required by some journals.

*I agree with open access publishing, but I do not understand why the onus is on investigators to pay instead of publishers. Journals receive free peer review, pay next to nothing for editorial board stipends, receive ad revenue, and now have small publishing costs as print journals are dwindling. It would be reasonable if investigators covered only costs related to editorial stipends, copy editing, and online hosting (assuming that publishers will provide these data transparently).* (#40, Pitt)

*The publishing business is very difficult to understand. We, researchers, write and review the papers that are published for free. The publishing houses control the logistics and nothing else, for which we pay them. Then, the authors receive no royalties and the publishers get all the profits; that seems unfair.* (#152, UCB)

*We provide peer-review service, editorial service.. Journals APCs are legalized thevery [sic], esp. at high-end range.* (#327, UTK)

#### Code: Quality or rigor.

Several respondents mentioned concerns that OA reduces the quality or integrity of the research. This code often includes concerns about perverse incentives for publishers to accept papers of dubious quality from authors who can pay the fee, or with speculation that high Impact Factor journals or those that are perceived as elite are exploiting their perceived high-quality status to charge higher APCs.

*I don’t like the model where researchers pay a lot (in some journals) to publish their work – it creates bad incentives to publish bad research in some cases, and makes it so people with less financial backing cannot have their papers openly accessible in others.* (#164, UCB)

*Page charges and open access fees are being exploited by so-called elite journals because of their perceived status. Individuals are more than willing to pay $5-10K or more to publish in Nature and Cell title journals often because of careerism. Members of promotion and tenure committees or grant review panels are often so specialized that they cannot evaluate science outside of their narrow areas. So having the imprimatur of Cell or Nature in the journal title gives the allure of significance or importance. This is corrupting.* (#6, Pitt)

#### Presupposition of cost.

The coding process also initially identified a theme of *Presupposition of Cost*, meaning that the respondent conflated OA with an APC or other fee. Several respondents positioned payment for OA publication as an expected fact about the world, although about 71% of the journals in the Directory of Open Access Journals (DOAJ) do not charge article processing fees as part of their business model [15]. This is a linguistic phenomenon known as *presupposition:* speakers assume that readers know some information and reference that information when discussing other information. This presupposition that OA requires a publication fee has been noted in other research [16]. However, this theme was not used as a code in the final analysis of the APC Trap for two reasons: first, because this section of the survey was primarily answered by authors who had paid an APC for their publication (and, therefore, the existence of the cost was in fact true), and second, the wording of the survey question contained a presupposition of cost in itself (“Do you have any additional comments regarding authors’ ability to pay publishing fees for open access?”).

However, it remains essential to highlight this presupposition, as it reveals a trend in conversations and marketing surrounding OA. Conflating OA (a model for the availability and use of research articles) with APCs (a business model for publishing) can not only entrench beliefs about publishing options but also complicate or obfuscate the purpose of publishing OA [16]. The conflation of OA with APCs can make the APC Trap feel more like an Open Access Trap, erasing the potential of Diamond OA (that is, publishing without fees to the author or reader) and other models, tarnishing the practice of open research by equating it to complex market and privilege dynamics illustrated by the other components of the APC Trap.

Below are some examples of responses that contain a *Presupposition of Cost,* for illustrative purposes.

*This discussion is useless. There is a cost to publish a paper, and it does not matter what people feel they would like to pay for a paper. Somebody always paid, and with subscription journals, libraries pay for it – i.e., us.* (#198, UTK)

This example from respondent 198 contains a clear presupposition using the construction “There is”: “There is a cost to publish a paper”. This presentation of a fact presupposes that this is a truthful state of the world. Linking the subscription cost to publication costs is a type of presupposition often used in publisher marketing to suggest that if they are not generating revenue by charging a fee for a subscription, then they must charge a different fee. This response was not coded for any other codes associated with the APC Trap, indicating that this presupposition is not always linked to the APC Trap.

*Presupposition of Cost* can co-occur with other APC Trap codes. In the response below, there are two other aspects of the APC Trap: *Grants Don’t Cut It* and *Academic Obligation/Expectation*:

*[O]pen access is mandatory now in projects that are federally funded. The open access model drains the grant funds because my last paper cost more than 3k. The Open Access fund would only pay 1800 of this and there is no guarantee of this funding.* (#181, UTK)

This example contains the presupposition “[t]he open access model drains the grant funds.” While it is not clear if they are referring only to one example of an APC associated with the publication of an article draining grant funds, the reference to the “open access model” presupposes that the OA model itself, and not, for example, a certain journal or article, is to blame.

Presuppositions can also occur when expressing nuance around expectations for costs; these instances sometimes co-occur with the *Threshold of Reasonableness* APC Trap code:

*It is acceptable for open access journals to require publishing fees since they cannot obtain revenue from subscriptions or advertisements. However, those that only publish online should have lower fees (<$750) compared to those that publish online AND in print (<$1500).* (#60, Pitt)

This example contains a presupposition of revenue associated with journals, whether through subscriptions, advertisements, or publishing fees. In this case, the *Threshold of Reasonableness* code applied to this response means that while the respondent might accept the existence of a publishing fee, they still exhibit a sense of the APC Trap by expressing a preference for lower fees than the reported average.

### APC Trap overview

**Fig 1:**
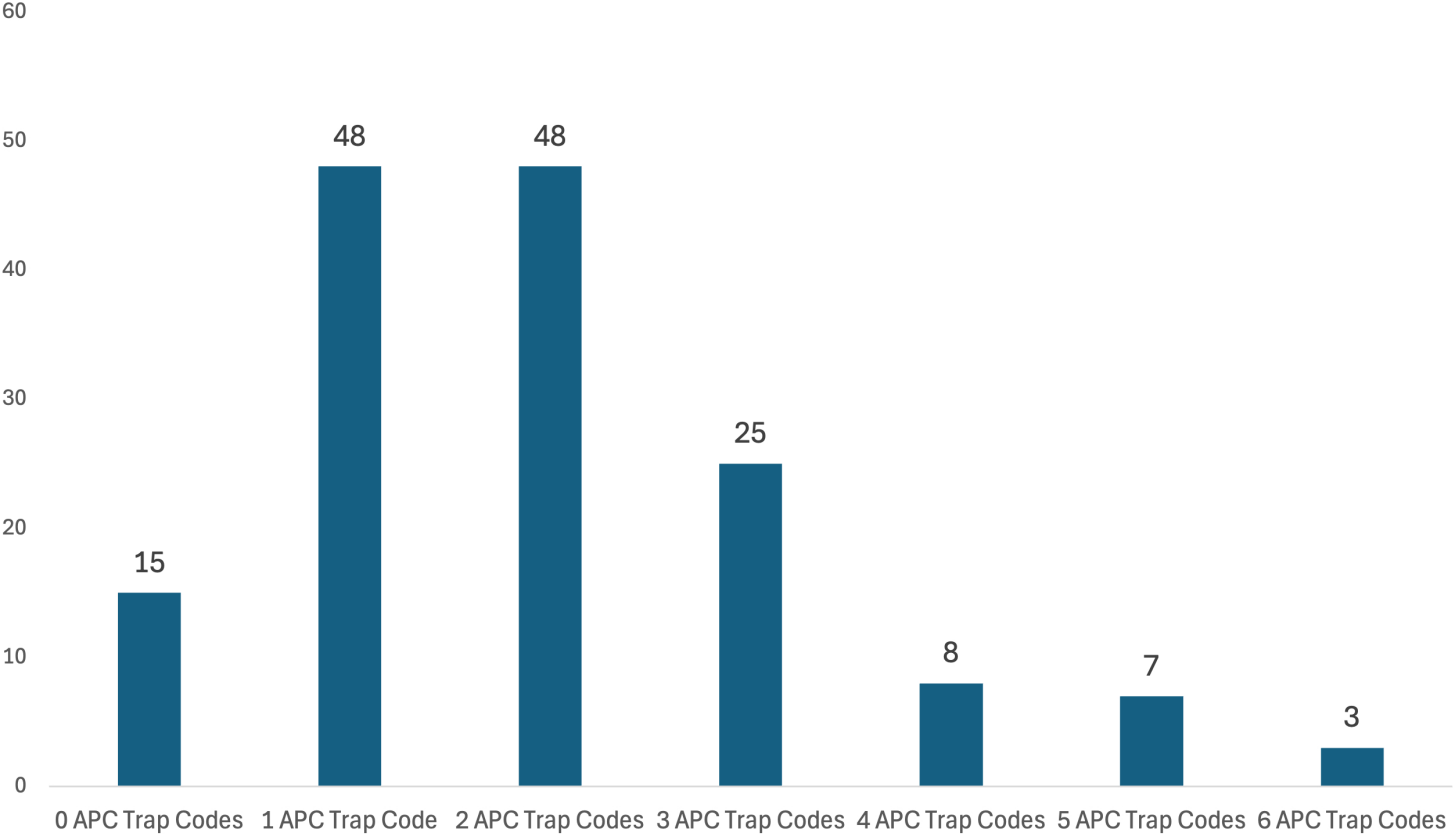
Responses by number of APC Trap codes.

After reaching a threshold of agreement for codes on all responses, results were analyzed to surface themes across the APC Trap codes. 90% of responses (n = 139) demonstrated at least one APC Trap code, indicating that the APC Trap is a phenomenon experienced by R1 authors in a wide array of contexts. Fig 1 shows the amount of responses grouped by the number of APC Trap codes associated with each response.

Most commonly, responses contained one or two APC Trap codes, with 62% of responses (n = 96) falling into these categories. Twenty-eight percent of responses (n = 43) contained three or more APC Trap codes. There were 15 responses which did not demonstrate any of the eight codes and 3 responses that demonstrated six codes, the most assigned to a single response.

Example response with no APC Trap Codes:

*“I like the idea of a University fund to support publication costs.”* #94, Pitt

Example response with six APC Trap Codes:

*“I really think these fees have gotten out of hand. Especially for junior researchers who may have little or no extramural funding, the fees present a dilemma. You have to publish to advance, but if you run out of money you won’t be able to continue your research, and you won’t advance. For the particular journal article that this survey referenced, the publisher offered to waive the fee if we submitted our work to that particular journal. This was the determining factor for me. If I hadn’t had to choose between one journal that didn’t charge a fee and a handful of others that would charge $3,000+ (!), I probably would have submitted to one of the other journals. But since I really felt like I needed to conserve resources, I opted for the journal that would waive the fee. Not a great system. I also really don’t understand where these fees go/what they are funding. They seem exorbitant for, what exactly? Putting my document into a template, loading it on a website, and adding it to some databases?”* #36, Pitt

Table 1 shows the total times each code was present in a response.

**Table 1:** Totals for each APC Trap code, and percentage of total responses.

APC Trap Code	Total	Percent of Total
Financial Concern	52	34%
Diversity or Equity	50	32%
Threshold of Reasonableness	46	30%
Restriction of Decisions	40	26%
Grants Don’t Cut It	35	23%
Academic Obligation/Expectation	33	21%
Academic Labor	28	18%
Quality or Rigor	20	13%

*Financial Concern* was the most common theme coded, with slightly more than a third (34%) of respondents expressing personal financial hardship. This may be due in part to respondents interpreting the first open-ended question about “authors’ ability to pay publishing fees” as a question about their own ability to pay. About a third (32%) of responses also included *Diversity or Equity* concerns, demonstrating that the inequity of APC-based OA models is top of mind for many R1 researchers.

### Demographics of the APC Trap

Demographic factors about respondents were gathered prior to the survey and were not based on respondent self-selection. There were some distinctive demographic variations in both the extent to which the APC Trap was experienced and the types of concerns which were most prominent.

Tables 2-4 display the average number of APC Trap codes based on these demographic factors, rounded to two decimal places.

**Table 2:** Average APC Trap codes by discipline.

Discipline	Average APC Trap Codes
Humanities and Social Sciences (HSS)	2.21
Natural Sciences and Engineering (NSE)	1.95
Health and Medicine (HM)	1.95

**Table 3:** Average APC Trap codes by institutional affiliation.

Institution	Average APC Trap Codes
University of Massachusetts (UMass)	2.59
University of Colorado Boulder (UCB)	2.06
University of Pittsburgh (Pitt)	1.97
University of Tennessee Knoxville (UTK)	1.59

**Table 4:** Average APC Trap codes by OA type.

Open Access (OA) Type	Average APC Trap Codes
Hybrid	2.21
Gold	1.94

HSS respondents demonstrated a 12.5% higher rate of APC Trap codes, on average, than the other two disciplinary categories. By institution, UMass responses demonstrated by far the largest number of APC Trap codes on average. Responses from authors of Hybrid articles demonstrated 13% more APC Trap codes, on average, than responses from authors of Gold articles.

**Fig 2:**
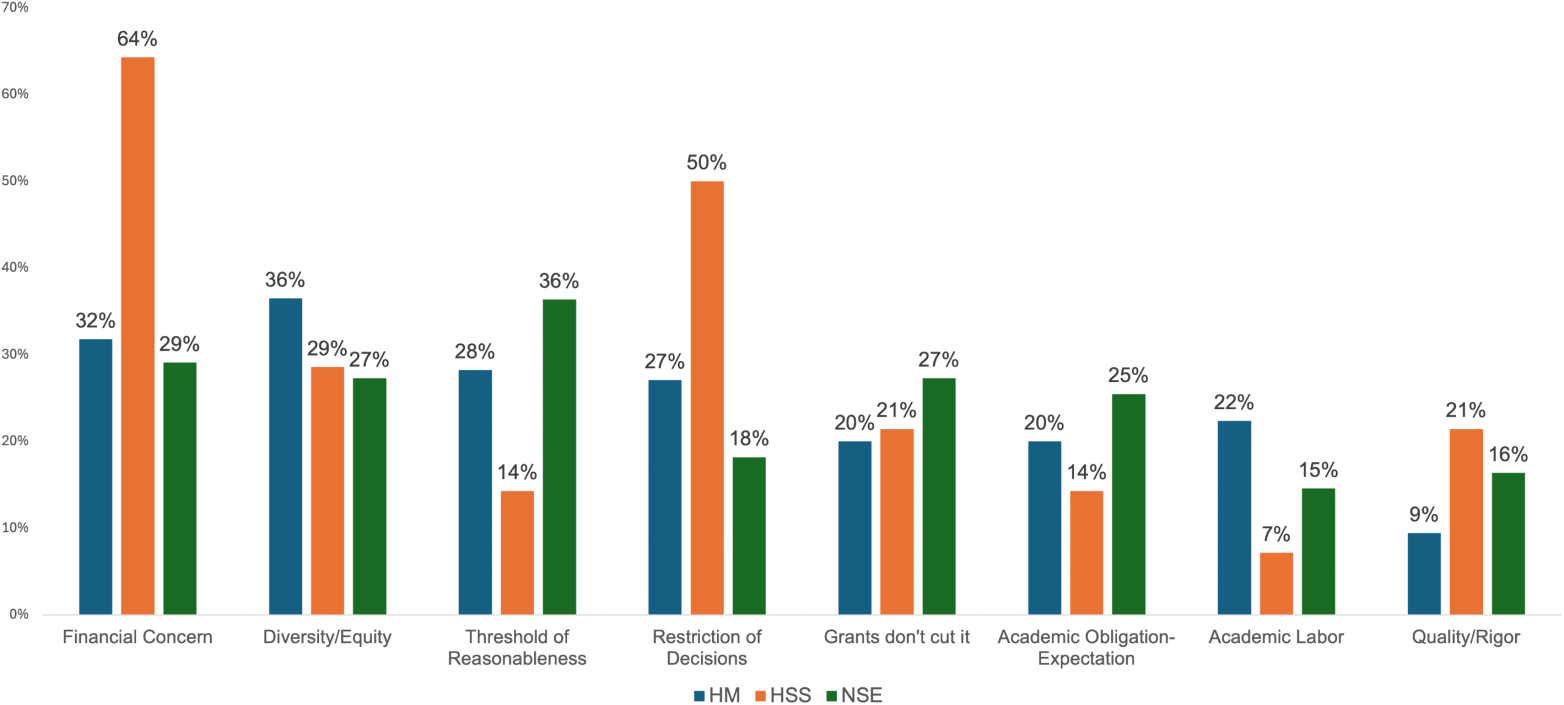
Percent of APC Trap codes by discipline.

Fig 2 shows a disciplinary breakdown of responses that include APC Trap codes. Respondents in HSS disciplines experienced the APC Trap more acutely than their peers, particularly in the areas of financing and decision-making around APCs.

*Financial Concern* was either the top or second-highest concern across all disciplines. HSS responses were twice as likely as HM and more than twice as likely as NSE responses to display *Financial Concern*. Similarly, HSS responses were almost twice as likely as HM and more than twice as likely as NSE responses to display a *Restriction of Decisions. Academic Labor* was by far the least likely code to appear in HSS responses.

For NSE respondents, *Threshold of Reasonableness* was the most common APC Trap code, with over one third (n = 20; 36%) of NSE responses demonstrating this code, followed by *Financial Concern*, *Diversity or Equity* and *Grants Don’t Cut It*. *Academic Labor* was the least common code in NSE responses.

*Diversity or Equity* was the most common APC Trap code for HM respondents, with more than a third (n = 31; 36%) of responses demonstrating this code, followed by *Financial Concern* and *Threshold of Reasonableness*. *Quality or Rigor* was the lowest concern demonstrated by HM responses.

**Fig 3:**
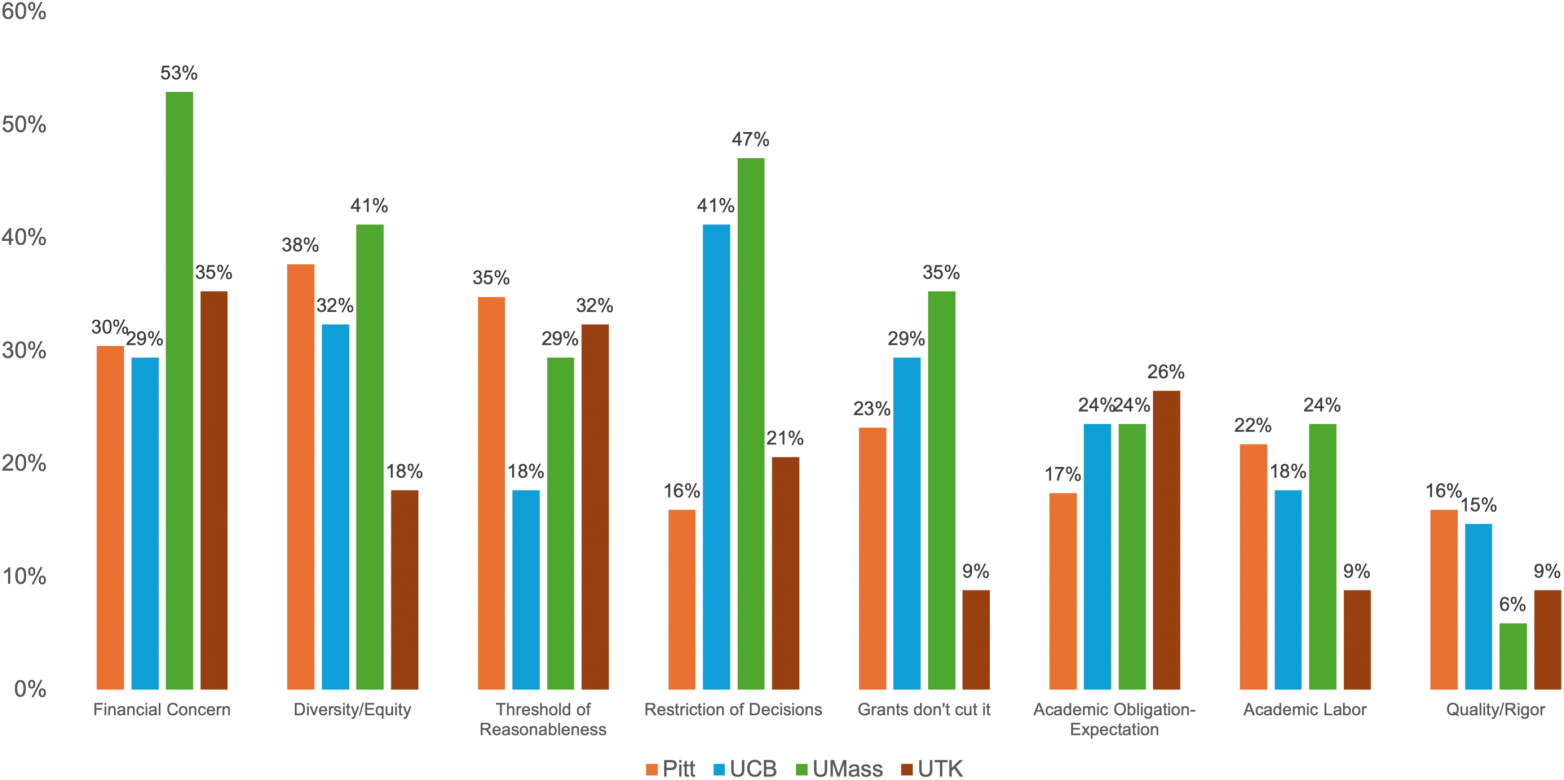
Percent of APC Trap codes by institution.

Fig 3 shows APC Trap codes separated by institution. More than half (n = 9; 53%) of UMass responses expressed *Financial Concern*, while nearly half (n = 8; 47%) expressed a *Restriction of Decisions* around OA funding. UMass responses were also the most likely to identify *Diversity or Equity* concerns around OA publishing. Out of the eight APC Trap codes identified for this study, UMass respondents demonstrated five of them more acutely than the other three institutions (*Financial Concern*, *Diversity or Equity*, *Restriction of Decisions*, *Grants Don’t Cut It*, and *Academic Labor)*.

For Pitt, *Diversity or Equity* (n = 26; 38%) was the biggest APC Trap element identified, followed closely by *Threshold of Reasonableness* (n = 24; 35%). Interestingly, while *Financial Concern* was the most identified APC Trap element in UTK responses, with over one-third (n = 12; 35%) of responses displaying *Financial Concern*, UTK also had significantly fewer responses indicating that *Grants Don’t Cut It* – 9%(n = 3), compared to a quarter or a third of responses at other institutions. For UCB, *Restriction of Decisions* was the most common APC Trap code identified (n = 14; 41%), followed by *Diversity or Equity* concerns (n = 11; 32%).

**Fig 4:**
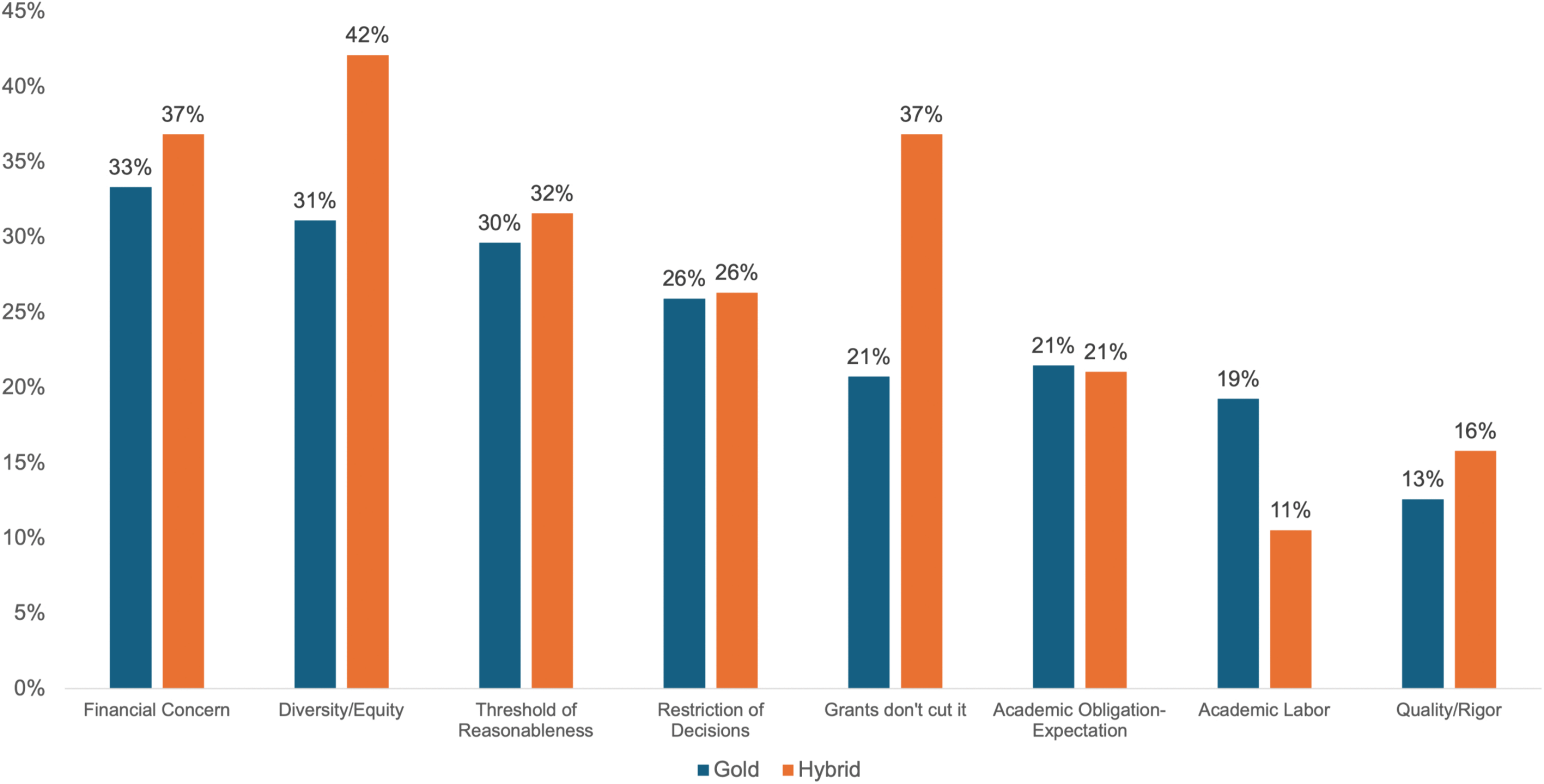
Percent of APC Trap code by journal OA type.

Fig 4 shows APC Trap codes separated by publication type, Gold or Hybrid OA. Authors of Hybrid articles were almost twice as likely as Gold OA authors to indicate in their responses that *Grants Don’t Cut It.* More to the point, respondents who published in Hybrid OA journals demonstrated every APC Trap code except *Academic Labor* more frequently than respondents who published in Gold OA journals.

### The APC Trap based on responses to other survey questions

**Fig 5:**
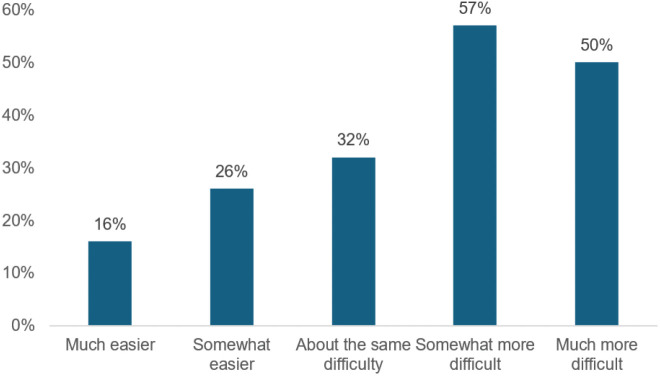
Percent of respondents’ comments coded as *Financial Concern* viewed by how respondents perceive their own ability to pay APCs relative to their peers.

Quantitative analysis of the codes alongside other survey questions demonstrates the layered nature of the APC Trap and strengthens the analysis of respondent sentiments. For example, Fig 5 shows that respondents who indicated it is more difficult for them to pay APCs are more likely to have left a comment coded as *Financial Concern*.

**Fig 6:**
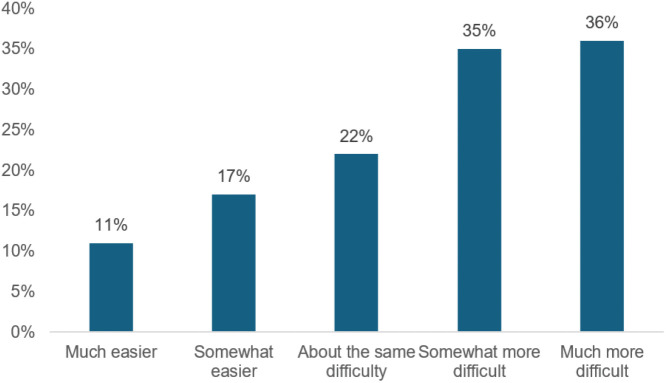
Percent of respondents’ comments coded for *Grants Don’t Cut It* viewed by how respondents perceive their own ability to pay APCs relative to their peers.

Conversely, when respondents said it was much *easier* for them to pay APCs, they also were less likely to leave a comment that was coded as *Grants Don’t Cut It* (Fig 6).

Other survey components reveal self-awareness; that is, respondents reported a moral quandary of participating in the system of paying APCs for OA even though they knew it was inequitable and made publishing more difficult for others. This sentiment surfaced when looking at the responses of those who reported that it was easier to pay APCs than others in their field.

**Fig 7:**
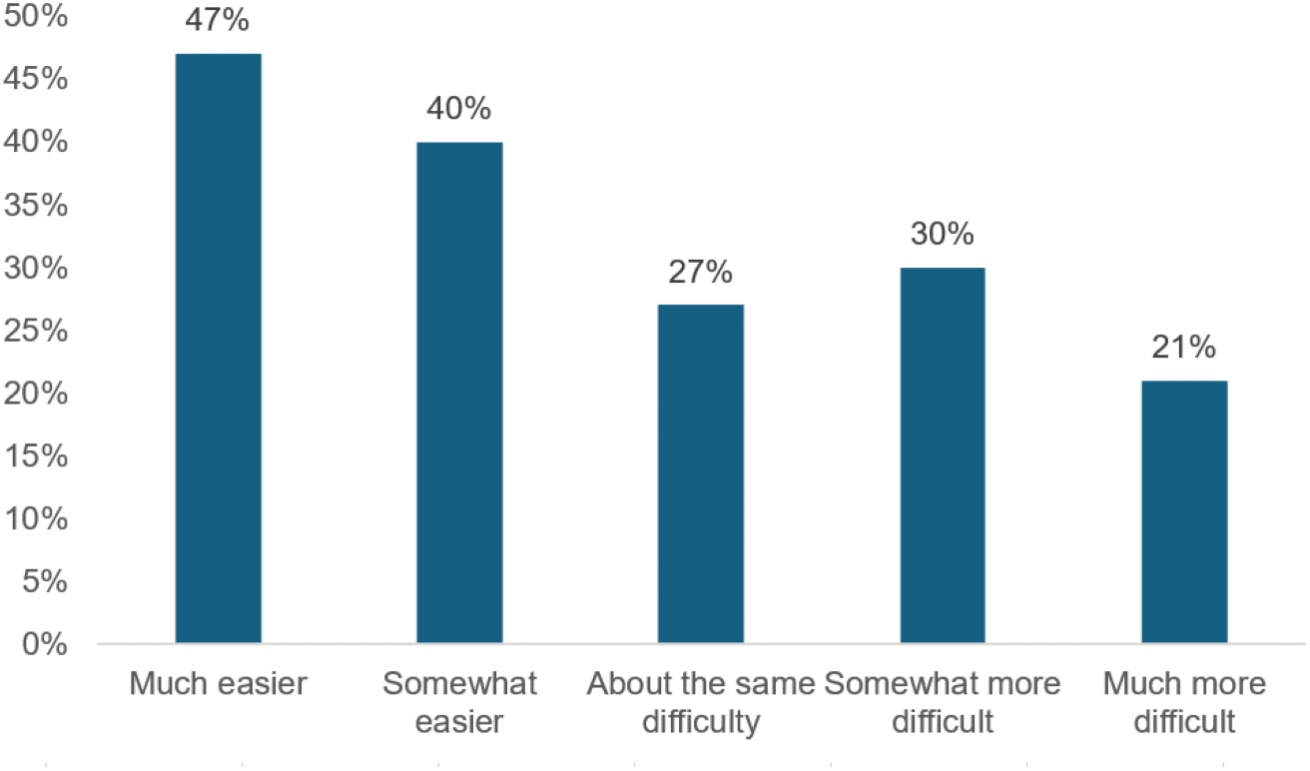
Percent of respondents’ comments coded for *Diversity or Equity* viewed by how respondents perceive their own ability to pay APCs relative to their peers.

Specifically, the easier that respondents thought it was for them to pay fees, the more their comments involved *Diversity or Equity* concerns. Fig 7 shows the upward trend for *Diversity or Equity* concerns as the self-reported ease of paying for APCs rises. This demonstrates a relationship between awareness of comparative ease and concerns about equity.

**Fig 8:**
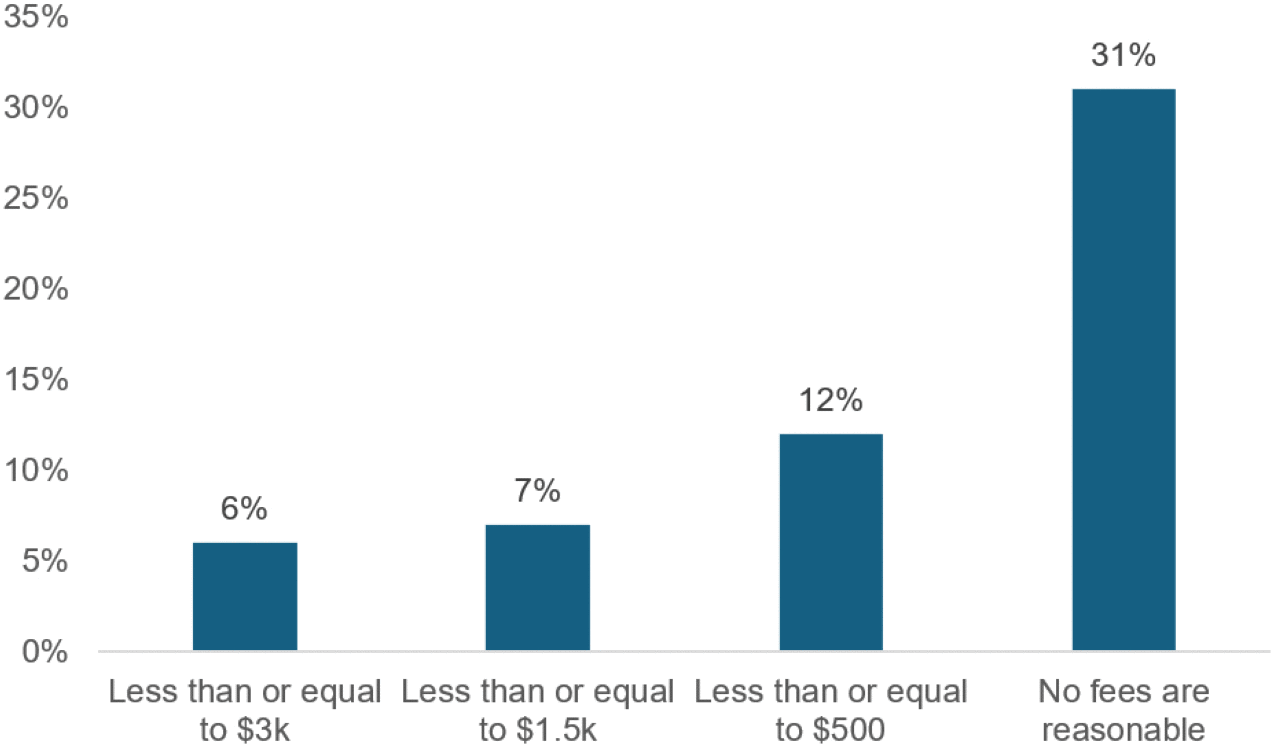
Percent of respondents’ comments coded for *Quality or Rigor* viewed by the amount respondents perceive is reasonable to pay for APCs.

Another moral quandary arose in the interaction between APCs and perceived ethics in publishing or academic conduct. Respondents who indicated that paying any APC amount is reasonable (that is, those who did *not* say “no fees are reasonable”), were much less likely to demonstrate concerns about *Quality or Rigor* (Fig 8). However, for respondents who indicated “No fees are reasonable”, *Quality or Rigor* was the second-most prominent code in their responses (second only to *Diversity or Equity)*, present in 31% of their responses.

### Cross-tabulation of the APC Trap codes

The analysis also examined the co-occurrence of codes within responses and found that some codes occurred together more often than others. Table 5 lists and highlights codes with a co-occurrence rate higher than 20%.

**Table 5:** Co-occurence matrix among APC Trap codes.

	Financial Concerns	Restriction of Decisions	Diversity or Equity	Grants Don’t Cut It	Academic Obligation / Expecta- tion	Quality or Rigor	Threshold of Reasonableness	Academic Labor
Financial Concerns	100%	37%	36%	18%	27%	8%	15%	8%
Restriction of Decisions		100%	13%	25%	24%	7%	16%	10%
Diversity or Equity			100%	13%	14%	21%	10%	11%
Grants Don’t Cut It				100%	21%	6%	11%	3%
Academic Obligation / Expectation					100%	8%	18%	7%
Quality or Rigor						100%	12%	9%
Threshold of Reasonableness							100%	21%
Academic Labor								100%

*Financial Concern* and *Restriction of Decisions* were the most likely to co-occur within a given response, with a 37% co-occurrence rate (n = 25 co-occurrence out of 67 coded for either). This is not surprising considering these are the two codes most likely to include personal anecdotes about difficulties with publishing fees. Examples of this cross-section of *Financial Concern* with *Restriction of Decisions* include:

*“I had to cut my salary in order to pay the increased fee for “mandatory” open access”* (#133, UCB)

*“I wrote an Opinion Piece, which cost about $400. The price determined what I wrote. I could not have afforded to submit an article.”* (#163, UCB)

Interestingly, *Financial Concern* and *Diversity or Equity* responses co-occurred at a similar rate (36%; n = 27 co-occurrence out of 75 coded for either). This is likely because the Code Book indicates that *Financial Concern* responses may involve the respondent identifying as a class or category of people with financial strain, while the Code Book’s instructions for *Diversity or Equity* are quite expansive and include references to any number of imbalances between different parties or stakeholders. Both codes deal with delineation between the self and others, of *me* and *them* (typically, more or less privileged researchers). Respondents with this co-occurrence were adept at understanding the wide variation of ability and need in funding OA publications. The following responses are examples of this unique crosstabulation:

“*The proportion of grant money that has to be set aside for APCs feels ridiculous, and unfair when that funding comes from public tax dollars and then is just shuttled straight to wildly profitable publishers. Also, publications in “good journals” are required to get funding in the first place, and this can place a strain on researchers who don’t yet have funding, but need to publish.”* (#232, UTK)

*“Open access fees really just fuel inequities in publications AND contribute to a lack of diversity in fully promoted and tenured professors at universities nationally. Those who have money can pay, be published, and get more citations, and those who don’t...well don’t. And then they are forced to move on to a different institution or career path. It’s a huge problem from a DEI perspective in academia.”* (#120, Pitt)

*Financial Concern* and *Academic Obligation/Expectation* also had a high co-occurrence rate (27%; n = 18 co-occurrence out of 67 coded for either). Responses invoke both personal financial strain as well as a sense of professional expectation or pressure to publish in *specific* journals, whether for the sake of the respondent’s academic tenure, promotion or job retention, or for the professional recognition of students who hope to become full-fledged researchers in the future. This co-occurrence is a demonstration of the APC Trap at work, whereby a sense of obligation is strong enough to override conflicting and often unsustainable financial strain:

*“I would estimate that I’m paying $10-15k per year in publication fees because so many journals in my field have switched to open access and I want to make sure students are publishing in places that keep them competitive for future research careers. UMass right now will partially subsidize one of those publications (but I need help with 4-5 pubs per year). It is completely unsustainable and I have no idea how I will continue to pay for publications.”* (#257, UMass)

Notably, both *Financial Concern* and *Academic Obligation/Expectation* were the most frequently co-occurring codes. Their repeated appearance in complex responses also serves to highlight a major competing stress – financial burden versus perceived career assessment expectations – that is apparent throughout much of this analysis.

*Restriction of Decisions* is a code that frequently co-occurred with other codes. The combination of *Restriction of Decisions* and *Grants Don’t Cut It* co-occur in 25% of responses (n = 15 co-occurrence out of 60 coded for either). This points to the strain multiple grant recipients expressed in their responses – even with grant funding, publishing decisions may feel forced, unsustainable, or, in the words of this researcher below, “absurd”:

*“Fees of $6000 to $10000 to publish after slow reviews and turnaround times by the journal are absurd. The system is broken yet we are often required or encouraged to publish in open access journals by NIH et al but we do not have the funds in our limited budget to cover this. The fees are not sustainable with the current level of support by NIH and non-profits. These fees may eat up as much as 10-20% of a small non profit grant. How are we supposed to cover our salaries, our lab salaries, pay for space and supplieds [sic], and then also pay these absurd fees?”* (#5, Pitt)

*Restriction of Decisions* also co-occurred with *Academic Obligation/Expectation* in 24% of responses (n = 14 co-occurrence out of 59 coded for either). These codes overlap in the Code Book in the sense that academic obligations often create real or perceived restrictions in publication decisions by authors. When there is an expectation within the field of study or the institution to publish OA or within certain high-impact journals, some of which charge APCs, this creates layered tension and frustration. Authors might feel obligated to pay fees to advance their careers, even if they might not agree on moral grounds or if it limits their decision-making. The following response demonstrates this quandary:

*Ability to pay fluctuates greatly depending on grant funding. But so long as the “prestige” associated with these journals is a key factor in evaluating faculty members, its [sic] hard not to [publish] there.* (#82, Pitt)

The co-occurrence of *Threshold of Reasonableness* and *Academic Labor* (21%, n = 13 out of 61 coded for either) indicates that those aware of the free labor provided in peer review contributions are particularly frustrated by the costs of OA publishing. Finally, the co-occurrence of *Diversity or Equity* and *Quality or Rigor*, which is the only co-occurring match above 20% for *Quality or Rigor,* suggests that those aware of the exclusion of disenfranchised authors may be simultaneously concerned that such exclusion can result in a reduction of overall quality/rigor in research and scholarship (n = 12 out of 58 coded for either).

## Discussion

Throughout the analysis of the APC Trap codes, it became apparent that there is a rich and multi-layered interplay across institutions, disciplines, publication type, as well as across the different codes, making each respondents’ experience of the APC Trap unique. However, some general trends can be discerned between demographic groups of respondents.

For example, HSS respondents were more likely to experience the APC Trap and experience it to a higher degree (multiple APC Trap codes) than other disciplines. It is likely this group experiences more financial pressure and fewer alternative funding options than their peers in this study because HSS authors are less likely to have available grant funding [17]. OA journals in HSS fields are also more likely to be Hybrid journals, which have significantly higher average APCs, placing additional financial pressure on authors [18]. This suggests that lack of institutional funding and broad OA initiatives likely impacted respondents who felt more financial strain or a restriction of decisions when paying these costs.

HSS respondents were also twice as likely to express personal concerns of OA publishing (*Financial Concern* and *Restriction of Decisions)*, while NSE and HM were more likely to express systematic concerns (*Threshold of Reasonableness*, *Diversity or Equity*, *Academic Labor*). This suggests that the APC Trap exists on a kind of spectrum. If an author can’t pay to begin with, their concerns are more localized, personal, and focused on how to participate within the system. If an author can pay more easily and holds a comparatively privileged status, concerns shift to broader inequities or systemic issues in scholarly publishing, including concerns about the inequities that APC models can reinforce in scholarly publishing. The broad concerns about *Diversity or Equity* that appeared in these findings are a key feature of the APC Trap as experienced by relatively privileged, R1 authors.

Though all respondents in this study were researchers at R1 institutions in the United States, there were also some trends gleaned across institutional affiliation. The first analysis of this study demonstrated that most APCs were paid through grants, yet authors at institutions with fewer alternative funding sources (e.g., departmental or library funds) appear to experience additional and compounded strain over the payment of APCs [11]. UMass, which had fewer alternative OA funding resources than the other institutions in this study, had the highest proportion of respondents express APC Trap codes in three codes with a financial angle: *Financial Concern, Grants Don’t Cut It,* and *Restriction of Decisions.* Similarly, even though UTK authors were very unlikely to indicate that *Grants Don’t Cut It* (9%; n = 3), *Financial Concern* was still at the forefront for many UTK respondents. These findings indicate that even at very well-resourced institutions, finances around OA publishing are precarious, and safety nets, where they exist, might still be quite thin and unstable.

This precariousness is further evinced when looking at how respondents rate their own ability to pay based on the APC Trap codes identified in their responses. Unsurprisingly, those who report it is easier for them to pay APCs are less likely to report that grant funds are lacking. Researchers who can pay APCs with grants may have the easiest time paying these fees, as they don’t have to search of external funding sources. However, even those who are able to set aside funding through grants may report feeling other pressures, and availability of this funding does not mean a researcher isn’t having to make difficult decisions about how money is allocated. Support of any kind is not without costs, as the following response shows:

*“The fees keep increasing as my obligations decrease the time I have to write grants to pay for these costs! Plus it stinks to spend a month of a grad student’s salary on OA. Supporting trainees is where I’d prefer to spend money.”* (#139,UCB)

Some respondents, such as this one, recognize that participating in paying APCs presents a moral quandary in which one benefit was exchanged at the expense of another. This quandary also has real, negative implications for research and the scholarly record. When researchers are unable to pay for lab assistants because they must cover publication costs, studies may take longer or become less rigorous. Restricted choices and exclusions also cause the scholarly record to suffer, such as when a researcher submits an opinion piece rather than an article due to price, but the article is more appropriate for their work. Weighing these costs and benefits increases the bind of the APC Trap experienced by respondents.

Additionally, the alignment between respondents who indicated “No fees are reasonable” and who had concerns about *Quality or Rigor* may indicate a conflation between APCs and a low-quality, review-minimal “pay to publish” model, which is a sentiment sometimes expressed in the responses themselves:

“*I think open access tho [sic] well intentioned is a disaster. Authors should not be in the position of pay to play. Even for those who do not want to participate in the APC system and who think that fees are completely unreasonable, they indicate a recognition that those who do participate are setting up an ethics problem in publishing related to the pressure to publish low quality materials.”* (#256, UMass)

Responses such as these exhibit an exasperation with APC models of OA publishing more generally. The issue is not just with payment but that these models may be lower in quality and less trustworthy. These viewpoints may be rooted in well-circulated misconceptions of OA that reify traditional subscription-based scholarly publishing, or they could be rooted in a desire to return to the largely Diamond-model origins of OA publishing. Without digging further into these respondents’ perceptions, it is not possible from this study to know the precise source of these sentiments. However, the sentiments themselves point to deep and complex discomfort with the status quo of scholarly publishing, in which OA is contributing just as much to the problem as to potential solutions.

### The messy knot of the APC Trap

This analysis and the examples provided show that the APC Trap is not a simple or straightforward phenomenon and is not monolithic or homogeneous across authors who may all be perceived as privileged, at least to some degree. The extent of its impact and the level at which any one researcher might experience the APC Trap is highly dependent on an individual’s positionality. This includes their positionality within their discipline, within their institution, as well as their perceived positionality within a global and national context. There are likely many other points of positionality that could influence a researcher’s experience of the APC Trap, such as being tenured or non-tenured, which were not variables in this study.

The key, unifying feature of the APC Trap is its resulting dissonance or bind, expressed as a frustration and acute sense of injustice within the system of scholarly publishing coupled with an obligation or sense of moral responsibility to participate in such a system. This bind creates a sense of powerlessness to change the system, or to influence its direction, which only serves to pull the knot tighter.

## Limitations

The most prominent limitation of this study is that the survey instrument was not intentionally designed to facilitate a robust qualitative analysis. The initial aim of the study was to identify funding sources for APCs and perception of authors’ ability to pay related to peers and to authors at less advantaged institutions. The open-ended questions at the end of the survey were intended to capture additional thoughts regarding authors’ ability to pay these fees to supplement the quantitative analysis, not to measure the APC Trap. However, once the survey was completed, it was clear that many of the 48% of respondents who provided usable open-ended responses were expressing frustrations and anxiety about their experiences with OA publishing which went far beyond the original scope of the study. Future research might find ways to better target and measure these experiences that are observed obliquely.

Additionally, while many respondents used the open-ended questions to suit their own purposes, the framing of the questions themselves undoubtedly led many survey participants to respond in a particular manner. The first question asks respondents specifically to speak to financial concerns related to APCs, and the second question also favors commentary about payment and cost because those were the topics of the previous multiple-choice questions in the survey. Questions targeting other aspects of OA publishing likely would have revealed additional APC Trap codes not captured by this study and may have impacted the degree to which financial concern and other themes related to finances (*Grants Don’t Cut It*, *Threshold of Reasonableness*) were observed in the analysis. As noted previously, this concern about leading questions is also the reason *Presupposition of Cost* is excluded from the final analysis.

Lastly, this study was designed to capture the sentiments of authors at very high research-intensive (R1) public institutions based in the U.S. As a study with a purposive sample, the findings are not representative of other institutions or nations; however, some limited, broader implications of the study are detailed in the Conclusion section below.

### Limitations of sentiment analysis tools

Automated sentiment analysis was attempted to aid in contextualizing the respondents’ points of view and concerns. Two different tools were tested: NVivo’s Auto Sentiment Coding and SentiStrength [19]. Unfortunately, for this dataset, the results and analysis from these tools was unusable. These are reported here because they represent a potential limitation to this study and future avenue for investigation.

The primary drawback was that each tool often flagged responses for simultaneously positive and negative sentiments. For example, NVivo categorized “moderately positive” responses that expressed support of OA in principle with concerns about inconsistent fees and unequal burdens on underfunded authors. SentiStrength offered even more mixed results because it used a numerical scoring approach, assigning values to individual words in each response. For several responses that all authors agreed to be strongly negative, SentiStrength assigned scores of 0 or even positive values due to simplistic keyword associations with isolated words such as “profit” or “enjoy,” without accounting for the context of the response.

The results from these experiments highlight a limitation of automated sentiment tools when applied to complex, nuanced topics like academic publishing models. Especially when used outside of their core training data, they persistently mis-characterize the intricacies of human sentiment, such as sarcasm, rhetorical structure, or compound sentiments [20]. These features appeared often in the analyzed dataset, since the respondents expressed strong emotions with subject matter that is deeply complex and intertwined with power, equity, and institutional critique. If automated tools improve, a sentiment analysis may be a promising avenue for future analysis.

## Conclusion and broader implications

Since APCs started gaining traction as a financial model for OA publishing at the beginning of the century, scholars, librarians, authors, and some publishers have raised serious concerns about the long-term sustainability of the model. There have also been concerns about how APCs re-entrench uneven and inequitable participation in knowledge sharing and distribution.

This study demonstrates concerns by researchers in the U.S. but may have global implications which could be explored in future studies. The concerns observed in this study manifest in the form of a phenomenon coined here as the APC Trap, which is characterized by eight overlapping and interacting themes. The eight codes of the APC Trap identified in this study mean that its actualization is unique from one researcher to the next. The limited scope of this purposive sampling study to four R1 institutions in the U.S. also means that there are undoubtedly valences of the APC Trap experienced by authors outside this study. However, the findings demonstrate how this phenomenon surfaces in the lived experiences of this set of researchers.

This study raises questions about broader implications. First, whether the APC Trap is experienced in wider, global contexts. Its pervasiveness within this relatively small sample suggests the APC funding model faces many challenges to adoption and acceptance in the U.S. even when a strong ethical and ideological drive to publish open access is present. Second, whether the agonized viewpoints expressed by researchers from some of the most well-resourced universities in the U.S. are accentuated in other regions and socioeconomic contexts.

APCs are not the only model for open access publishing, and a variety of alternative models have sought to repair concerns around cost and equity. Plan S attempted to propose an APC cap as a form of price control. Transformative agreements were proposed to bridge subscriptions to OA publishing models and to formalize libraries’ support of OA publishing on behalf of authors. SCOAP3 created a collective governance model of over 3,000 participating institutions to shift cost barriers away from individual authors. Subscribe to Open (S2O) models rely on reaching a threshold number of subscribing institutions to unlock research for everyone. While there are too many models to enumerate in full here, what is important to note is that the vast majority of proposed alternatives do not substantially reduce the price of OA publishing, but merely obscure the price of publishing from authors, hiding them behind a new (but not altogether different) administrative veneer. Many of these alternative models do not solve the APC Trap but merely redirect its impact onto other academic and administrative entities, including libraries.

Transformative or read-and-publish agreements continue to flourish, and several of the institutions in this study have accumulated more of these agreements since this survey was conducted. Because these agreements obscure prices but also favor full waivers for Hybrid journals rather than Gold journals, it will be interesting to see how these agreements shift the publishing landscape and influence expressions of the APC Trap. As APC prices continue to rise in an austere funding environment, authors may feel their options for OA publishing continue to restrict to Hybrid journals as they seek institutional support via these agreements.

For all their faults, APCs – as opposed to the alternative approaches above – render the prices of OA publishing visible and palpable to authors. Through APCs paid directly by authors, the cognitive dissonance caused by the current scholarly publishing landscape is readily apparent:

*“[T]he system is obviously bonkers, in that we create the products, review others’ products for free, and then pay to get the products published. What a world!”* (#165, UCB)

*“The whole system is a cash cow, and we pay and pay and pay. And worse, it’s literally a requirement for our academic success. And that is before you investigate equity concerns…”* (#254, UMass)

*“It feels like legalized extortion. I am a captive audience. I need to publish my work both because that is how I get promoted and remain competitive for grant funding… It just feels wrong.”* (#52, Pitt)

To alleviate the experience of the APC Trap for authors, an obvious solution may be to subsume those prices into new subscriptions, transformative agreements, and collective funding initiatives. While these models may temporarily avert the APC Trap by obscuring the prices of publishing from authors, they do not solve the problem of the APC Trap. Rather, other models may simply delay the reckoning with publication prices and entrench existing inequities by redirecting ethical and financial burdens. When scholars and institutions must finally confront the trap, will we be bound too tight to break free?
