# Correction: “The system is obviously bonkers”: The APC Trap and the bind of scholarly publishing across four research intensive institutions in the U.S.

**DOI:** 10.1371/journal.pone.0358346

**Published:** 2026-09-11

**Authors:** Melissa H. Cantrell, Rachel Caldwell, Jennifer A. Mezick, Matthew Estill, Lauren B. Collister

The corresponding author’s email address is incorrect. Melissa H. Cantrell’s email address is: mcantr19@utk.edu.

In the Discussion section, there is an error in the second quote. The correct quote is: I think open access tho [sic] well intentioned is a disaster. Authors should not be in the position of pay to play. The Library of Congress needs to publish scientific papers at cost, with the funds taken from an agency wide tax. For profit scientific publishers need to be put out of business.“(#256, Umass).
