# Supplementary material for: “The system is obviously bonkers”: The APC Trap and the bind of scholarly publishing across four research intensive institutions in the U.S
Source: PLoS One. 2026 Jul 1;21(7):e0351430. doi: 10.1371/journal.pone.0351430 (PMC13322547; doi:10.1371/journal.pone.0351430)
Supplement: Attachment — Submitted filename: PONE-D-25-47145_R1_w. a few reviewer comments.pdf [file pone.0351430.s003.pdf]

PLOS ONE

# "The system is obviously bonkers": The APC Trap and the bind of scholarly publishing across four research intensive institutions in the U.S.

--Manuscript Draft--

| | |
|---|---|
| Manuscript Number: | PONE-D-25-47145R1 |
| Article Type: | Research Article |
| Full Title: | "The system is obviously bonkers": The APC Trap and the bind of scholarly publishing across four research intensive institutions in the U.S. |
| Short Title: | The APC Trap and the bind of scholarly publishing |
| Corresponding Author: | Melissa Hart Cantrell, MA, MLIS<br>University of Colorado Boulder<br>Boulder, CO UNITED STATES OF AMERICA |
| Keywords: | Open access; scholarly publishing; article processing charges; APCs; Library and information science |
| Abstract: | This study analyzes the cognitive dissonance displayed by authors at research intensive institutions in the U.S. with regard to the scholarly publishing of Open Access (OA) publications. A qualitative analysis of 154 open-ended responses was conducted. Eight themes were identified, then each response was coded for the themes. The themes themselves coalesce around a phenomenon the authors of this study term the article processing charge (APC) Trap, in which privileged and well-resourced researchers in the U.S. experience highly contradictory sentiments about OA publishing. The results of the cognitive dissonance characteristic of the APC Trap are strong feelings of conflict as well as powerlessness. The qualitative analysis reveals the APC Trap as a deeply layered phenomenon, whose manifestation is dependent on positionality, including demographic features and interactions between APC Trap themes. While much has been made of the failings of the APC model of OA publishing for under-resourced researchers, this study reveals the ways in which this model is unsustainable even for privileged researchers and countries most well-positioned to participate within it. |
| Order of Authors: | Melissa H. Cantrell |
| | Rachel Caldwell |
| | Jennifer A. Mezick |
| | Matthew Estill |
| | Lauren B. Collister |
| Response to Reviewers: | A letter detailing the revisions required and our response to them is included in the attached documents for this submission. |
| Additional Information: | |
| **Question** | **Response** |
| **Financial Disclosure**<br><br>Enter a financial disclosure statement that describes the sources of funding for the work included in this submission. Review the submission guidelines for detailed requirements. View published research articles from *PLOS ONE* for specific examples. | The author(s) received no specific funding for this work. |

*Powered by Editorial Manager® and ProduXion Manager® from Aries Systems Corporation*

| | |
|---|---|
| This statement is required for submission and **will appear in the published article** if the submission is accepted. Please make sure it is accurate.<br><br>**Funded studies**<br>Enter a statement with the following details:<br>• Initials of the authors who received each award<br>• Grant numbers awarded to each author<br>• The full name of each funder<br>• URL of each funder website<br>• Did the sponsors or funders play any role in the study design, data collection and analysis, decision to publish, or preparation of the manuscript?<br><br>Did you receive funding for this work? | |
| **Competing Interests**<br><br>Use the instructions below to enter a competing interest statement for this submission. On behalf of all authors, disclose any competing interests that could be perceived to bias this work—acknowledging all financial support and any other relevant financial or non-financial competing interests.<br><br>This statement is required for submission and **will appear in the published article** if the submission is accepted. Please make sure it is accurate and that any funding sources listed in your Funding Information later in the submission form are also declared in your Financial Disclosure statement.<br><br>View published research articles from *PLOS ONE* for specific examples. | The authors have declared that no competing interests exist. |

| | |
|---|---|
| **NO authors have competing interests**<br><br>Enter: *The authors have declared that no competing interests exist.*<br><br>**Authors with competing interests**<br><br>Enter competing interest details beginning with this statement:<br><br>*I have read the journal's policy and the authors of this manuscript have the following competing interests: [insert competing interests here]*<br><br>* typeset | |
| **Ethics Statement**<br><br>Enter an ethics statement for this submission. This statement is required if the study involved:<br><br>• Human participants<br>• Human specimens or tissue<br>• Vertebrate animals or cephalopods<br>• Vertebrate embryos or tissues<br>• Field research<br><br>Write "N/A" if the submission does not require an ethics statement.<br><br>General guidance is provided below. Consult the submission guidelines for detailed instructions. **Make sure that all information entered here is included in the Methods section of the manuscript.** | The study was reviewed by the University of Colorado Review Board and accepted under the review category Exempt. IRB Protocol Number: 23-0216 |

| | |
|---|---|
| **Format for specific study types**<br><br>**Human Subject Research (involving human participants and/or tissue)**<br>• Give the name of the institutional review board or ethics committee that approved the study<br>• Include the approval number and/or a statement indicating approval of this research<br>• Indicate the form of consent obtained (written/oral) or the reason that consent was not obtained (e.g. the data were analyzed anonymously)<br><br>**Animal Research (involving vertebrate animals, embryos or tissues)**<br>• Provide the name of the Institutional Animal Care and Use Committee (IACUC) or other relevant ethics board that reviewed the study protocol, and indicate whether they approved this research or granted a formal waiver of ethical approval<br>• Include an approval number if one was obtained<br>• If the study involved *non-human primates*, add *additional details* about animal welfare and steps taken to ameliorate suffering<br>• If anesthesia, euthanasia, or any kind of animal sacrifice is part of the study, include briefly which substances and/or methods were applied<br><br>**Field Research**<br><br>Include the following details if this study involves the collection of plant, animal, or other materials from a natural setting:<br>• Field permit number<br>• Name of the institution or relevant body that granted permission | |
| **Data Availability**<br><br>Authors are required to make all data underlying the findings described fully available, without restriction, and from the time of publication. PLOS allows rare exceptions to address legal and ethical concerns. See the PLOS Data Policy and FAQ for detailed information. | Yes - all data are fully available without restriction |

| | |
|---|---|
| A Data Availability Statement describing where the data can be found is required at submission. Your answers to this question constitute the Data Availability Statement and **will be published in the article**, if accepted.<br><br>**Important:** Stating 'data available on request from the author' is not sufficient. If your data are only available upon request, select 'No' for the first question and explain your exceptional situation in the text box.<br><br>Do the authors confirm that all data underlying the findings described in their manuscript are fully available without restriction? | |
| **Describe where the data may be found in full sentences. If you are copying our sample text, replace any instances of XXX with the appropriate details.**<br><br>• If the data are **held or will be held in a public repository**, include URLs, accession numbers or DOIs. If this information will only be available after acceptance, indicate this by ticking the box below. For example: *All XXX files are available from the XXX database (accession number(s) XXX, XXX.).*<br>• If the data are all contained **within the manuscript and/or Supporting Information files**, enter the following: *All relevant data are within the manuscript and its Supporting Information files.*<br>• If neither of these applies but you are able to provide **details of access elsewhere**, with or without limitations, please do so. For example:<br><br>*Data cannot be shared publicly because of [XXX]. Data are available from the XXX Institutional Data Access / Ethics Committee (contact via XXX) for researchers who meet the criteria for access to confidential data.*<br><br>*The data underlying the results presented in the study are available from (include the name of the third party* | Survey instruments and data for the study are available at https://github.com/parnopaeus/oalanguage/tree/master/APCStudy |

| | |
|---|---|
| *and contact information or URL).*<br>• This text is appropriate if the data are owned by a third party and authors do not have permission to share the data.<br><br>* typeset | |
| Additional data availability information: | |

Manuscript
Click here to access/download;Manuscript;APCTrap_PLOSOne_Manuscript.d

# "The system is obviously bonkers": The APC Trap and the bind of scholarly publishing across four research intensive institutions in the U.S.

Melissa H. Cantrell[1¶]*, Rachel Caldwell[2¶], Jennifer A. Mezick[3¶], Matthew Estill[4¶], Lauren B. Collister[5¶]

[1] University Libraries, University of Colorado Boulder, Boulder, Colorado, United States of America

[2] University Libraries, University of Massachusetts Amherst, Amherst, Massachusetts, United States of America

[3] University Libraries, University of Tennessee Knoxville, Knoxville, Tennessee, United States of America

[4] University Library System, University of Pittsburgh, Pittsburgh, Pennsylvania, United States of America

[5] Invest in Open Infrastructure

* Corresponding author
¶ These authors contributed equally to this work

E-mail: melissa.cantrell@colorado.edu

# Abstract

This study analyzes the cognitive dissonance displayed by authors at research intensive institutions in the U.S. with regard to the scholarly publishing of Open Access (OA) publications. A qualitative analysis of 154 open-ended responses was conducted. Eight themes were identified, then each response was coded for the themes. The themes themselves coalesce around a phenomenon the authors of this study term the article processing charge (APC) Trap, in which privileged and well-resourced researchers in the U.S. experience highly contradictory sentiments about OA publishing. The results of the cognitive dissonance characteristic of the APC Trap are strong feelings of conflict as well as powerlessness. The qualitative analysis reveals the APC Trap as a deeply layered phenomenon, whose manifestation is dependent on positionality, including demographic features and interactions between APC Trap themes. While much has been made of the failings of the APC model of OA publishing for under-resourced researchers, this study reveals the ways in which this model is unsustainable even for privileged researchers and countries most well-positioned to participate within it.


# Introduction

This paper examines the complex attitudes and experiences of researchers regarding article processing charges (APCs) in open access (OA) publishing. The APC model's introduction is usually attributed to BioMed Central in the year 2000 as a means to support financial sustainability for publishers who offer open access to scholarly articles in lieu of subscription revenues [1]. The problematization of the APC-based business model began shortly after its introduction. Authors initially approached APCs as a binary choice of whether to pay or not: "once authors are willing or able to pay an APC, [...] they are willing to pay them with little regard to the size of an APC" [2]. Publishers report pricing APCs based on journal prestige rather than production costs, indicating little market control over hyperinflation [3]. APC hyperinflation was

documented at 5-10% annual increases with little impact on growth at major publishers or evidence for need to increase prices [2].

Beyond the pricing models, literature has consistently highlighted how APCs in general create "barriers" excluding researchers from less well-resourced institutions and countries [1,2,4,5] and reinforcing existing inequities [3,6,7,8]. Journal editors view APCs as barriers to inclusivity, expressing concern that APCs replicate financial inequalities and disadvantage under-resourced authors, particularly from the Global South or smaller institutions [9]. While authors at institutions with OA budgets viewed OA as complementary to other factors like reputation and editorial practices, those without OA budgets saw APCs as major deterrents to publishing and making their research widely available. In a 2021 study of researchers from India, authors with institutional support viewed OA favorably, with one researcher explaining: "Sometimes I think about open access as my first choice...because I'm coming from a low-resource country. And I know how people there suffer in finding the information." Conversely, unsupported authors reported: "I simply won't consider those journals because I just can't [pay] those fees...even if it's probably the best journal for me" [5]. While researchers value OA in principle, cost remains a major deterrent when not covered by grants or institutions [6].

This study explores the navigation of the open access publishing landscape by authors who encountered APCs. In previous work, the authors conducted a survey to understand the resources that authors used to pay for APCs [10]. In addition to the quantitative findings, the responses to open-ended questions in the survey demonstrated cognitive dissonance among researchers who show a strong willingness to publish OA while viewing APC prices as increasingly unreasonable and unfeasible. These findings aligned with observations of the "APC effect" introduced by Klebel & Ross-Hellauer [3].  The "APC effect" describes how better-resourced institutions publish in journals with higher APCs and higher perceived prestige. In fact, institutions in wealthier countries pay 42% higher average APCs, creating stratification

where less-resourced institutions are blocked from costly, prestigious publications. These charges thus have "a chilling effect on opportunity and equality for researchers from less prestigious or less wealthy institutions" [3]. While publishers offer waivers for some low- and middle-income countries, many countries like India don't qualify despite researchers facing substantial constraints. In one study, doctors and researchers reported having plenty of clinical material and the potential to write and publish, but APCs limited their options to submit academic papers [5].

The existing literature has documented the APC effect specifically leading to the exclusion of under-resourced researchers.   Our qualitative findings extend this concept. While the APC effect represents a barrier for many researchers globally, we posit a complementary "APC Trap" that binds privileged researchers who feel compelled to participate in OA publishing while simultaneously feeling powerless to change its inequities. We investigate this “APC Trap” by using our survey results, which include direct comments from well-resourced researchers about their experiences and perspectives with APCs. The researchers surveyed in our study report simultaneous price insensitivity and price outrage toward OA models, which results in a bind for those who feel obligated to use APC models despite strong feelings about their unjust impacts. This complex thinking is characterized by the "bind" of the APC Trap—a contradictory situation where APC "barriers" combine with professional anxieties and equity concerns. This results in a feeling of inability to change the status quo, even among the world's most privileged and well-resourced researchers. This article explores how the APC Trap manifests in qualitative findings, its disciplinary and institutional variations, and how it represents a difficult bind without easy solutions. We present this evidence of the ethical compromise of APCs for well-resourced researchers to demonstrate that the entrenched APC model is not working for *any author,* even those who have the easiest path to pay and the greatest benefit.

# Materials and methods

This study is drawn from a dataset collected in 2022 about publications from four United States (U.S.) research intensive universities (Carnegie Classification of R1). For full details about the dataset, see our first paper [10]. The four universities are: the University of Colorado Boulder (UCB), the University of Massachusetts Amherst (UMass), the University of Pittsburgh (Pitt), and the University of Tennessee Knoxville (UTK). These institutions are all public universities and are geographically situated in different areas of the U.S. They have similar academic profiles with degree and research representation across disciplines with the exception to hospital-based medical research. The University of Pittsburgh has a large medical school that is included in this study. The other institutions in this study exclude medical school researchers from the data because those medical schools report through different campuses with different administrative and budgetary structures.The study protocol underwent a multi-site IRB approval, with the University of Colorado Boulder serving as the primary IRB review institution. Co-Investigators at the other institutions also submitted protocols for supplemental IRB approval, and the one Co-Investigator not affiliated with an institution of higher education submitted an Individual Investigator Agreement. The study was waived as “Exempt” by all four institutions. Data were collected via a Qualtrics questionnaire, and the recruitment period was from October 31, 2023 and November 21, 2023. Written informed consent was obtained by having respondents read and accept an informed consent statement prior to completing the survey.

After the data were collected, the dataset was organized by institution and grouped into OA types: Gold OA and Hybrid OA. These categories were based on reporting categories in Dimensions, a proprietary, comprehensive scientific research database [11]. For further details, see the Methodology in our previous paper [10]. Each of these categories was then divided into three broad academic areas: Humanities and Social Sciences (HSS), Health and Medicine (HM), and Natural Sciences and Engineering (NSE). These three disciplinary categories used

for this study are based on the Fields of Research Codes included in the Dimensions dataset [11].

Six different surveys (two within each HSS, HM, NSE) were created using the Qualtrics platform, and these surveys were customized based on OA type (Gold OA or Hybrid OA). All surveys included the same two free-response questions analyzed for this paper. Survey-based analysis with open-ended questioning is well-suited for researching scholarly publishing practices and perceptions as an effective method for capturing nuanced attitudes and decision-making processes [9]. Manual coding of free text responses allows for theme development that incorporates complex social and institutional factors which can influence publishing behaviors [6,9]. These methods align with the field's focus on understanding motivations, barriers, and values; and provide contextual insights to inform future scholarly communication policy and practice. This study used a purposive sampling method, meaning that participants were selected based on specific criteria rather than chosen at random. This allowed the study to focus on individuals who were most relevant to the research questions--faculty authors engaged in OA publishing who were likely to have encountered an article processing charge. This approach helped ensure that the data collected would be useful and applicable to the goals of the research, which was originally to identify the ways that authors at R1 institutions pay for APCs. This purposive sampling allowed for a targeted analysis of the sentiments and funding sources of well-resourced researchers within the U.S. As the biggest users and drivers of the APC model of open access publishing, the sentiments of this subset of researchers may serve as a bellwether for the overall sustainability of the model globally.

While the broader study collected both quantitative and qualitative data related to faculty publishing patterns, this paper focuses on the qualitative responses to two open-ended, free-text questions included at the end of each survey. All respondents received the same open-ended questions, regardless of whether the survey was for a Hybrid OA or Gold OA journal.

These questions were designed to provide space for respondents to offer additional context, express concerns, or raise issues that may not have been fully addressed in the preceding survey questions. While there were 321 completed surveys, 154 of those also included usable responses to at least one of the open-ended questions at the end of the survey.

The first open-ended question asked, “Do you have any additional comments regarding authors’ ability to pay publishing fees for Open Access?” This question aimed to elicit personal perspectives, institutional challenges, or equity concerns related to APCs and the economics of OA publishing. The second question invited broader reflection and asked, “Do you have any additional comments about the topics in this survey?” This prompt allowed participants to provide feedback on the survey content, share general insights about OA publishing, or raise related issues not explicitly covered by the survey. However, there were not distinct or observable differences in how the 154 respondents answered across the two open-ended questions, so for the purpose of analysis the responses were aggregated into a single qualitative response, resulting in 154 responses total.

The response data was initially analyzed using automated sentiment analysis tools; the results of this analysis are briefly reported below in the *Sentiment analysis* section. After reviewing the results, we unanimously agreed that manual coding was the most effective method for analyzing the data, given the nuanced context of the language in the free text responses. All open-ended responses from both free-text questions across every respondent type and respondent group were consolidated into a single spreadsheet. The dataset was then prepared for analysis with each respondent assigned a unique identifier: a number and a short institution code.

The response data was initially reviewed to identify recurring patterns, concepts and sentiments to uncover underlying themes that reflect respondents' experiences, perceptions, or attitudes.

Through discussion, we normalized frequently mentioned themes into 8 codes. Responses identified as representative of specific themes were included as examples in the Code Book to guide the subsequent coding of survey responses. We also coded for an additional theme, called *Presupposition of Cost*, but determined it should not be analyzed as related to the APC Trap. The codes are discussed in the Results section. Respondents also conveyed broader sentiments, including frustration and appreciation, which, though not directly tied to the APC Trap, offer valuable context for understanding the overall conflicted discourse around APCs.

Following the development of eight APC Trap codes, each researcher independently reviewed participant responses. For each code, a "yes" or "no" was assigned, indicating its applicability to a given response. It's important to note that codes were applied to the full set of responses from a single respondent, rather than to individual statements, and encompassed data from both free-text questions. Therefore, each response could be assigned multiple codes which reflected themes demonstrated across the entirety of the response.

An iterative refinement process was used to ensure coding consistency and accuracy. After an initial coding review, the Code Book was refined to address any ambiguities we identified. Subsequently, each researcher coded the data independently. To measure agreement, an Excel-based formula was developed to calculate consensus on "yes" and "no" assignments. A code was definitively applied to a respondent if four or more researchers reached agreement. In instances of disagreement, all researchers discussed each specific case to reach a consensus, leading to further refinements of the codebook. This iterative process of independent coding, discussion of discrepancies, and codebook refinement continued until complete agreement on all applied codes was achieved.

# Results

Thematic analysis resulted in the identification of eight major APC Trap codes. Many more themes were initially identified; however, some were not indicative of the APC Trap, and others were combined or consolidated into a single code. For example, concerns about the future of graduate students and their inability to pay, public and taxpayer concerns, and concerns for researchers with less or no grant funding are all included in the code for *Diversity or Equity*. Additionally, there are certainly additional ways respondents indicated a feeling of being “trapped” by the APC system, which are not included here as they may not have been expressed by enough respondents to emerge in the qualitative results.

Below, we describe the eight main APC Trap codes we identified, the number of times they occurred in our dataset, along with illustrative quotes drawn from our respondents. These results provide a code book that may be of use for future research and development of this model.

## Code: Financial concern

This code was the most frequently applied in our dataset (n=52; 34%). This code focused on specific and personal financial hardships or worries on the part of the respondent with regard to paying the APC.

> *More journals are forcing open access fees if you publish with them. I publish often so it does stress me out where I'll always come up with this money from.* (#249, UTK)

*I have ample grant and internal support; payment for me is feasible, albeit it is expensive and a drain on resources. I have colleagues with far less resources which precludes their ability to pay for open-access publishing.* (#32, Pitt)

## Code: Diversity or equity

This code was used frequently in our dataset (n=50; 30%). This code was applied to responses that included comments that APCs are hurting the ability to meet diversity and/or equity goals. Equity may refer to any number of imbalances between different parties, including references to "pay-to-play" scenarios and acknowledgement that some have deeper pockets than others. Common themes in this code included the perception that those who can’t pay for open access would suffer from lower exposure and reader access to their research, as well as increased bias towards the subjects of interest for wealthier institutions.

*I just think it is inherently unfair that the public doesn't have open access to ALL published materials, especially when the opportunity to pay for publishing is not equally distributed among faculty. We need a better system! As you can see by my answers above, I object to the fees on principle, not because of the cost. Why should I benefit from paying a fee that others' can't afford? Why would I want this to be a reason why my work is used/cited by others? It should be read and used/cited on its merits, not some artifact of gamesmanship or privilege.* (#25, Pitt)

*It's a diversity and equity issue. People from historically marginalized groups are least likely to be able to pay publication fees. Plus, publishing companies make LOTS OF MONEY and do not need these fees to be this high.* (#158, UCB)

## Code: Threshold of reasonableness

This code was used 46 times (28%) in our dataset, most commonly when respondents indicated that there was an upper limit of their tolerance for APCs. This theme is broader than *Financial Concern* (which focuses on specific, personal concerns for affording a specific fee) and can include more general statements about the state of the field and questioning the reasonableness of the current system of APCs in OA publishing. This code also applies to responses that call out the exploitative or excessive practices of publishers, implying that there are “reasonable” limits for fees and profit.

> *I recognize that there is labor involved in the publishing industry, but the costs to publish are out of control. Especially when you consider the profit margins enjoyed by these publishers.* (#272, UMass)

> *I want to emphasize that help from the University and the departments is greatly appreciated. The cost of publishing has gone up significantly in the last 5 years that it is very difficult to find that kind of money. The amount needed to publish is very unreasonable and I do not understand why the cost has increased. It is very frustrating to investigators like myself, who want to publish in high impact journals, but, cannot always do so.* (#227, UTK)

## Code: Restriction of decisions

This code was used 40 times (24%) in our dataset. This code indicates that respondents made choices they might not otherwise have made because of an APC – specifically, that they had to make a choice for themselves, not that unnamed other parties have their decisions restricted. Restrictions included mandates or grant requirements that restrict a possible set of publication

outlets, or choosing different journals due to an APC that made a particular outlet unaffordable. This code often indicates a sense of powerlessness.

> *I have a well-funded lab and access to a decent amount of discretionary funding to pay for publishing fees, but then I often struggle because the highest-impact / most valued journals have the highest publishing fees, meaning that sometimes I have to choose between a high-valued venue and a venue I can afford.* (#170, UCB)

> *I felt a bit trapped into the high fees ($3090) for this journal. We had originally submitted to a different (higher impact Nature family journal, which I think could have been worth the cost for its scope and recognition. But, after review, the editorial staff suggested we transfer to a more specialized Nature family journal. At that point, it would have been too time consuming and cumbersome to pull the paper and resubmit to a completely different journal, so we went ahead. But the fees for this journal do NOT match its standing or reputation, and I could have easily submitted this article to a no cost PLoS journal if I had realized earlier how much this journal would cost.* (#194, UTK)

## Code: Grants don’t cut it

This code was applied to responses (n=35; 21%) that specifically indicate limitations in grant funding, whether grant funds were used to pay for the APC or not. This code often referred to grant funds not being enough for all that the respondent needed to do for their research or academic expectations. This code was not applied to acknowledgements that some others don't have grant funds to cover these costs (which would be coded Diversity/Equity). One theme that recurred in this code surrounded the logistics of paying APCs with grants and the various hurdles and barriers that caused for publications.

*Ultimately seems like a good model, but in the near term we are increasingly shifting the burden of publication costs from less-publishing consumers (industry, non research-intensive academic) to grants, without most funders increasing grants to keep up (just one of many costs of research growing faster than grants) which makes things challenging. As a result I try to avoid paying open access fees whenever possible.* (#300, UCB)

*People with NIH [National Institutes of Health] and NSF [National Science Foundation] money get much more and are required to publish open access. USDA is not that case and all of my funding comes from that, leaving me to use valuable discretionary funding that could be used for other important purposes (equipment, equipment maintenance, etc.)* (#274, UMass)

*There is a Catch-22 when it comes to using grant funding to cover the APC. Typically we are submitting manuscripts for publication at least a year after our research funding expires. Therefore we can't pay the APC from grant funds unless the NIH allows us to retain that money after we close out the grant.* (#95, Pitt)

## Code: Academic obligation/expectation

Responses coded for *Academic Obligation/Expectation* (n=33; 20%) indicate a strong expectation or obligation to publish OA for professional advancement. These respondents mention pressures surrounding promotion, publication impact, student benefits, or to conform with disciplinary norms. This code is similar to Restriction of Choice but often without a specific decision point, and often indicates a feeling of being trapped by expectations around the job.

*IF journals require publishing fees, researchers should be allotted money for this since it is a requirement of my job.* (#156, UCB)

*APCs should be supported by Dept./College/University because publishing is an expectation/requirement of their job and benefits the institution's reputation.* (#221, UTK)

## Code: Academic labor

The *Academic Labor* code (n=28; 17%) references responses about academic work (including peer review) that is often done for free and without benefit, while a journal or publisher profits from APCs. These responses often include references to merit with journals (such as a waived fee as a reward for labor) and volunteerism required by some journals.

*I agree with open access publishing, but I do not understand why the onus is on investigators to pay instead of publishers. Journals receive free peer review, pay next to nothing for editorial board stipends, receive ad revenue, and now have small publishing costs as print journals are dwindling. It would be reasonable if investigators covered only costs related to editorial stipends, copy editing, and online hosting (assuming that publishers will provide these data transparently).* (#40, Pitt)

*The publishing business is very difficult to understand. We, researchers, write and review the papers that are published for free. The publishing houses control the logistics and nothing else, for which we pay them. Then, the authors receive no royalties and the publishers get all the profits; that seems unfair.* (#152, UCB)

*We provide peer-review service, editorial service.. Journals APCs are legalized thevery [sic], esp. at high-end range.* (#327, UTK)

## Code: Quality or rigor

Several (n=20; 12%) respondents mentioned concerns that OA reduces the quality or integrity of the research. This code often co-occurs with concerns about perverse incentives for publishers to accept papers of dubious quality from authors who can pay the fee, or with speculation that high Impact Factor journals or those that are perceived as elite are exploiting their perceived high-quality status to charge higher APCs.

> *I don't like the model where researchers pay a lot (in some journals) to publish their work - it creates bad incentives to publish bad research in some cases, and makes it so people with less financial backing cannot have their papers openly accessible in others.* (#164, UCB)

> *Page charges and open access fees are being exploited by so-called elite journals because of their perceived status. Individuals are more than willing to pay $5-10K or more to publish in Nature and Cell title journals often because of careerism. Members of promotion and tenure committees or grant review panels are often so specialized that they cannot evaluate science outside of their narrow areas. So having the imprimatur of Cell or Nature in the journal title gives the allure of significance or importance. This is corrupting.* (#6, Pitt)

## Presupposition of cost

As part of the analysis, responses were also coded for *Presupposition of Cost*, meaning that the respondent conflated OA with an article processing charge or other fee. Several of our respondents positioned payment for OA publication as an expected fact about the world, although about 71% of the journals in the Directory of Open Access Journals do not charge article processing fees as part of their business model [12]. This is a linguistic phenomenon

known as *presupposition:* speakers assume that readers know some information and reference that information when discussing other information. This presupposition that OA requires a publication fee has been noted in other research [13]. However, in this work, this code was not analyzed as part of the APC Trap for two reasons: first, because this section of the survey was primarily answered by authors who had paid an APC for their publication (and, therefore, the existence of the cost was in fact true), and second, the wording of the question contained a presupposition of cost in itself (“Do you have any additional comments regarding authors' ability to pay publishing fees for open access?”).

It remains essential to highlight this presupposition, as it reveals a trend in conversations and marketing surrounding OA. Conflating OA (a model for the availability and use of research articles) with article processing charges (a business model for publishing) can not only entrench beliefs about publishing options but also complicate or obfuscate the purpose of publishing OA [13]. The conflation of OA with APCs can make the APC Trap feel more like an Open Access Trap, erasing the potential of Diamond OA (that is, publishing without fees to the author or reader) and other models, tarnishing the practice of open research by equating it to complex market and privilege dynamics illustrated by the other components of the APC Trap.

Below are some examples of responses that contain a *Presupposition of Cost* for illustrative purposes.

> *This discussion is useless. There is a cost to publish a paper, and it does not matter what people feel they would like to pay for a paper. Somebody always paid, and with subscription journals, libraries pay for it - i.e., us.* (#198, UTK)

This example from respondent 198 contains a clear presupposition using the construction “There is”: “There is a cost to publish a paper”. This presentation of a fact presupposes that this is a truthful state of the world. Linking the subscription cost to publication costs is a type of presupposition often used in publisher marketing – to suggest that if they are not generating revenue by charging a fee for a subscription, then they must charge a different fee. This response was not coded for any other codes associated with the APC Trap, indicating that this presupposition is not always linked to the APC Trap.

*Presupposition of Cost* can co-occur with other APC Trap codes. In this response from #181, UTK, there are two other aspects of the APC Trap: *Grants Don’t Cut It* and *Academic Obligation/Expectation*:

> *[O]pen access is mandatory now in projects that are federally funded. The open access model drains the grant funds because my last paper cost more than 3k. The Open Access fund would only pay 1800 of this and there is no guarantee of this funding.* (#181, UTK)

This example contains the presupposition “[t]he open access model drains the grant funds.” While it is not clear if they are referring only to one example of an APC associated with  the publication of an article draining grant funds, the reference to the “open access model” presupposes that the model itself, and not, for example, a certain instance of that model, is to blame.

Presuppositions can also occur when expressing nuance around expectations for costs; these instances sometimes co-occur with the *Threshold of Reasonableness* APC Trap code:

*It is acceptable for open access journals to require publishing fees since they cannot obtain revenue from subscriptions or advertisements. However, those that only publish online should have lower fees (<$750) compared to those that publish online AND in print (<$1500).* (#60, Pitt)

This example contains a presupposition of revenue associated with journals, whether through subscriptions, advertisements, or publishing fees. In this case, the *Threshold of Reasonableness* code applied to this response means that while the respondent might accept the existence of a publishing fee, they still exhibit a sense of the APC Trap by expressing a preference for lower fees than the reported average.

# Discussion

Out of 321 total responses to the survey, 154 had a usable response to at least one of the two open-ended questions: “Do you have any additional comments regarding authors' ability to pay publishing fees for open access?”, and “Do you have any additional comments about the topics in this survey?” By “usable,” we mean that the text field contained substantive content beyond responses such as “No comment” or “N/A.”

After reaching a threshold of agreement for codes on all responses, we were able to analyze the results to surface themes across the APC Trap elements. In this section, we first analyze the coding of the qualitative responses through quantitative analysis, then demonstrate the layered nature of the APC Trap by analyzing these codes alongside the other survey questions as well as alongside other codes.

Our thematic analysis of qualitative data identified several core motifs around which authors were fixated in relation to APCs, and which worked together to reveal a contradictory but

complementary sense of obligation and exasperation over paying for OA publishing. It is this bind - this acute cognitive dissonance - that we coined as the APC Trap.

## APC Trap elements

Across all respondents to the survey who provided a usable open-ended response (n=154), 90% of those respondents' answers demonstrated at least one APC Trap theme (n=139), indicating that the APC Trap is a phenomenon experienced by R1 authors in a wide array of contexts. Fig 1 shows the totals for the number of APC Trap elements coded within a single response.

**Fig 1. Responses by number of APC Trap elements.**

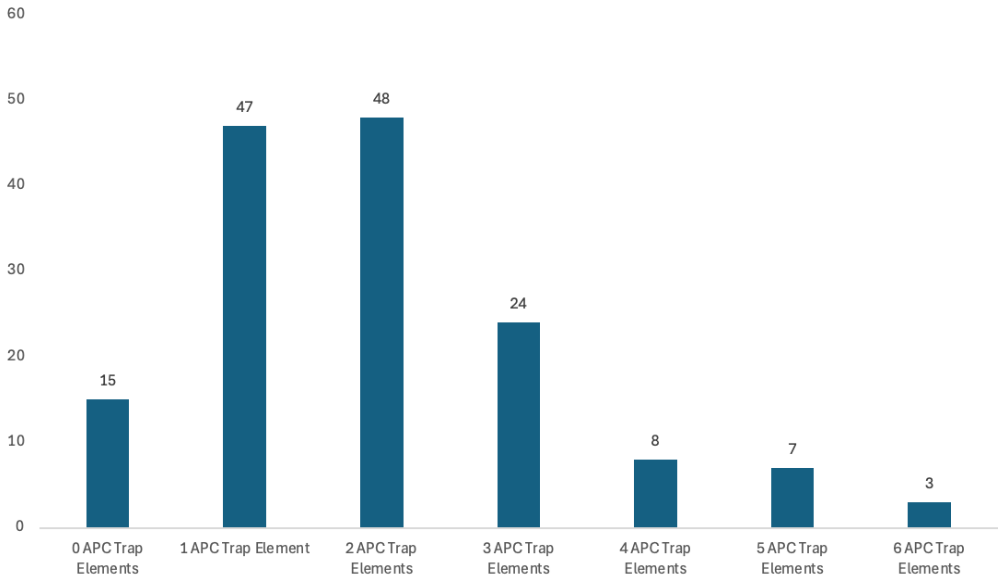


Most commonly, responses contained one or two APC Trap elements, with 62% of responses (n=95) falling into these categories. Twenty-seven percent of responses (n=42) contained three or more APC Trap elements. There were 15 responses which did not demonstrate any of the eight elements we identified, while 3 responses demonstrated six elements within the open-ended responses. No responses demonstrated seven or more APC Trap elements.

Example response with no APC Trap Elements:

*"I like the idea of a University fund to support publication costs."* #94, Pitt

Example response with six APC Trap Elements:

*"I really think these fees have gotten out of hand. Especially for junior researchers who may have little or no extramural funding, the fees present a dilemma. You have to publish to advance, but if you run out of money you won't be able to continue your research, and you won't advance. For the particular journal article that this survey referenced, the publisher offered to waive the fee if we submitted our work to that particular journal. This was the determining factor for me. If I hadn't had to choose between one journal that didn't charge a fee and a handful of others that would charge $3,000+ (!), I probably would have submitted to one of the other journals. But since I really felt like I needed to conserve resources, I opted for the journal that would waive the fee. Not a great system. I also really don't understand where these fees go/what they are funding. They seem exorbitant for, what exactly? Putting my document into a template, loading it on a website, and adding it to some databases?"* #36, Pitt

Table 1 shows the total number each element was coded as present in a response, as well as the percentage of responses out of 154 for each element.

**Table 1. Totals for each APC Trap element, and percentage of total responses.**

| **APC Trap Element** | **Total** | **Percent of Total** |
|---|---|---|
| Financial Concern | 52 | 34% |
| Diversity or Equity | 50 | 32% |
| Threshold of Reasonableness | 46 | 30% |
| Restriction of Decisions | 40 | 26% |
| Grants Don't Cut It | 35 | 23% |

| | | |
|---|---|---|
| Academic Obligation/Expectation | 33 | 21% |
| Academic Labor | 28 | 18% |
| Quality or Rigor | 20 | 13% |

*Financial Concern* was the most common theme coded, with slightly more than a third (34%) of respondents expressing personal financial hardship. This may be due in part to respondents interpreting the first open-ended question about “authors’ ability to pay publishing fees” as a question about their own ability to pay. About a third (32%) of responses also included *Diversity or Equity* concerns, demonstrating that the inequity of APC-based OA models is top of mind for many researchers.

## Demographics

Demographic factors about respondents were gathered prior to the survey and were not based on respondent self-selection. There were some distinctive demographic variations in both the extent to which the APC Trap was experienced and the types of concerns which were most prominent.

Tables 2-4 display the average number of APC Trap elements coded based on these demographic factors, rounded to two decimal places.

**Table 2. Average APC Trap elements by discipline.**

| Discipline | Average APC Trap Elements |
|---|---|
| Humanities and Social Sciences | 2.21 |
| Natural Sciences and Engineering | 1.95 |
| Health and Medicine | 1.95 |

**Table 3. Average APC Trap elements by institutional affiliation.**

| Institution | Average APC Trap Elements |
|---|---|
| University of Massachusetts | 2.59 |
| University of Colorado Boulder | 2.06 |
| University of Pittsburgh | 1.97 |
| University of Tennessee Knoxville | 1.59 |

**Table 4. Average APC Trap elements by OA type.**

| Open Access (OA) Type | Average APC Trap Elements |
|---|---|
| Hybrid | 2.21 |
| Gold | 1.94 |

HSS demonstrated a 12.5% higher rate of APC Trap elements, on average, than the other two disciplinary categories. By institution, UMass responses demonstrated by far the largest number of APC Trap elements on average. Responses from authors of Hybrid articles demonstrated slightly higher average APC Trap elements, at 13% more APC Trap elements, on average, than responses from authors of Gold articles.

Fig 2 shows a disciplinary breakdown of responses that include APC Trap elements. Respondents in HSS disciplines, as well as respondents of Hybrid OA articles, experienced the APC Trap more acutely than their peers. While we cannot make broad-stroke generalisations since each respondent is unique in their experience of the APC Trap, it is likely these groups experience more financial pressure and fewer alternative funding options than their peers in this study. HSS authors are less likely to have available grant funding. According to the OpenAPC dataset hybrid articles have significantly higher average APCs, placing additional financial pressure on authors [14]. This suggests that lack of institutional funding and broad OA initiatives impacted respondents who felt more financial strain or a restriction of decisions when paying these costs.

**Fig 2. Percent of APC Trap elements by discipline.**

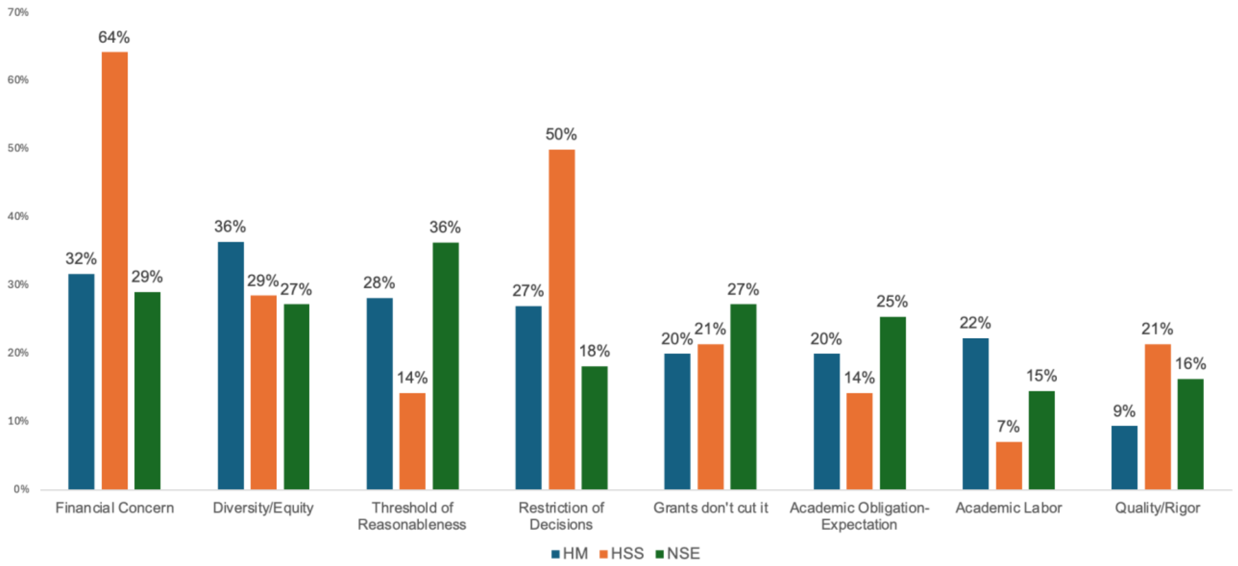


HSS responses were twice as likely as HM and more than twice as likely as NSE responses to display *Financial Concern*. Similarly, HSS responses were almost twice as likely as HM and more than twice as likely as NSE responses to display a *Restriction of Decisions. Academic Labor* was by far the least likely element to appear in HSS responses.

For NSE respondents, *Threshold of Reasonableness* was the most significant APC Trap element, with over one third (36%) of HM responses demonstrating this code, followed by *Financial Concern*, *Diversity or Equity* and *Grants Don't Cut It*. *Academic Labor* was the least coded element in NSE responses.

*Diversity or Equity* was the most common APC Trap element for HM respondents, with more than a third (36%) of responses demonstrating this code, followed by *Financial Concern* and *Threshold of Reasonableness*. *Quality or Rigor* was the lowest concern demonstrated by HM responses. *Financial Concern* was either the top or second-highest concern across all disciplines.

Generally, HSS respondents were twice as likely to experience the "personal" trap elements (*Financial Concern* and *Restriction of Decisions)*, while the other disciplines were more likely to focus on the "systematic" concerns of OA publishing not being reasonable or equitable for everyone (*Threshold of Reasonableness*, *Diversity or Equity*, *Academic Labor*). This suggests that the APC Trap exists on a kind of spectrum: If an author can't pay to begin with, their concerns are focused on how to participate within the system. If an author can pay and holds a somewhat privileged status, concerns shift to broader inequities or systemic concerns in scholarly publishing.

Fig 3 shows APC Trap codes separated by institution. More than half (53%) of UMass qualitative responses expressed *Financial Concern*, while nearly half (47%) expressed a *Restriction of Decisions* around OA funding. UMass responses were also the most likely to identify *Diversity or Equity* concerns around OA publishing. In fact, out of the eight APC Trap elements analyzed for this study, UMass respondents demonstrated five of them more acutely than the other three institutions (*Financial Concern*, *Diversity or Equity*, *Restriction of Decisions*, *Grants Don't Cut It*, and *Academic Labor)*. Even as our first study demonstrated the majority of APCs being paid through grants, authors at institutions with fewer alternative funding sources (such as departments or a library fund) may experience additional and compounded strain over the payment of APCs.

**Fig 3. Percent of APC Trap elements by institution.**

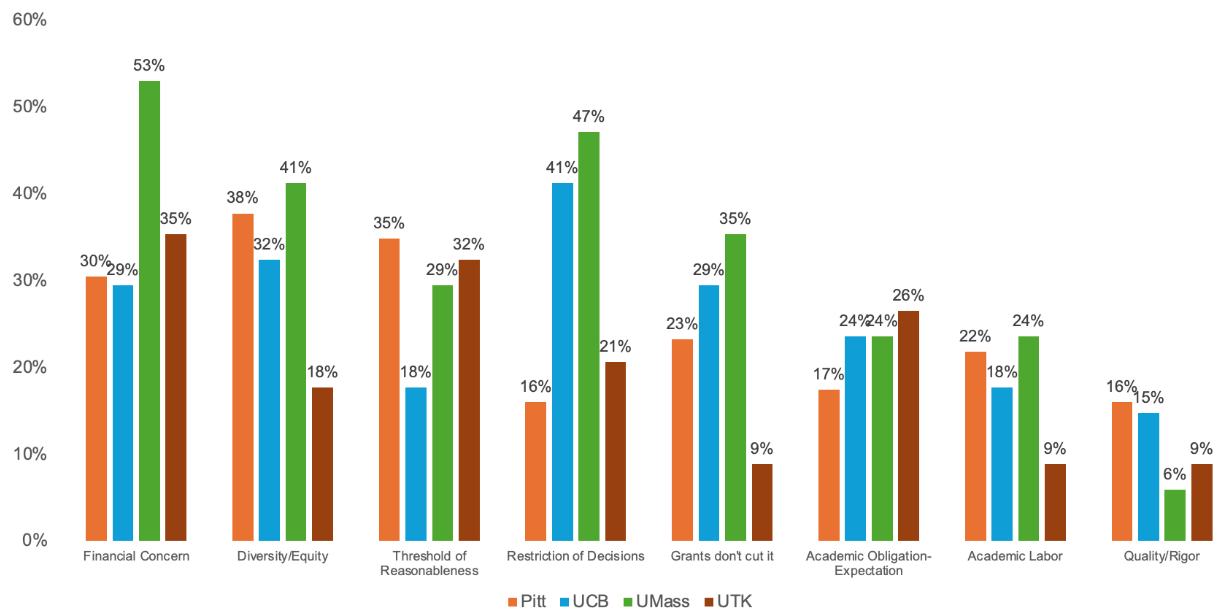


For Pitt, *Diversity or Equity* concerns (38%) was the biggest APC Trap element identified, followed closely by *Threshold of Reasonableness* (35%). Interestingly, while *Financial Concern* was the most identified APC Trap element in UTK responses, with over one-third (35%) of responses displaying *Financial Concern*, UTK also had significantly fewer responses indicating that *Grants Don't Cut It* - 9%, compared to a quarter or a third of responses at other institutions. This likely indicated that financial issues brought up by UTK responses were more often of a personal nature. For UCB responses, *Restriction of Decisions* was the most common APC Trap element identified (41%) by a fairly large margin, followed by *Diversity or Equity* concerns (32%).

Fig 4 shows APC Trap elements separated by kind of publication, Gold or Hybrid OA. Authors of Hybrid articles were almost twice as likely as Gold OA authors to indicate in their responses that *Grants Don't Cut It.* This can certainly be explained in part by the fact that a portion of Gold respondents did not pay an APC at all, but may also indicate the pressure of higher APCs under the Hybrid OA model.

**Fig 4. Percent of APC Trap elements by journal OA type.**

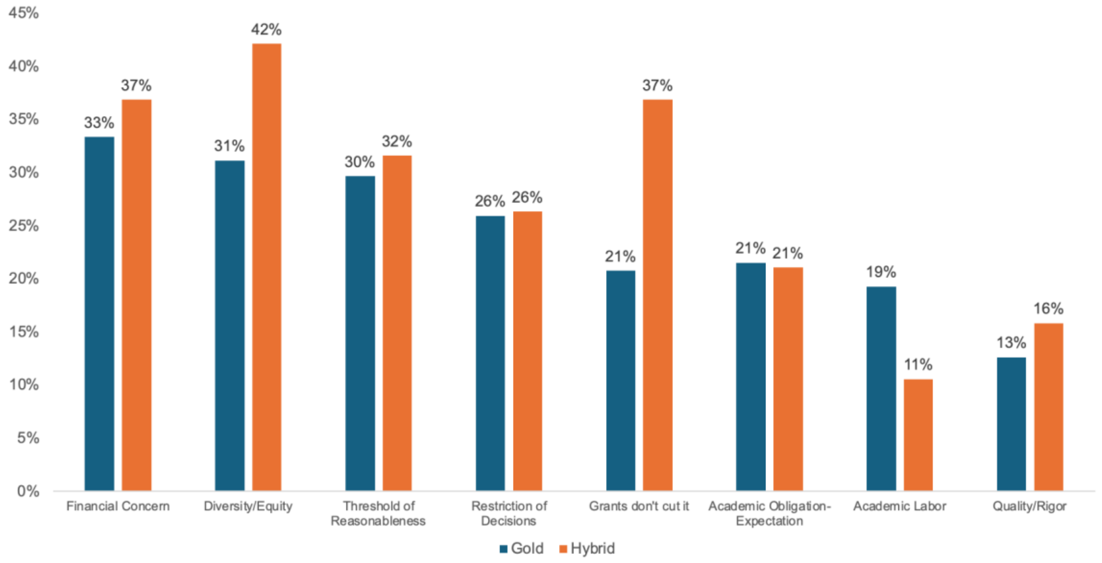


In fact, respondents who published in Hybrid OA journals demonstrated every APC Trap except *Academic Labor* more frequently than respondents who published in Gold OA journals. While the cost of Hybrid OA is likely an important factor, we cannot attribute this difference solely to costs, as respondents of Hybrid OA articles were also more likely to demonstrate concerns about the *Quality or Rigor* of OA publishing, as well as concern for *Diversity or Equity* (42%), suggesting these respondents have elevated concerns about the standards and viability for such models even as they willingly participate in them.

## Sentiment analysis

In addition to the coding of the APC Trap elements, we attempted to determine the sentiment of the qualitative responses as a next logical step in understanding the respondents' points of view and concerns about the APC Trap elements. Two different sentiment analysis tools were tested: NVivo's Auto Sentiment Coding and SentiStrength [15]. Unfortunately, both tools had significant

drawbacks that resulted in the results and analysis being unusable. These are reported here because they represent a potential limitation to this study and future avenue for investigation.

The primary drawback was that each response often flagged both tools for simultaneously positive and negative analysis. For example, NVivo categorized as “moderately positive” responses that expressed support of OA in principle with concerns about inconsistent fees and unequal burdens on underfunded authors. SentiStrength offered even more mixed results because it used a numerical scoring approach, assigning values to individual words in each response. For several responses that all authors agreed to be strongly negative, SentiStrength assigned scores of 0 or even positive values due to simplistic keyword associations with isolated words such as "profit" or "enjoy," without accounting for the context of the response.

The results from these experiments highlight a limitation of automated sentiment tools when applied to complex, nuanced topics like academic publishing models: they often fail to grasp sarcasm, rhetorical structure, or compound sentiments. These features appeared often in the analyzed dataset, since the respondents had strong emotions and the subject matter is deeply complex and intertwined with power, equity, and institutional critique. As such, a sentiment analysis is not included in this results discussion, although this may be a promising avenue for future analysis.

## The APC Trap based on responses to other survey questions

Some APC Trap codes have a clear relationship with other survey questions, which can provide some indicator of sentiment of the respondent. For example, Fig 5 shows that respondents who indicated it is more difficult for them to pay are more likely to have left a comment coded as *Financial Concern*.

**Fig 5. Percent of respondents' comments coded as *Financial Concern* viewed by how respondents perceive their own ability to pay APCs relative to their peers.**

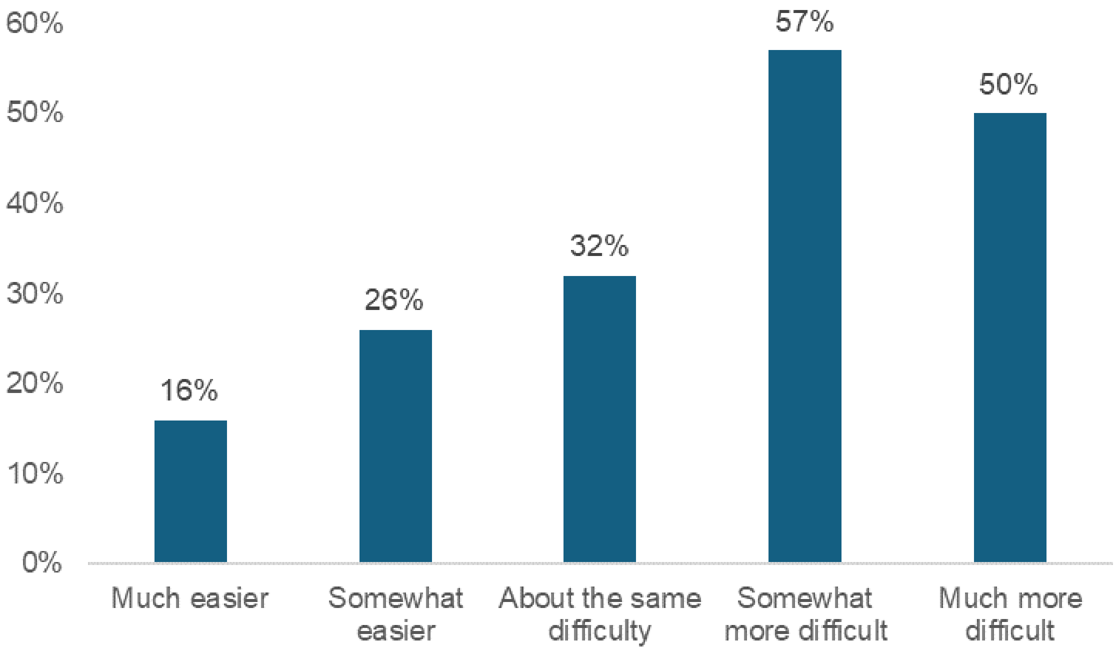


Similarly, when respondents said it was much *easier* for them to pay APCs, they also were less likely to leave a comment that was coded as *Grants Don't Cut It* (Fig 6). This might indicate that participants who have larger grants or significant institutional support already find it easier to pay for APCs in general because they are accustomed to financial support. The responses for those who indicate it is more difficult to pay APCs also report difficulties with support in general, as in the following response:

> *"The fees keep increasing as my obligations decrease the time I have to write grants to pay for these costs! Plus it stinks to spend a month of a grad student's salary on OA. Supporting trainees is where I'd prefer to spend money."* (#139,UCB)

**Fig 6. Percent of respondents' comments coded for *Grants Don't Cut It* viewed by how respondents perceive their own ability to pay APCs relative to their peers.**

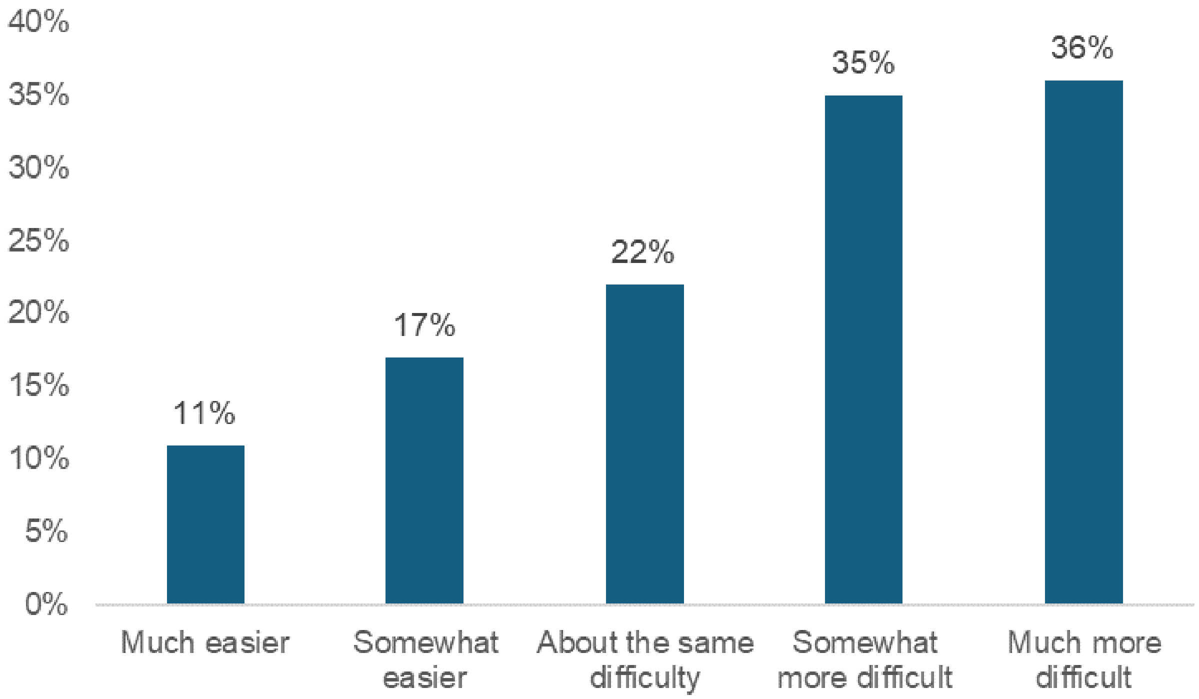


Some other interactions of survey elements reveal additional elements of the APC Trap. Part of the APC Trap is self-awareness; that is, respondents reported a moral quandary of participating in the system of paying APCs for OA even though they knew that it was inequitable and made publishing more difficult for others. This sentiment surfaced when looking at the responses of those who reported that it was easier to pay APCs than others in their field.

Specifically, the easier that respondents thought it was for them to pay fees themselves, the more their comments involved *Diversity or Equity Concerns*. Fig 7 shows the upward trend of codes for *Diversity or Equity Concerns* as the self-reported ease of paying for APCs rises. This demonstrates a correlation between awareness of ease and inequity, suggesting that perhaps at least some respondents recognized that participating in paying APCs presented a moral quandary.

**Fig 7. Percent of respondents' comments coded for *Diversity or Equity* viewed by how respondents perceive their own ability to pay APCs relative to their peers.**

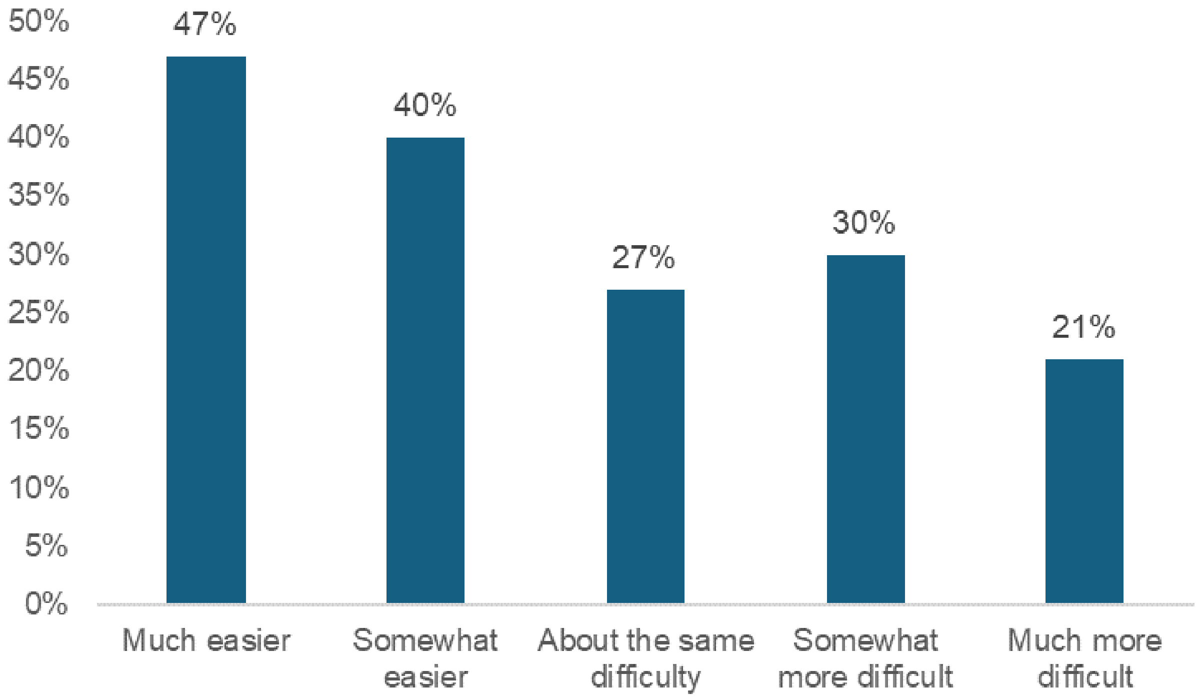


Another APC Trap moral quandary is the interaction between APCs and perceived ethics in publishing or academic conduct. When we looked at the respondents who indicated a reasonable amount to pay for APCs, we noticed an interesting trend related to this concept. Respondents who indicated any amount is reasonable (that is, those who did *not* say "no fees are reasonable"), were much less likely to demonstrate concerns about *Quality or Rigor* (Fig 8).

However, for respondents who indicated "No fees are reasonable", *Quality or Rigor* was the second-most prominent code in their responses (second only to *Diversity or Equity)*, present in 31% of their responses.

This alignment between a response of "No fees are reasonable" and a concern about *Quality or Rigor* may indicate conflation between article processing charges and a low-quality, review-

minimal “pay to publish” model, which is a sentiment often expressed in the responses themselves. The following response provides a succinct example:

> “*I think open access tho [sic] well intentioned is a disaster. Authors should not be in the position of pay to play.” Even for those who do not want to participate in the APC system and who think that fees are completely unreasonable, they indicate a recognition that those who do participate are setting up an ethics problem in publishing related to the pressure to publish low quality materials.”* (#256, UMass)

**Fig 8. Percent of respondents’ comments coded for *Quality or Rigor* viewed by the amount respondents’ perceive is reasonable to pay for APCs.**

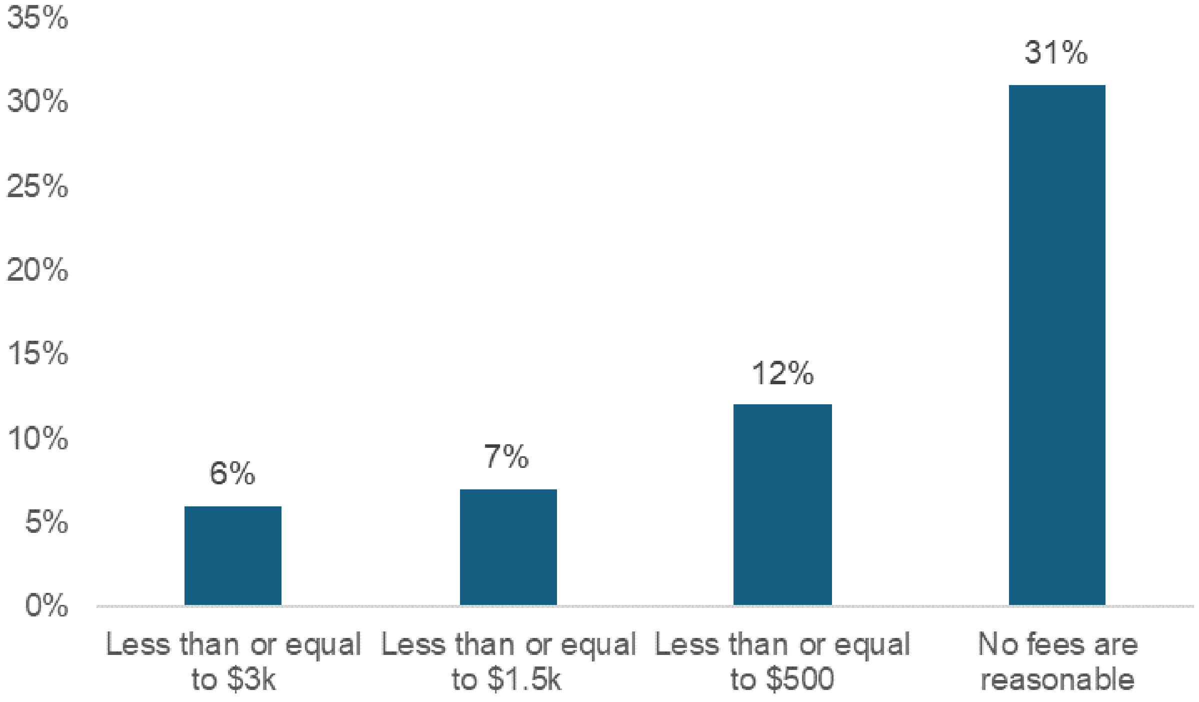


## Cross-tabulation of the APC Trap elements

In our analysis, we noted that some codes occurred together more often than others. Table 5 lists and highlights codes with a co-occurrence rate higher than 20% – in other words, out of

responses coded with either of two elements, the shaded boxes in the table indicate the percent of those which coded for both exceeded 20%.

**Table 5. Correlation matrix among APC Trap elements**

| | Financial Concerns | Restriction of Decisions | Diversity or Equity | Grants Don't Cut It | Academic Obligation / Expecta-tion | Quality or Rigor | Threshold of Reasonabl eness | Academic Labor |
|---|---|---|---|---|---|---|---|---|
| Financial Concerns | 100% | 37% | 36% | 18% | 27% | 8% | 15% | 8% |
| Restriction of Decisions | | 100% | 13% | 25% | 24% | 7% | 16% | 10% |
| Diversity or Equity | | | 100% | 13% | 14% | 21% | 10% | 11% |
| Grants Don't Cut It | | | | 100% | 21% | 6% | 11% | 3% |
| Academic Obligation / Expectation | | | | | 100% | 8% | 18% | 7% |
| Quality or Rigor | | | | | | 100% | 12% | 9% |
| Threshold of Reasonabl eness | | | | | | | 100% | 21% |
| Academic Labor | | | | | | | | 100% |

*Financial Concern* and *Restriction of Decisions* were the most likely to co-occur within a given response, with a 37% co-occurrence rate. This is not surprising considering these are the two most "personal" codes, with responses most likely to provide personal anecdotes about difficulties with publishing fees. Here are a couple of examples of this cross-section of *Financial Concern* with *Restriction of Decisions*:

> *"I had to cut my salary in order to pay the increased fee for "mandatory" open access"* (#133, UCB)

*“I wrote an Opinion Piece, which cost about $400. The price determined what I wrote. I could not have afforded to submit an article.”* (#163, UCB)

Interestingly, *Financial Concern* and *Diversity or Equity* responses co-occurred at a similar rate (36%). This is likely due to the fact that our codebook indicates that *Financial Concern* responses may involve the respondent identifying as a class or category of people with financial strain, while the codebook’s instructions for *Diversity or Equity* are quite expansive and include references to any number of imbalances between different parties or stakeholders. Both of these codes, in other words, deal with delineation between the self and others, of *me* and *them* (typically more or less privileged researchers). Respondents with this co-occurrence were adept at understanding the wide variation of ability and need in funding OA publications. The following responses are examples of this unique crosstabulation:

“*The proportion of grant money that has to be set aside for APCs feels ridiculous, and unfair when that funding comes from public tax dollars and then is just shuttled straight to wildly profitable publishers. Also, publications in "good journals" are required to get funding in the first place, and this can place a strain on researchers who don't yet have funding, but need to publish.”* (#232, UTK)

*"Open access fees really just fuel inequities in publications AND contribute to a lack of diversity in fully promoted and tenured professors at universities nationally. Those who have money can pay, be published, and get more citations, and those who don't...well don't. And then they are forced to move on to a different institution or career path. It's a huge problem from a DEI perspective in academia."* (#120, Pitt)

*Financial Concern* and *Academic Obligation/Expectation* also had a high co-occurrence rate (27%). Responses invoke both personal financial strain as well as a sense of professional expectation or pressure to publish in particular journals, whether for the sake of the respondent's academic tenure, promotion or job retention, or for the professional recognition of students who hope to become full-fledged researchers in the future. This co-occurrence is a great demonstration of the APC Trap at work, whereby a sense of obligation is strong enough to override conflicting and often unsustainable financial strain. This is exemplified in the following response:

> *"I would estimate that I'm paying $10-15k per year in publication fees because so many journals in my field have switched to open access and I want to make sure students are publishing in places that keep them competitive for future research careers. UMass right now will partially subsidize one of those publications (but I need help with 4-5 pubs per year). It is completely unsustainable and I have no idea how I will continue to pay for publications."* (#257, UMass)

In the last two high co-occurring pairs, *Restriction of Decisions* is a recurring theme. The combination of *Restriction of Decisions* and *Grants Don't Cut It*, which co-occur in 25% of responses, points to the strain multiple grant recipients expressed in their responses – even with grant funding, publishing decisions may feel forced, unsustainable, or, in the words of this researcher below, "absurd."

> *"Fees of $6000 to $10000 to publish after slow reviews and turnaround times by the journal are absurd. The system is broken yet we are often required or encouraged to publish in open access journals by NIH et al but we do not have the funds in our limited budget to cover this. The fees are not sustainable with the current level of support by*

> *NIH and non-profits. These fees may eat up as much as 10-20% of a small non profit grant. How are we supposed to cover our salaries, our lab salaries, pay for space and supplieds [sic], and then also pay these absurd fees?”* (#5, Pitt)

Taking up one last example, *Restriction of Decisions* and *Academic Obligation/Expectation* – which co-occur in 24% of responses – overlap in the codebook in the sense that academic obligations often create real or perceived restrictions in publication decisions by authors. When there is an expectation within the field of study or the institution to publish OA or within certain high-impact journals, some of which charge APCs, this creates layered tension and frustration. Authors might feel obligated to pay fees to advance their careers, even if they might not agree on moral grounds or if it limits their decision-making. The following response demonstrates this quandary:

> *Ability to pay fluctuates greatly depending on grant funding. But so long as the "prestige" associated with these journals is a key factor in evaluating faculty members, its [sic] hard not to [publish] there.* (#82, Pitt)

## The messy knot of the APC Trap

Our analysis and the examples provided show that the APC Trap is not a simple or straightforward phenomenon. The extent of its impact and the level at which any one researcher might experience any number of APC Trap elements is highly dependent on an individual’s positionality. This includes their positionality within their discipline, within their institution, as well as their perceived positionality within a global and national context. There are likely many other points of positionality which could influence a researcher’s experience of the APC Trap, such as being tenured or non-tenured, which were not variables in this study.

In other words, one researcher’s experience of the APC Trap may look entirely different from another, depending on their positionality and their unique constraints and privileges. The key, unifying feature of the APC Trap is its resulting dissonance or “bind” – a frustration and acute sense of injustices within a system coupled with a need, obligation, or sense of moral responsibility to participate in such a system. This “bind” creates a sense of powerlessness, which only serves to pull the knot tighter.

# Limitations

The most prominent limitation of this study is that the survey instrument was not intentionally designed to facilitate a robust qualitative analysis. The initial aim of the study was to identify funding sources for APCs and perception of authors’ ability to pay related to peers and to authors at less advantaged institutions, and the open-ended questions at the end of the survey were intended to capture additional thoughts regarding authors’ ability to pay these fees to supplement the quantitative analysis. However, once the survey was completed, it was clear that many of the 48% of respondents who provided usable open-ended responses were expressing frustrations and anxiety about their experiences with OA publishing which went far beyond the original scope of the study. In other words, the survey was not designed from the outset to measure the APC Trap. Future research might find ways of better targeting and measuring the experiences which we observed obliquely.

Additionally, while many respondents used the open-ended questions to suit their own purposes, the framing of the questions themselves undoubtedly led many survey participants to respond in a particular manner. The two open-ended questions asked were:

> *Do you have any additional comments regarding authors' ability to pay publishing fees for open access?*
>
> and

*Do you have any additional comments about the topics in this survey?*

The first question asks respondents specifically to speak to financial concerns related to APCs, and the second question also favors commentary about payment and cost because those were the topics of the previous multiple-choice questions in the survey. Questions targeting other aspects of OA publishing likely would have revealed additional APC Trap elements not captured by this study, and may have impacted the degree to which financial concern and other themes related to finances (*Grants Don't Cut It*, *Threshold of Reasonableness*) were observed in the analysis. As noted previously, this concern about leading questions is also the reason we opted to exclude *Presupposition of Cost* from the final analysis.

Lastly, this study was designed to capture the sentiments of authors at very research-intensive (R1) institutions based in the U.S., and so its implications for smaller and less well-resourced institutions, as well as global contexts, are limited. However, some limited, broader implications of the study are detailed in the following section.

# Conclusion and Broader Implications

Since APCs started gaining traction as a financial model for OA publishing at the beginning of the 20th century, scholars, librarians, authors, and some publishers have raised serious concerns about the long-term sustainability of the model. There have also been concerns about how APCs re-entrench uneven and inequitable participation in knowledge sharing and distribution.

This study confirms these concerns in the U.S. but may have global implications which could be explored in future studies. These concerns manifest in the privileged researchers of this study in the form of a phenomenon we coin the APC Trap and are characterized by eight overlapping and interacting characteristics. The eight elements of the APC Trap identified in this study mean

that its actualization is unique from one researcher to the next. The limited scope of this study to four R1 institutions in the U.S. also means that there are undoubtedly valences of the APC Trap which are not captured in this study. However, our findings demonstrate how this phenomenon surfaces in the lived experiences of this niche set of researchers.

There are a couple of broader implications to be drawn from this study. First, whether the APC Trap is observed in wider, global contexts or not, its pervasiveness even within this relatively small sample suggests the APC funding model faces many challenges to adoption and acceptance in the U.S. even when a strong ethical and ideological drive to publish open access is present. Second, if privileged researchers from some of the most well-resourced universities in the United States have such an agonized viewpoint of APCs, it accentuates the pain and hardship this model presents in other regions and socioeconomic contexts.

It is true that APCs are not the only model of sustainability for open access publishing, and a variety of alternative models have sought to repair concerns around cost and equity. Plan S attempted to propose an APC cap as a form of price control. Transformative agreements were proposed as a way to bridge subscriptions to OA publishing models, and as a way to formalize libraries' support of OA publishing on behalf of authors. SCOAP3 created a collective governance model of over 3,000 participating institutions to shift cost barriers away from individual authors. Subscribe to Open (S2O) models rely on reaching a threshold number of subscribing institutions to unlock research for everyone. While there are too many models to enumerate in full here, what is important to note is that the vast majority of proposed alternatives do not substantially reduce or offset the costs of OA publishing, but merely obscure the costs of publishing from authors, hiding them behind a new (but not altogether different) administrative veneer. We would go so far as to suggest that many of these alternative models

do not solve the APC Trap, but merely redirect its impact onto other academic and administrative bodies, including librarians.

For all their faults, APCs – as opposed to the alternative approaches above – render the costs of OA publishing visible and palpable to authors. Through APCs we can directly observe the cognitive dissonance caused by the current scholarly publishing landscape:

> *"[T]he system is obviously bonkers, in that we create the products, review others' products for free, and then pay to get the products published. What a world!"* (#165, UCB)

> *"The whole system is a cash cow, and we pay and pay and pay. And worse, it's literally a requirement for our academic success. And that is before you investigate equity concerns…"* (#254, UMass)

> *"It feels like legalized extortion. I am a captive audience. I need to publish my work both because that is how I get promoted and remain competitive for grant funding. . . It just feels wrong."* (#52, Pitt)

To alleviate the experience of the APC Trap for authors, it may seem like an obvious solution to subsume those costs into new subscriptions, transformative agreements, and collective funding initiatives. While these models may temporarily avert the APC Trap insofar as they obscure the costs of publishing from authors, we should not assume they solve the problem of the APC Trap. Rather, they may simply be delaying the reckoning with publication costs and entrenching existing inequities by redirecting ethical and financial burdens. When we must finally confront the trap, will we be bound too tight to break free?

# Acknowledgements

We thank Jessica Pearlman, Director of Research Methods Programs at University of Massachusetts Amherst's Institute for Social Science Research, for assistance with data analysis.

# References

1. Beasley G. Article processing charges: a new route to open access? Inf Serv Use. 2016; 36(3-4): 163-170. doi: https://doi.org/10.3233/ISU-160815.

2. Khoo SYS. Article processing charge hyperinflation and price insensitivity: an open access sequel to the serials crisis. LIBER Q. 2019 May 9;29(1).doi:10.18352/lq.10280.

3. Klebel T, Ross-Hellauer T. The APC-Barrier and its effect on stratification in open access publishing. Quant Sci Stud. 2023; 4(1): 22-43. https://doi.org/10.1162/qss_a_00245.

4. Morrison HG. MDPI pricing. [Internet]. 2018 [cited 2019 April]. Available from: https://web.archive.org/web/20180817052832/https://sustainingknowledgecommons.org/2018/05/16/mdpi-pricing-thanks-to-mdpi-ceo-franck-vazquez-phd/.

5. Jain VK, Iyengar KP, Vaishya R. Article processing charges may be a barrier to publishing. J Clin Orthop Trauma. 2021;14: 14-16. https://doi.org/10.1016/j.jcot.2020.10.039

6. Maggio LA, Chtena N, Alperin JP, Moorhead L, Willinsky JM. (2024). “The best home for this paper”: A qualitative study of how authors select where to submit manuscripts. Perspect Med Educ. 2024 Sep 9;13(1). doi:10.5334/pme.1517

7. Warlick SE, Vaughan K. Factors influencing publication choice: why faculty choose open access. Biomed Digit Libr. 2007 Mar 9;4(1). doi:10.1186/1742-5581-4-1.

8. Demeter M, Istratii R. Scrutinising what open access journals mean for global inequalities. Pub Res Q. 2020;36: 505–522. https://doi.org/10.1007/s12109-020-09771-9

9. Borchardt R, Schultz T, Dawson D. Financial and other perceived barriers to transitioning to an equitable no-publishing fee open access model: A survey of LIS journal editors. Coll Res Libr. 2024; 85(1): 96. https://doi.org/10.5860/crl.85.1.96

10. Cantrell MH, Mezick J, Estill M, Caldwell R, Collister LB. An APC Trap? Privilege and the perception of reasonableness in open access publishing. J Libr Sch Commun. 2025; 13(1): eP18184. https://doi.org/10.31274/jlsc.18184

11. Dimensions. Definitions of Publications data fields in Dimensions [Internet] 2025. [cited 2025 October]. Available from: https://dimensions.freshdesk.com/support/solutions/articles/23000018864-definitions-of-publications-data-fields-in-dimensions-details-page-and-export-

12. Crawford W. Gold open access 2013-2018: Articles in journals (GOA4). [Internet]. Cities & Insights Books. 2019. Available from: https://waltcrawford.name/goa4.pdf

13. Cantrell, MH, Collister, LB. The status quo bias and the uptake of open access. First Monday. 2019; 24(7). https://doi.org/10.5210/fm.v24i7.10089

14. Pieper D. et al. OpenAPC data [Internet] 2025. [cited 2025 July]. Available from: https://treemaps.openapc.net/apcdata/openapc/.

15. Thelwall M, Buckley K, Paltoglou G. Sentiment strength detection for the social web. J Assoc Inf Sci Technol. 2011 Oct 13;61(12). doi:10.1002/asi.21662.

Revised Manuscript with Track Changes

# "The system is obviously bonkers": The APC Trap and the bind of scholarly publishing across four ~~R1 institutions~~research intensive institutions in the U.S.

Melissa H. Cantrell[1¶]*, Rachel Caldwell[2¶], Jennifer A. Mezick[3¶], Matthew Estill[4¶], Lauren B. Collister[5¶]

**Commented [MC1]:** The footnotes show up in the desktop version of the Word doc and appear when you click on the footnote number. I'm not sure how to make them appear as text on this page.

[1] University Libraries, University of Colorado Boulder, Boulder, Colorado, United States of America

[2] University Libraries, University of Massachusetts Amherst, Amherst, Massachusetts, United States of America

[3] University Libraries, University of Tennessee Knoxville, Knoxville, Tennessee, United States of America

[4] University Library System, University of Pittsburgh, Pittsburgh, Pennsylvania, United States of America

[5] Invest in Open Infrastructure

* Corresponding author

¶ These authors contributed equally to this work

E-mail: melissa.cantrell@colorado.edu

# Abstract

This study analyzes R1 authors' the cognitive dissonance displayed by authors at research intensive institutions in the U.S. with regard to the scholarly publishing of Open Access (OA) publications, which was noted in a previous study on the sources of article processing charges (APCs) for authors of Open Access (OA) journal articles. A qualitative analysis of 154 open-ended responses was conducted. Eight themes were first identified, then each response was coded for the themes. The themes themselves coalesce around a phenomenon the authors of this study term the article processing charge (APC) Trap, in which privileged and well-resourced researchers in the U.S. experience highly contradictory sentiments about Open AccessOA publishing. The resultresults of the cognitive dissonance characteristic of the APC Trap are strong feelings of conflict as well as powerlessness amongst researchers. The qualitative analysis reveals the APC Trap as a deeply layered phenomenon, whose manifestation is dependent on positionality, as revealed throughincluding demographic features, as well as throughand interactions between different APC Trap themes. While much has been made of the failings of the APC model of open access OA publishing for under-resourced researchers, this study reveals the ways in which this model is unsustainable even for privileged researchers and countries most well-positioned to participate within it.

Commented [MC2]: use acronynym or not?

Commented [la3R2]: Spell it out here

# Introduction

This paper examines the complex attitudes and experiences of researchers regarding article processing charges (APCs) in open access (OA) publishing. Building on previous quantitative findings that demonstrated cognitive dissonance among researchers who show a strong willingness to publish OA while viewing APC prices as increasingly unreasonable and unfeasible, this study explores the qualitative dimensions of how researchers navigate APC-based publishing decisions

Commented [la4]: References order after my edit:
1: NEW https://doi.org/10.3233/ISU-16081
2: Formerly 3
3: Formerly 4
4: Formerly 2
5/6/7/8/9: Same
10: Formerly 1

Then Dimensions and the rest stay the same.

This paper examines the complex attitudes and experiences of researchers regarding article processing charges (APCs) in open access (OA) publishing. The APC model's introduction is usually attributed to BioMed Central in the year 2000 as a means to support financial sustainability for publishers who offer open access to scholarly articles in lieu of subscription revenues [1]. The problematization of the APC-based business model began shortly after its introduction. Authors initially approached APCs as a binary choice of whether to pay or not: "once authors are willing or able to pay an APC, [...] they are willing to pay them with little regard to the size of an APC" [2]. Publishers report pricing APCs based on journal prestige rather than production costs, indicating little market control over hyperinflation [3]. APC hyperinflation was documented at 5-10% annual increases with little impact on growth at major publishers or evidence for need to increase prices [2].

Beyond the pricing models, literature has consistently highlighted how APCs in general create "barriers" excluding researchers from less well-resourced institutions and countries [1,2,4,5] and reinforcing existing inequities [3,6,7,8]. Journal editors view APCs as barriers to inclusivity, expressing concern that APCs replicate financial inequalities and disadvantage under-resourced authors, particularly from the Global South or smaller institutions [9]. While authors at institutions with OA budgets viewed OA as complementary to other factors like reputation and editorial practices, those without OA budgets saw APCs as major deterrents to publishing and making their research widely available. In a 2021 study of researchers from India, authors with institutional support viewed OA favorably, with one researcher explaining: "Sometimes I think about open access as my first choice...because I'm coming from a low-resource country. And I know how people there suffer in finding the information." Conversely, unsupported authors reported: "I simply won't consider those journals because I just can't [pay] those fees...even if it's probably the best journal for me" [5]. While researchers value OA in principle, cost remains a major deterrent when not covered by grants or institutions [6].

**Commented [la5]:** Add line ~59 and following here, reorganize

**Commented [la6R5]:** Melissa - can we add a reference to this article in the references list? https://doi.org/10.3233/ISU-160815

**Commented [MC7R5]:** Ok, I added this - all the references will still need to be re-ordered once we are done

**Commented [lb8]:** Change reference to 2 - Khoo

**Commented [lb9]:** Change reference to 3 - Klebel

**Commented [lb10]:** Change reference to 2 - Khoo

**Commented [lb11]:** Change reference to 1, 2, 4 (Morrison), 5 (Jain),

**Commented [lb12]:** Change reference to 3 (Kleber), 6 (Maggio), 7 (Warlick).

**Commented [lb13]:** Reference is 8, Borchardt et al.

**Commented [MC14R13]:** 9 = Borchardt

**Commented [lb15]:** Change reference to 5, Jain

**Commented [lb16]:** Change reference to 6, Maggio

This study explores the navigation of the open access publishing landscape by authors who encountered APCs. In previous work, the authors conducted a survey to understand the resources that authors used to pay for APCs [10]. In addition to the quantitative findings, the responses to open-ended questions in the survey demonstrated cognitive dissonance among researchers who show a strong willingness to publish OA while viewing APC prices as increasingly unreasonable and unfeasible. These findings aligned with observations of the "APC effect" introduced by Klebel & Ross-Hellauer [3]. The "APC effect" describes how better-resourced institutions publish in journals with higher APCs and higher perceived prestige. In fact, institutions in wealthier countries pay 42% higher average APCs, creating stratification where less-resourced institutions are blocked from costly, prestigious publications. These charges thus have "a chilling effect on opportunity and equality for researchers from less prestigious or less wealthy institutions" [3]. While publishers offer waivers for some low- and middle-income countries, many countries like India don't qualify despite researchers facing substantial constraints. In one study, doctors and researchers reported having plenty of clinical material and the potential to write and publish, but APCs limited their options to submit academic papers [5].

**Commented [lb17]:** Change reference to 9, Cantrell

**Commented [lb18]:** Should be 3 now

**Commented [lb19]:** change to 3, Klebel

**Commented [lb20]:** 5, Jain

The existing literature has documented the APC effect specifically leading to the exclusion of under-resourced researchers. ~~Our research extends this thinking by examining well-resourced R1 institution researchers' perceptions directly. Authors report simultaneous price insensitivity and price outrage toward OA models, which results in a bind for researchers who feel obligated to use APC models despite strong feelings about their unjust impacts. This complex thinking is characterized by the "bind" of the APC Trap—a contradictory situation where APC "barriers" combine with professional anxieties and equity concerns. This results in a feeling of inability to change the status quo, even among the world's most privileged and well-resourced researchers. This article explores how the APC Trap manifests in qualitative findings, its disciplinary and~~

institutional variations, and how it represents a difficult bind without easy solutions. Our qualitative findings extend this concept. While the APC effect represents a barrier for many researchers globally, we posit a complementary "APC Trap" that binds privileged researchers who feel compelled to participate in OA publishing while simultaneously feeling powerless to change its inequities. We investigate this “APC Trap” by using our survey results, which include direct comments from well-resourced researchers about their experiences and perspectives with APCs. The researchers surveyed in our study report simultaneous price insensitivity and price outrage toward OA models, which results in a bind for those who feel obligated to use APC models despite strong feelings about their unjust impacts. This complex thinking is characterized by the "bind" of the APC Trap—a contradictory situation where APC "barriers" combine with professional anxieties and equity concerns. This results in a feeling of inability to change the status quo, even among the world's most privileged and well-resourced researchers. This article explores how the APC Trap manifests in qualitative findings, its disciplinary and institutional variations, and how it represents a difficult bind without easy solutions. We present this evidence of the ethical compromise of APCs for well-resourced researchers to demonstrate that the entrenched APC model is not working for *any author,* even those who have the easiest path to pay and the greatest benefit.

# Materials and methods

This study is drawn from a dataset collected in 2022 about publications from four United States (U.S.) research intensive universities ((Carnegie Classification of R1)in 2022. For full details about the dataset, see our first paper (citation here)[10]. The fFour major researchesearch intensive universities in the U.S.(Carnegie Classification of R1) participated in this studyare: the University of Colorado Boulder (UCB), the University of Massachusetts Amherst (UMass), the

University of Pittsburgh (Pitt), and the University of Tennessee Knoxville (UTK). These institutions are all public universities and are geographically situated in different areas of the U.S. They have similar academic profiles with degree and research representation across disciplines with the exception to hospital-based medical research. The University of Pittsburgh has a large medical school that is included in this study. The other institutions in this study exclude medical school researchers from the data because those medical schools report through different campuses with different administrative and budgetary structures.

The study protocol underwent a multi-site IRB approval, with the University of Colorado Boulder serving as the primary IRB review institution. Co-Investigators at the other institutions also submitted protocols for supplemental IRB approval, and the one Co-Investigator not affiliated with an institution of higher education submitted an Individual Investigator Agreement. The study was waived as "Exempt" by all four institutions. Data were collected via a Qualtrics questionnaire, and the recruitment period was from October 31, 2023 and November 21, 2023. Written informed consent was obtained by having respondents read and accept an informed consent statement prior to completing the survey.

After the data were collected, the dataset was organized by institution and grouped into OA types: Gold OA and Hybrid OA. These categories were based on reporting categories in Dimensions, a proprietary, comprehensive scientific research database [11]. For further details, see the Methodology in our previous paper [10]. Each of these categories was then divided into three broad academic areas: Humanities and Social Sciences (HSS), Health and Medicine (HM), and Natural Sciences and Engineering (NSE). These three disciplinary categories used for this study are based on the Fields of Research Codes included in the Dimensions dataset [11].

Six different surveys (two within each HSS, HM, NSE) were created using the Qualtrics platform, and these surveys were customized based on OA type (Gold OA or Hybrid OA). All surveys included the same two free-response questions analyzed for this paper. Survey-based analysis with open-ended questioning is well-suited for researching scholarly publishing practices and perceptions as an effective method for capturing nuanced attitudes and decision-making processes [9]. Manual coding of free text responses allows for theme development that incorporates complex social and institutional factors which can influence publishing behaviors [6,9]. These methods align with the field's focus on understanding motivations, barriers, and values; and provide contextual insights to inform future scholarly communication policy and practice. This study used a purposive sampling method, meaning that participants were selected based on specific criteria rather than chosen at random. This allowed the study to focus on individuals who were most relevant to the research questions--faculty authors engaged in OA publishing who were likely to have encountered an article processing charge. This approach helped ensure that the data collected would be useful and applicable to the goals of the research, which was originally to identify the ways that authors at R1 institutions pay for APCs. This purposive sampling allowed for a targeted analysis of the sentiments and funding sources of well-resourced researchers within the U.S. As the biggest users and drivers of the APC model of ~~open access~~ publishing, the sentiments of this subset of researchers may serve as a bellwether for the overall sustainability of the model globally.

While the broader study collected both quantitative and qualitative data related to faculty publishing patterns, this paper focuses on the qualitative responses to two open-ended, free-text questions included at the end of each survey. All respondents received the same open-ended questions, regardless of whether the survey was for a Hybrid OA or Gold OA journal. These questions were designed to provide space for respondents to offer additional context, express concerns, or raise issues that may not have been fully addressed in the preceding

**Commented [la21]:** Add a reference here to other survey-based studies, e.g. Maggio, Borchardt, that we already cite in this paper.

**Commented [la22]:** Here is a good place to say - these authors were likely to have paid an APC, and we chose them because of that!

survey questions. While there were 321 completed surveys, 154 of those also included usable responses to at least one of the open-ended questions at the end of the survey.

The first open-ended question asked, “Do you have any additional comments regarding authors’ ability to pay publishing fees for Open Access?” This question aimed to elicit personal perspectives, institutional challenges, or equity concerns related to APCs and the economics of OA publishing. The second question invited broader reflection and asked, “Do you have any additional comments about the topics in this survey?” This prompt allowed participants to provide feedback on the survey content, share general insights about OA publishing, or raise related issues not explicitly covered by the survey. However, there were not distinct or observable differences in how the 154 respondents answered across the two open-ended questions, so for the purpose of analysis the responses were aggregated into a single qualitative response, resulting in 154 responses total.

The response data was initially analyzed using automated sentiment analysis tools; the results of this analysis are briefly reported below in the *Sentiment analysis* section. After reviewing the results, we unanimously agreed that manual coding was the most effective method for analyzing the data, given the nuanced context of the language in the free text responses. All open-ended responses from both free-text questions across every respondent type and respondent group were consolidated into a single spreadsheet. The dataset was then prepared for analysis with each respondent assigned a unique identifier: a number and a short institution code.

The response data was initially reviewed to identify recurring patterns, concepts and sentiments to uncover underlying themes that reflect respondents' experiences, perceptions, or attitudes. Through discussion, we normalized frequently mentioned themes into 8 codes. Responses identified as representative of specific themes were included as examples in the Code Book to

guide the subsequent coding of survey responses. We also coded for an additional theme, called *Presupposition of Cost*, but determined it should not be analyzed as related to the APC Trap. The codes are discussed in the Results ~~*Code Book*~~ section. Respondents also conveyed broader sentiments, including frustration and appreciation, which, though not directly tied to the APC Trap, offer valuable context for understanding the overall conflicted discourse around APCs.

Following the development of eight APC Trap codes, each researcher independently reviewed participant responses. For each code, a "yes" or "no" was assigned, indicating its applicability to a given response. It's important to note that codes were applied to the full set of responses from a single respondent, rather than to individual statements, and encompassed data from both free-text questions. Therefore, each response could be assigned multiple codes which reflected themes demonstrated across the entirety of the response.

An iterative refinement process was used to ensure coding consistency and accuracy. After an initial coding review, the Code Book was refined to address any ambiguities we identified. Subsequently, each researcher coded the data independently. To measure agreement, an Excel-based formula was developed to calculate consensus on "yes" and "no" assignments. A code was definitively applied to a respondent if four or more researchers reached agreement. In instances of disagreement, all researchers discussed each specific case to reach a consensus, leading to further refinements of the codebook. This iterative process of independent coding, discussion of discrepancies, and codebook refinement continued until complete agreement on all applied codes was achieved.

## ~~Sentiment analysis~~

Two different sentiment analysis tools were used initially to explore how respondents feel about APCs: NVivo's Auto Sentiment Coding and SentiStrength [1011]. From the outset, we knew that sentiment analysis would not be enough to truly unpack the responses of our participants. However, the sentiment analysis did provide us with a starting point and some initial observations upon which to base our further qualitative analysis and coding.

NVivo categorized the 136 responses into four sentiment categories—very positive, moderately positive, moderately negative, and very negative—based on its automated sentiment detection. The NVivo analysis suggested that OA itself is widely valued, with positive comments often expressing gratitude for institutional support or fee waivers, and framing OA as a means of making research more inclusive and equitable. Even within these favorable responses, however, participants flagged systemic inequities and called for reforms. Moderately positive responses tended to support OA in principle while expressing concern about inconsistent fees and unequal burdens on underfunded authors. On the negative side, responses highlighted the financial strain of APCs, the perception of a “pay to publish” model, and concerns about how these systems exacerbate global and institutional inequalities. The most negative responses offered a systemic critique of academic publishing, describing it as exploitative and exclusionary, with calls for dismantling for-profit models altogether.

**Commented [MC23]:** Lauren, where did the number 136 responses come from - we received 321 surveys back, 154 of which had usable survey results

SentiStrength offered a numerical scoring approach, assigning values to the strength of positive and negative sentiments in each response. However, its results were more difficult to interpret. Several strongly negative responses were assigned scores of 0 or even positive values due to simplistic keyword associations with isolated words in the response, such as "profit" or "enjoy," without accounting for the specific context of the response. For example, critiques describing APCs as “absurd,” “unethical,” or part of an “exploitative” system were sometimes scored similarly to milder or strongly supportive statements.

~~This highlights a core limitation of automated sentiment tools when applied to complex, nuanced topics like academic publishing models: they often fail to grasp sarcasm, rhetorical structure, or compound sentiments. These features appeared often in our dataset, as would be expected when the stakes of interpretation are high and the subject matter is deeply complex and intertwined with power, equity, and institutional critique. As such, after performing this analysis, we embarked on a more careful, human-centered qualitative coding practice.~~

## Results ~~Code Book~~

Thematic analysis resulted in the identification of eight~~nine~~ major APC Trap codes. Many more themes were initially identified; however, some were not indicative of the APC Trap, and others were combined or consolidated into a single code. For example, concerns about the future of graduate students and their inability to pay, public and taxpayer concerns, and concerns for researchers with less or no grant funding are all included in the code for *Diversity or Equity*. Additionally, there are certainly additional ways respondents ~~indicated a feeling of being "trapped" by the APC system, which are not included here as they may not have been expressed by enough respondents to emerge in the qualitative results~~.

Below, we describe the eight main APC Trap codes we identified, the number of times they occurred in our dataset, along with illustrative quotes drawn from our respondents. These results provide a code book that may be of use for future research and development of this model.

## Code: Financial concern

This code was the most frequently applied in our dataset (n=52; 34%). This code focused on specific and personal financial hardships or worries on the part of the respondent with regard to paying the APC.

> *More journals are forcing open access fees if you publish with them. I publish often so it does stress me out where I'll always come up with this money from.* (#249, UTK)

> *I have ample grant and internal support; payment for me is feasible, albeit it is expensive and a drain on resources. I have colleagues with far less resources which precludes their ability to pay for open-access publishing.* (#32, Pitt)

## Code: Diversity or equity

This code was used frequently in our dataset (n=50; 30%). This code was applied to responses that included comments that APCs are hurting the ability to meet diversity and/or equity goals. Equity may refer to any number of imbalances between different parties, including references to "pay-to-play" scenarios and acknowledgement that some have deeper pockets than others. Common themes in this code included the perception that those who can’t pay for open access would suffer from lower exposure and reader access to their research, as well as increased bias towards the subjects of interest for wealthier institutions.

> *I just think it is inherently unfair that the public doesn't have open access to ALL published materials, especially when the opportunity to pay for publishing is not equally distributed among faculty. We need a better system! As you can see by my answers*

*above, I object to the fees on principle, not because of the cost. Why should I benefit from paying a fee that others' can't afford? Why would I want this to be a reason why my work is used/cited by others? It should be read and used/cited on its merits, not some artifact of gamesmanship or privilege.* (#25, Pitt)

*It's a diversity and equity issue. People from historically marginalized groups are least likely to be able to pay publication fees. Plus, publishing companies make LOTS OF MONEY and do not need these fees to be this high.* (#158, UCB)

## Code: Threshold of reasonableness

This code was used ~~46 times (28%)~~ in our dataset, most commonly when respondents indicated that there was an upper limit of their tolerance for APCs. This theme is broader than *Financial Concern* (which focuses on specific, personal concerns for affording a specific fee) and can include more general statements about the state of the field and questioning the reasonableness of the current system of APCs in OA publishing. This code also applies to responses that call out the exploitative or excessive practices of publishers, implying that there are “reasonable” limits for fees and profit.

*I recognize that there is labor involved in the publishing industry, but the costs to publish are out of control. Especially when you consider the profit margins enjoyed by these publishers.* (#272, UMass)

*I want to emphasize that help from the University and the departments is greatly appreciated. The cost of publishing has gone up significantly in the last 5 years that it is very difficult to find that kind of money. The amount needed to publish is very*

*unreasonable and I do not understand why the cost has increased. It is very frustrating to investigators like myself, who want to publish in high impact journals, but, cannot always do so.* (#227, UTK)

## Code: Restriction of decisions

This code was used 40 times (24%) in our dataset. This code indicates that respondents made choices they might not otherwise have made because of an APC – specifically, that they had to make a choice for themselves, not that unnamed other parties have their decisions restricted. Restrictions included mandates or grant requirements that restrict a possible set of publication outlets, or choosing different journals due to an APC that made a particular outlet unaffordable. This code often indicates a sense of powerlessness.

> *I have a well-funded lab and access to a decent amount of discretionary funding to pay for publishing fees, but then I often struggle because the highest-impact / most valued journals have the highest publishing fees, meaning that sometimes I have to choose between a high-valued venue and a venue I can afford.* (#170, UCB)

> *I felt a bit trapped into the high fees ($3090) for this journal. We had originally submitted to a different (higher impact Nature family journal, which I think could have been worth the cost for its scope and recognition. But, after review, the editorial staff suggested we transfer to a more specialized Nature family journal. At that point, it would have been too time consuming and cumbersome to pull the paper and resubmit to a completely different journal, so we went ahead. But the fees for this journal do NOT match its standing or reputation, and I could have easily submitted this article to a no cost PLoS journal if I had realized earlier how much this journal would cost.* (#194, UTK)

## Code: Grants don’t cut it

This code was applied to responses (n=35; 21%) that specifically indicate limitations in grant funding, whether grant funds were used to pay for the APC or not. This code often referred to grant funds not being enough for all that the respondent needed to do for their research or academic expectations. This code was not applied to acknowledgements that some others don't have grant funds to cover these costs (which would be coded Diversity/Equity). One theme that recurred in this code surrounded the logistics of paying APCs with grants and the various hurdles and barriers that caused for publications.

> *Ultimately seems like a good model, but in the near term we are increasingly shifting the burden of publication costs from less-publishing consumers (industry, non research-intensive academic) to grants, without most funders increasing grants to keep up (just one of many costs of research growing faster than grants) which makes things challenging. As a result I try to avoid paying open access fees whenever possible.* (#300, UCB)

> *People with NIH* [National Institutes of Health] *and NSF* [National Science Foundation] *money get much more and are required to publish open access. USDA is not that case and all of my funding comes from that, leaving me to use valuable discretionary funding that could be used for other important purposes (equipment, equipment maintenance, etc.)* (#274, UMass)

> *There is a Catch-22 when it comes to using grant funding to cover the APC. Typically we are submitting manuscripts for publication at least a year after our research funding*

> *expires. Therefore we can't pay the APC from grant funds unless the NIH allows us to retain that money after we close out the grant.* (#95, Pitt)

## Code: Academic obligation/expectation

Responses coded for *Academic Obligation/Expectation* (n=33; 20%) indicate a strong expectation or obligation to publish OA for professional advancement. These respondents mention pressures surrounding promotion, publication impact, student benefits, or to conform with disciplinary norms. This code is similar to Restriction of Choice but often without a specific decision point, and often indicates a feeling of being trapped by expectations around the job.

> *IF journals require publishing fees, researchers should be allotted money for this since it is a requirement of my job.* (#156, UCB)

> *APCs should be supported by Dept./College/University because publishing is an expectation/requirement of their job and benefits the institution's reputation.* (#221, UTK)

## Code: Academic labor

The *Academic Labor* code (n=28; 17%) references responses about academic work (including peer review) that is often done for free and without benefit, while a journal or publisher profits from APCs. These responses often include references to merit with journals (such as a waived fee as a reward for labor) and volunteerism required by some journals.

> *I agree with open access publishing, but I do not understand why the onus is on investigators to pay instead of publishers. Journals receive free peer review, pay next to nothing for editorial board stipends, receive ad revenue, and now have small publishing*

*costs as print journals are dwindling. It would be reasonable if investigators covered only costs related to editorial stipends, copy editing, and online hosting (assuming that publishers will provide these data transparently).* (#40, Pitt)

*The publishing business is very difficult to understand. We, researchers, write and review the papers that are published for free. The publishing houses control the logistics and nothing else, for which we pay them. Then, the authors receive no royalties and the publishers get all the profits; that seems unfair.* (#152, UCB)

*We provide peer-review service, editorial service.. Journals APCs are legalized thevery [sic], esp. at high-end range.* (#327, UTK)

## Code: Quality or rigor

Several (n=20; 12%) respondents mentioned concerns that OA reduces the quality or integrity of the research. This code often co-occurs with concerns about perverse incentives for publishers to accept papers of dubious quality from authors who can pay the fee, or with speculation that high Impact Factor journals or those that are perceived as elite are exploiting their perceived high-quality status to charge higher APCs.

*I don't like the model where researchers pay a lot (in some journals) to publish their work - it creates bad incentives to publish bad research in some cases, and makes it so people with less financial backing cannot have their papers openly accessible in others.* (#164, UCB)

*Page charges and open access fees are being exploited by so-called elite journals because of their perceived status. Individuals are more than willing to pay $5-10K or*

*more to publish in Nature and Cell title journals often because of careerism. Members of promotion and tenure committees or grant review panels are often so specialized that they cannot evaluate science outside of their narrow areas. So having the imprimatur of Cell or Nature in the journal title gives the allure of significance or importance. This is corrupting.* (#6, Pitt)

## Presupposition of cost

As part of the analysis, responses were also coded for *Presupposition of Cost*, meaning that the respondent ~~equated~~conflated OA with an article processing charge or other fee. Several of our respondents positioned payment for OA publication as an expected fact about the world, although about 71% of the journals in the Directory of Open Access Journals do not charge article processing fees as part of their business model [12]. This is a linguistic phenomenon known as *presupposition:* speakers assume that readers know some information and reference that information when discussing other information. This particular presupposition that OA requires a publication fee has been noted in other research [13]. ~~However, in this work, this code was not analyzed ,~~as part of the APC Trap for two reasons: first, because this section of the survey was primarily answered by authors who had paid an APC for their publication (and, therefore, the existence of the cost was in fact true), and second, the wording of the question contained a presupposition of cost in itself (“Do you have any additional comments regarding authors' ability to pay publishing fees for open access?”).

It remains essential to highlight this presupposition, as it reveals a trend in conversations and marketing surrounding OA. Conflating OA (a model for the availability and use of research articles) with article processing charges (a business model for publishing) can not only entrench beliefs about publishing options but also complicate or obfuscate the purpose of publishing OA [13]. The conflation of OA with APCs can make the APC Trap feel more like an Open Access

**Commented [MC24]:** First Monday paper?

**Commented [MC25]:** First Monday paper

Trap, erasing the potential of Diamond OA (that is, publishing without fees to the author or reader) and other models, tarnishing the practice of open research by equating it to complex market and privilege dynamics illustrated by the other components of the APC Trap.

Below are some examples of responses that contain a *Presupposition of Cost* for illustrative purposes.

> *This discussion is useless. There is a cost to publish a paper, and it does not matter what people feel they would like to pay for a paper.* ~~*Somebody always paid, and with subscription journals, libraries pay for it - i.e., us.*~~ (#198, UTK)

This example from respondent 198 contains a clear presupposition using the construction "There is": "There is a cost to publish a paper". This presentation of a fact presupposes that this is a truthful state of the world. Linking the subscription cost to publication costs is a type of presupposition often used in publisher marketing – to suggest that if they are not generating revenue by charging a fee for a subscription, then they must charge a different fee. This response was not coded for any other codes associated with the APC Trap, indicating that this presupposition is not always linked to the APC Trap.

*Presupposition of Cost* can co-occur with other APC Trap codes. In this response from #181, UTK, there are two other aspects of the APC Trap: *Grants Don't Cut It* and *Academic Obligation/Expectation*:

> *[O]pen access is mandatory now in projects that are federally funded. The open access model drains the grant funds because my last paper cost more than 3k. The Open*

*Access fund would only pay 1800 of this and there is no guarantee of this funding.* (#181, UTK)

This example contains the presupposition “[t]he open access model drains the grant funds.” While it is not clear if they are referring only to one example of an APC associated with the publication of an article draining grant funds, the reference to the “open access model” presupposes that the model itself, and not, for example, a certain instance of that model, is to blame.

Presuppositions can also occur when expressing nuance around expectations for costs; these instances sometimes co-occur with the *Threshold of Reasonableness* APC Trap code:

> *It is acceptable for open access journals to require publishing fees since they cannot obtain revenue from subscriptions or advertisements. However, those that only publish online should have lower fees (<$750) compared to those that publish online AND in print (<$1500).* (#60, Pitt)

This example contains a presupposition of revenue associated with journals, whether through subscriptions, advertisements, or publishing fees. In this case, the *Threshold of Reasonableness* code applied to this response means that while the respondent might accept the existence of a publishing fee, they still exhibit a sense of the APC Trap by expressing a preference for lower fees than the reported average.

## ~~Results and d~~Discussion

Out of 321 total responses to the survey, 154 had a usable response to at least one of the two open-ended questions: “Do you have any additional comments regarding authors' ability to pay

publishing fees for open access?", and "Do you have any additional comments about the topics in this survey?" By "usable," we mean that the text field contained substantive content beyond responses such as "No comment" or "N/A."

~~After reaching a threshold of agreement for codes on all responses, we were able to analyze the results to surface themes across the APC Trap elements. In this section, we first analyze the coding of the qualitative responses through quantitative analysis, then demonstrate the layered nature of the APC Trap by analyzing these codes alongside the other survey questions as well as alongside other codes.~~

Our thematic analysis of qualitative data identified several core motifs around which authors were fixated in relation to APCs, and which worked together to reveal a contradictory but complementary sense of obligation and exasperation over paying for OA publishing. It is this bind - this acute cognitive dissonance - that we coined as the APC Trap.

## APC Trap elements

Across all respondents to the survey who provided a usable open-ended response (n=154), 90% of those respondents' answers demonstrated at least one APC Trap theme (n=139), indicating that the APC Trap is a phenomenon experienced by R1 authors in a wide array of contexts. Fig~~ure~~ 1 shows the totals for the number of APC Trap elements coded within a single response.

**Fig 1. Responses by number of APC Trap elements.**

**Formatted:** Space Before: 12 pt

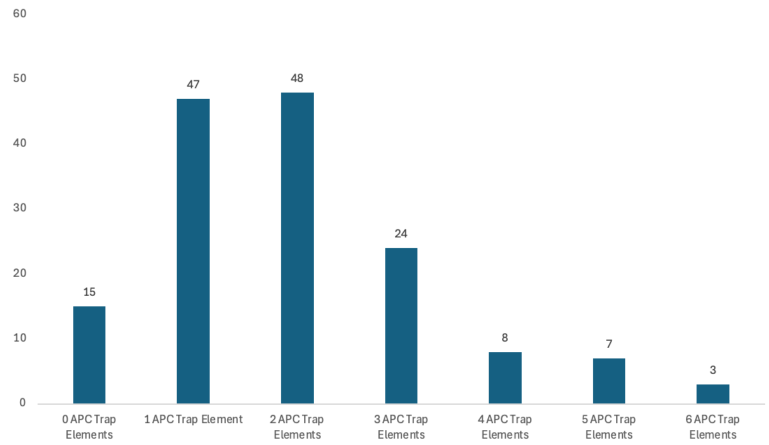


~~**Fig. 1. Responses by number of APC Trap elements.**~~

**Formatted:** Font: Bold

**Formatted:** Font: Bold, English (United States)

Most commonly, responses contained one or two APC Trap elements, with 62% of responses (n=95) falling into these categories. Twenty-seven percent of responses (n=42) contained three or more APC Trap elements. There were 15 responses which did not demonstrate any of the eight elements we identified, while 3 responses demonstrated six elements within the open-ended responses. No responses demonstrated seven or more APC Trap elements.

Example response with no APC Trap Elements:

*"I like the idea of a University fund to support publication costs."* #94, Pitt

Example response with six APC Trap Elements:

*"I really think these fees have gotten out of hand. Especially for junior researchers who may have little or no extramural funding, the fees present a dilemma. You have to publish to advance, but if you run out of money you won't be able to continue your research, and you won't advance. For the particular journal article that this survey referenced, the publisher offered to waive the fee if we submitted our work to that*

> *particular journal. This was the determining factor for me. If I hadn't had to choose between one journal that didn't charge a fee and a handful of others that would charge $3,000+ (!), I probably would have submitted to one of the other journals. But since I really felt like I needed to conserve resources, I opted for the journal that would waive the fee. Not a great system. I also really don't understand where these fees go/what they are funding. They seem exorbitant for, what exactly? Putting my document into a template, loading it on a website, and adding it to some databases?"* #36, Pitt

Table 1 shows the total number each element was coded as present in a response, as well as the percentage of responses out of 154 for each element.

**Table 1. Totals for each APC Trap element, and percentage of total responses.**

**Formatted:** Font: Bold

| **APC Trap Element** | **Total** | **Percent of Total** |
|---|---|---|
| Financial Concern | 52 | 34% |
| Diversity or Equity | 50 | 32% |
| Threshold of Reasonableness | 46 | 30% |
| Restriction of Decisions | 40 | 26% |
| Grants Don't Cut It | 35 | 23% |
| Academic Obligation/Expectation | 33 | 21% |
| Academic Labor | 28 | 18% |
| Quality or Rigor | 20 | 13% |

~~Table 1. Totals for each APC Trap element, and percentage of total responses.~~

*Financial Concern* was the most common theme coded, with slightly more than a third (34%) of respondents expressing personal financial hardship. This may be due in part to respondents interpreting the first open-ended question about "authors' ability to pay publishing fees" as a question about their own ability to pay. About a third (32%) of responses also included *Diversity*

*or Equity* concerns, demonstrating that the inequity of APC-based OA models is top of mind for many researchers.

## Demographics

Demographic factors about respondents were gathered prior to the survey and were not based on respondent self-selection. There were some distinctive demographic variations in both the extent to which the APC Trap was experienced and the types of concerns which were most prominent.

Tables 2-4~~2 through 4~~ display the average number of APC Trap elements coded based on these demographic factors, rounded to two decimal places.

**Table 2. Average APC Trap elements by discipline.**

| Discipline | Average APC Trap Elements |
|---|---|
| Humanities and Social Sciences~~SS~~ | 2.21 |
| Natural Sciences and Engineering~~SE~~ | 1.95 |
| Health and Medicine~~HM~~ | 1.95 |

~~Table 2. Average APC Trap elements by discipline.~~

**Table 3. Average APC Trap elements by institutional affiliation.**

| Institution | Average APC Trap Elements |
|---|---|
| University of Massachsetts~~Mass~~ | 2.59 |
| University of Colorado Boulder~~CB~~ | 2.06 |
| University of Pittsburgh ~~Pitt~~ | 1.97 |
| University of Tennessee Knoxville~~UTK~~ | 1.59 |

~~Table 3. Average APC Trap elements by institutional affiliation.~~

**Formatted:** Font: Bold

**Formatted:** Space Before: 12 pt

**Formatted:** Space Before: 12 pt

**Formatted:** Font: Bold

Table 4. Average APC Trap elements by OA type.

| Open Access (OA) Type | Average APC Trap Elements |
| --- | --- |
| Hybrid | 2.21 |
| Gold | 1.94 |

Table 4. Average APC Trap elements by OA type.

HSS demonstrated a 12.5% higher rate of APC Trap elements, on average, than the other two disciplinary categories. By institution, UMass responses demonstrated by far the largest number of APC Trap elements on average. Responses from authors of Hybrid articles demonstrated slightly higher average APC Trap elements, at 13% more APC Trap elements, on average, than responses from authors of Gold articles.

Figure 2 shows a disciplinary breakdown of responses that include APC Trap elements. Respondents in HSS disciplines, as well as respondents of Hybrid OA articles, experienced the APC Trap more acutely than their peers. While we cannot make broad-stroke generalisations since each respondent is unique in their experience of the APC Trap, it is likely these groups experience more financial pressure and fewer alternative funding options than their peers in this study. HSS authors are less likely to have available grant funding. According to the OpenAPC dataset hybrid articles have significantly higher average APCs, placing additional financial pressure on authors [14]. This suggests that lack of institutional funding and broad OA initiatives impacted respondents who felt more financial strain or a restriction of decisions when paying these costs.

**Fig 2. Percent of APC Trap elements by discipline.**

Formatted: Font: Bold

Formatted: Space Before: 12 pt

Formatted: Font: Bold

Formatted: Space Before: 12 pt, Line spacing: Multiple 1.15 li

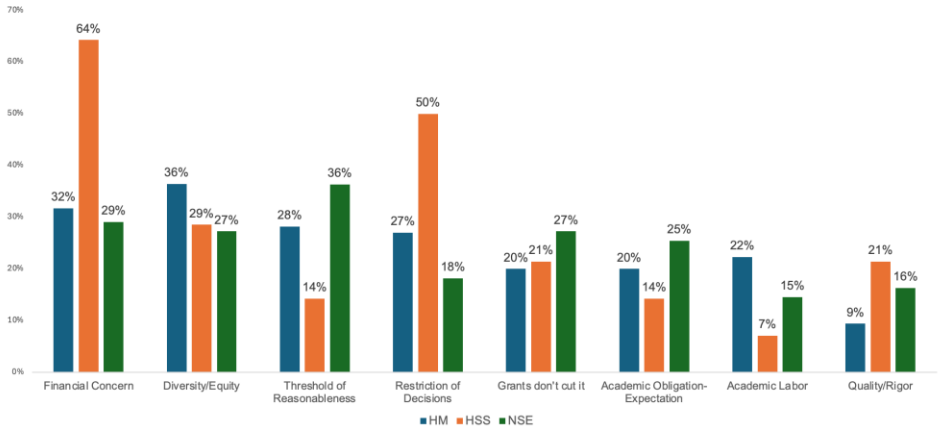


~~**Fig. 2. Percent of APC Trap elements by discipline.**~~

Formatted: Font: Bold

HSS responses were twice as likely as HM and more than twice as likely as NSE responses to display *Financial Concern*. Similarly, HSS responses were almost twice as likely as HM and more than twice as likely as NSE responses to display a *Restriction of Decisions. Academic Labor* was by far the least likely element to appear in HSS responses.

For NSE respondents, *Threshold of Reasonableness* was the most significant APC Trap element, with over one third (36%) of HM responses demonstrating this code, followed by *Financial Concern*, *Diversity or Equity* and *Grants Don't Cut It*. *Academic Labor* was the least coded element in NSE responses.

*Diversity or Equity* was the most common APC Trap element for HM respondents, with more than a third (36%) of responses demonstrating this code, followed by *Financial Concern* and *Threshold of Reasonableness*. *Quality or Rigor* was the lowest concern demonstrated by HM responses. *Financial Concern* was either the top or second-highest concern across all disciplines.

Generally, HSS respondents were twice as likely to experience the “personal” trap elements (*Financial Concern* and *Restriction of Decisions)*, while the other disciplines were more likely to focus on the “systematic” concerns of OA publishing not being reasonable or equitable for everyone (*Threshold of Reasonableness*, *Diversity or Equity*, *Academic Labor*). This suggests that the APC Trap exists on a kind of spectrum: If an author can’t pay to begin with, their concerns are focused on how to participate within the system. If an author can pay and holds a somewhat privileged status, concerns shift to broader inequities or systemic concerns in scholarly publishing.

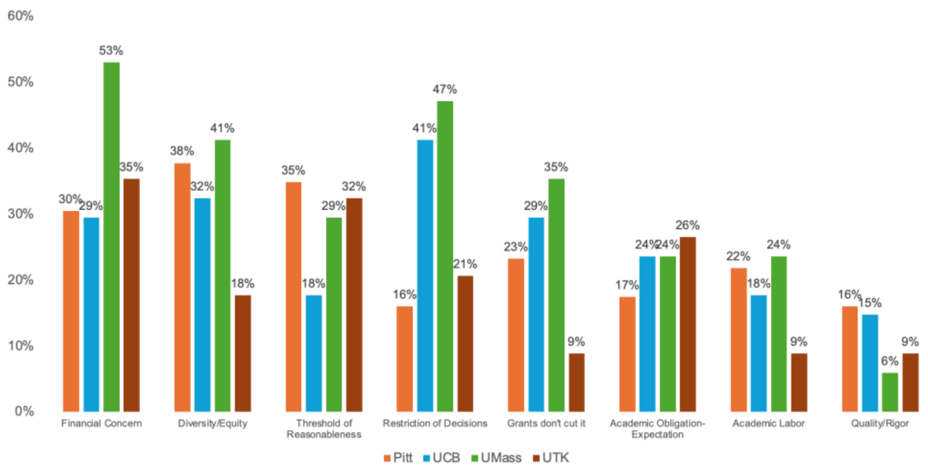


**Fig. 3. Percent of APC Trap elements by institution.**

Formatted: Font: Bold

Figure 3 shows APC Trap codes separated by institution. More than half (53%) of UMass qualitative responses expressed *Financial Concern*, while nearly half (47%) expressed a *Restriction of Decisions* around OA funding. UMass responses were also the most likely to identify *Diversity or Equity* concerns around OA publishing. In fact, out of the eight APC Trap elements analyzed for this study, UMass respondents demonstrated five of them more acutely than the other three institutions (*Financial Concern*, *Diversity or Equity*, *Restriction of Decisions*, *Grants Don’t Cut It*, and *Academic Labor)*. Even as our first study demonstrated the majority of

APCs being paid through grants, authors at institutions with fewer alternative funding sources (such as departments or a library fund) may experience additional and compounded strain over the payment of APCs.

**Fig 3. Percent of APC Trap elements by institution.**

Formatted: Space Before: 12 pt

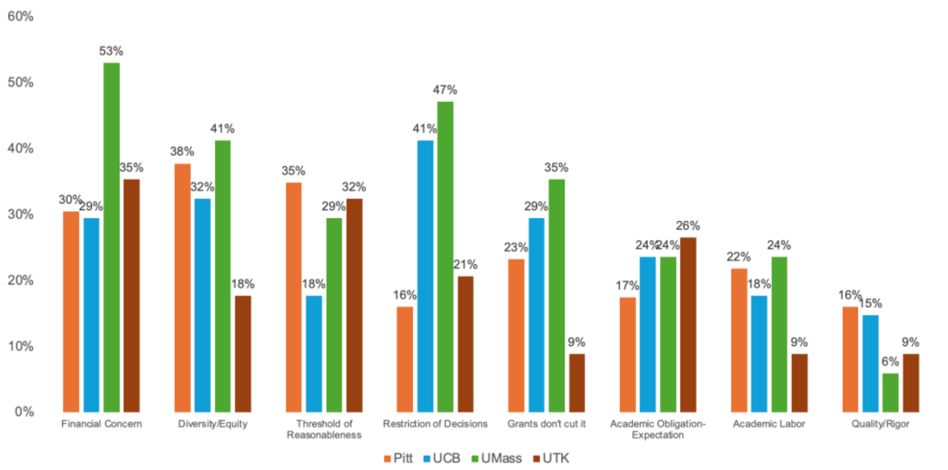


For Pitt, *Diversity or Equity* concerns (38%) was the biggest APC Trap element identified, followed closely by *Threshold of Reasonableness* (35%). Interestingly, while *Financial Concern* was the most identified APC Trap element in UTK responses, with over one-third (35%) of responses displaying *Financial Concern*, UTK also had significantly fewer responses indicating that *Grants Don't Cut It* - 9%, compared to a quarter or a third of responses at other institutions. This likely indicated that financial issues brought up by UTK responses were more often of a personal nature. For UCB responses, *Restriction of Decisions* was the most common APC Trap element identified (41%) by a fairly large margin, followed by *Diversity or Equity* concerns (32%).

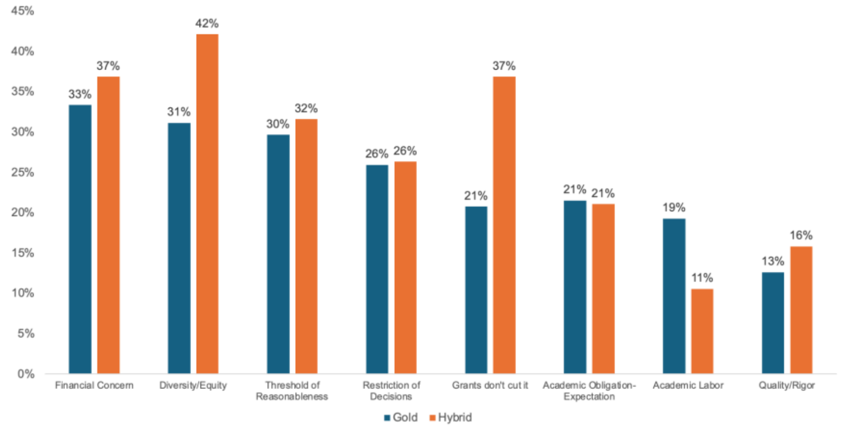


~~**Fig. 4. Percent of APC Trap elements by journal OA type.**~~

**Formatted:** Font: Bold

Fig~~ure~~ 4 shows APC Trap elements separated by kind of publication, Gold or Hybrid OA. Authors of Hybrid articles were almost twice as likely as Gold OA authors to indicate in their responses that *Grants Don't Cut It.* This can certainly be explained in part by the fact that a portion of Gold respondents did not pay an APC at all, but may also indicate the pressure of higher APCs under the Hybrid OA model.

**Fig 4. Percent of APC Trap elements by journal OA type.**

**Formatted:** Space Before: 12 pt

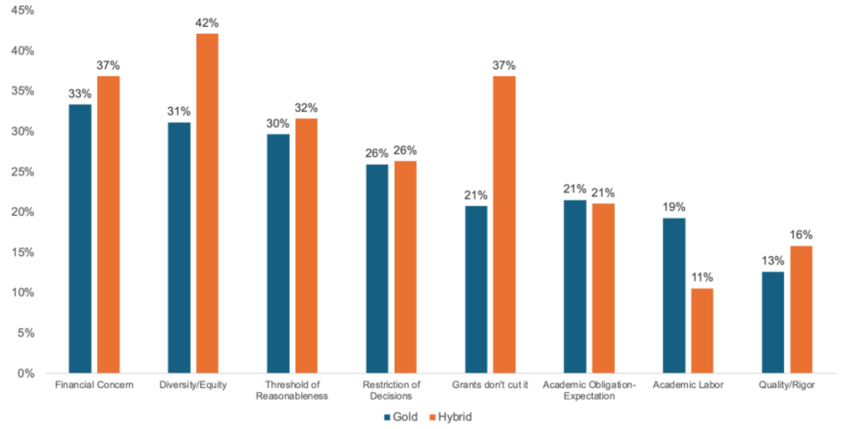


**Fig 4. Percent of APC Trap elements by journal OA type.**

In fact, respondents who published in Hybrid OA journals demonstrated every APC Trap except *Academic Labor* more frequently than respondents who published in Gold OA journals. While the cost of Hybrid OA is likely an important factor, we cannot attribute this difference solely to costs, as respondents of Hybrid OA articles were also more likely to demonstrate concerns about the *Quality or Rigor* of OA publishing, as well as concern for *Diversity or Equity* (42%), suggesting these respondents have elevated concerns about the standards and viability for such models even as they willingly participate in them.

## Sentiment analysis

In addition to the coding of the APC Trap elements, we attempted to determine the sentiment of the qualitative responses as a next logical step in understanding the respondents' points of view and concerns about the APC Trap elements. Two different sentiment analysis tools were tested: NVivo's Auto Sentiment Coding and SentiStrength [15]. Unfortunately, both tools had significant

**Commented [la26]:** Check for where this appears in the references list at the end of edits.

drawbacks that resulted in the results and analysis being unusable. These are reported here because they represent a potential limitation to this study and future avenue for investigation.

The primary drawback was that each response often flagged both tools for simultaneously positive and negative analysis. For example, NVivo categorized as “moderately positive” responses that expressed support of OA in principle with concerns about inconsistent fees and unequal burdens on underfunded authors. SentiStrength offered even more mixed results because it used a numerical scoring approach, assigning values to individual words in each response. For several responses that all authors agreed to be strongly negative, SentiStrength assigned scores of 0 or even positive values due to simplistic keyword associations with isolated words such as "profit" or "enjoy," without accounting for the context of the response.

The results from these experiments highlight a limitation of automated sentiment tools when applied to complex, nuanced topics like academic publishing models: they often fail to grasp sarcasm, rhetorical structure, or compound sentiments. These features appeared often in the analyzed dataset, since the respondents had strong emotions and the subject matter is deeply complex and intertwined with power, equity, and institutional critique. As such, a sentiment analysis is not included in this results discussion, although this may be a promising avenue for future analysis.

**Formatted:** Font: Not Bold

**Formatted:** Normal

## The APC Trap based on responses to other survey questions

Some APC Trap codes have a clear relationship with other survey questions, which can provide some indicator of sentiment of the respondent. For example, Fig~~Figure~~ 5 shows that respondents who indicated it is more difficult for them to pay are more likely to have left a comment coded as *Financial Concern*.

**Fig 5. Percent of respondents' comments coded as *Financial Concern* viewed by how respondents perceive their own ability to pay APCs relative to their peers.**

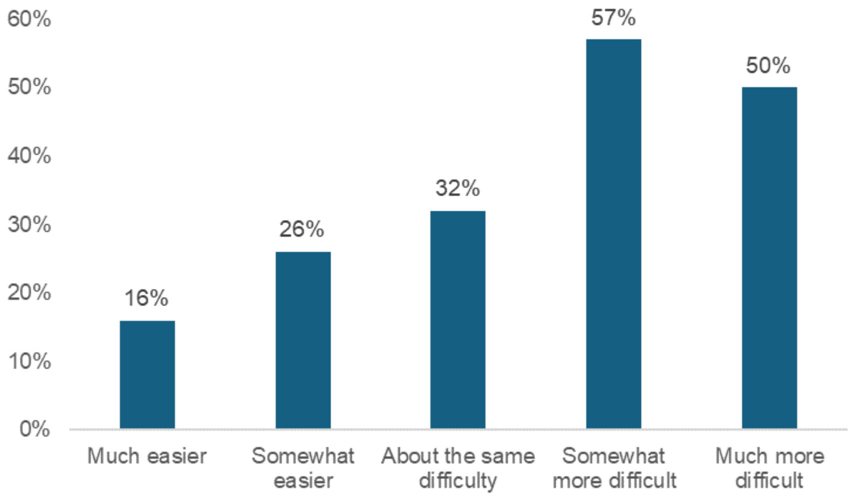


~~**Fig.5. Percent of respondents' comments coded as *Financial Concern* viewed by how respondents perceive their own ability to pay APCs relative to their peers.**~~

Similarly, when respondents said it was much *easier* for them to pay APCs, they also were less likely to leave a comment that was coded as *Grants Don't Cut It* (Fig 6). This might indicate that participants who have larger grants or significant institutional support already find it easier to pay for APCs in general because they are accustomed to financial support. The responses for those who indicate it is more difficult to pay APCs also report difficulties with support in general, as in the following response:

**Formatted:** Space Before: 12 pt

**Formatted:** Font: Bold

**Formatted:** Font: Bold, English (United States)

*"The fees keep increasing as my obligations decrease the time I have to write grants to pay for these costs! Plus it stinks to spend a month of a grad student's salary on OA. Supporting trainees is where I'd prefer to spend money."* (#139,UCB)

**Fig 6. Percent of respondents' comments coded for *Grants Don't Cut It* viewed by how respondents perceive their own ability to pay APCs relative to their peers.**

**Formatted:** Space Before: 12 pt, Line spacing: Double

**Formatted:** Font: Bold, English (United States)

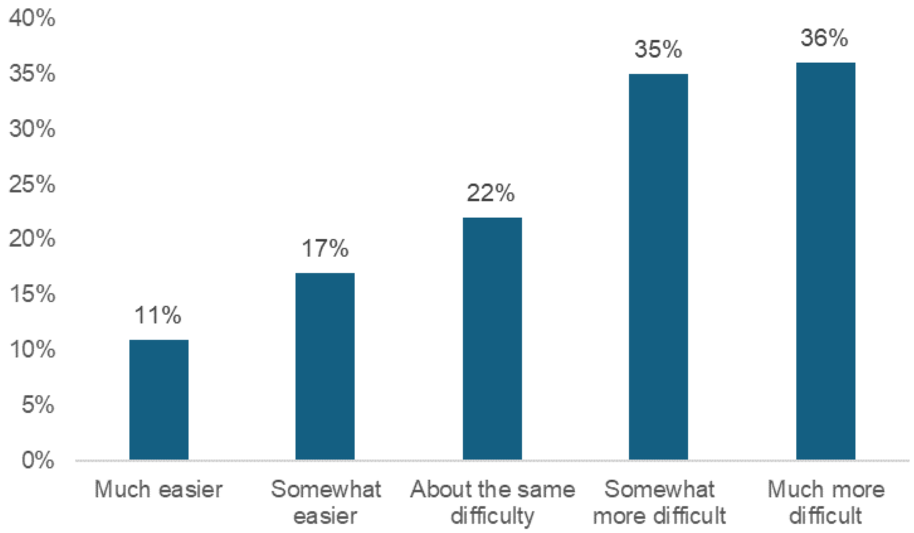


~~**Fig. 6. Percent of respondents' comments coded for *Grants Don't Cut It* viewed by how respondents perceive their own ability to pay APCs relative to their peers.**~~

**Formatted:** Font: Bold

**Formatted:** Font: Bold, English (United States)

Some other interactions of survey elements reveal additional elements of the APC Trap. Part of the APC Trap is self-awareness; that is, respondents reported a moral quandary of participating in the system of paying APCs for OA even though they knew that it was inequitable and made publishing more difficult for others. This sentiment surfaced when looking at the responses of those who reported that it was easier to pay APCs than others in their field.

Specifically, the easier that respondents thought it was for them to pay fees themselves, the more their comments involved *Diversity or Equity Concerns*. Fig~~ure~~ 7 shows the upward trend of codes for *Diversity or Equity Concerns* as the self-reported ease of paying for APCs rises. This demonstrates a correlation between awareness of ease and inequity, suggesting that perhaps at least some respondents recognized that participating in paying APCs presented a moral quandary.

**Fig 7. Percent of respondents' comments coded for *Diversity or Equity* viewed by how respondents perceive their own ability to pay APCs relative to their peers.**

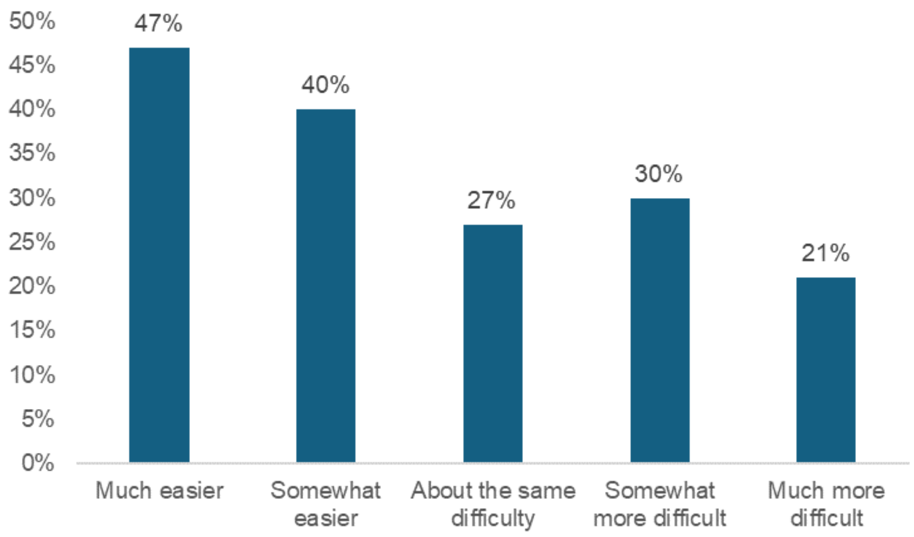


~~**Fig. 7. Percent of respondents' comments coded for *Diversity or Equity* viewed by how respondents perceive their own ability to pay APCs relative to their peers.**~~

Another APC Trap moral quandary is the interaction between APCs and perceived ethics in publishing or academic conduct. When we looked at the respondents who indicated a reasonable amount to pay for APCs, we noticed an interesting trend related to this concept.

**Formatted:** Space Before: 12 pt

**Formatted:** Font: Bold

**Formatted:** Font: Bold

Respondents who indicated any amount is reasonable (that is, those who did *not* say “no fees are reasonable”), were much less likely to demonstrate concerns about *Quality or Rigor* (Fig. 8).

However, for respondents who indicated “No fees are reasonable”, *Quality or Rigor* was the second-most prominent code in their responses (second only to *Diversity or Equity)*, present in 31% of their responses.

This alignment between a response of “No fees are reasonable” and a concern about *Quality or Rigor* may indicate conflation between article processing charges and a low-quality, review-minimal “pay to publish” model, which is a sentiment often expressed in the responses themselves. The following response provides a succinct example:

> “*I think open access tho [sic] well intentioned is a disaster. Authors should not be in the position of pay to play.” Even for those who do not want to participate in the APC system and who think that fees are completely unreasonable, they indicate a recognition that those who do participate are setting up an ethics problem in publishing related to the pressure to publish low quality materials.”* (#256, UMass)

**Fig 8. Percent of respondents’ comments coded for *Quality or Rigor* viewed by the amount respondents’ perceive is reasonable to pay for APCs.**

**Formatted:** Space Before: 12 pt

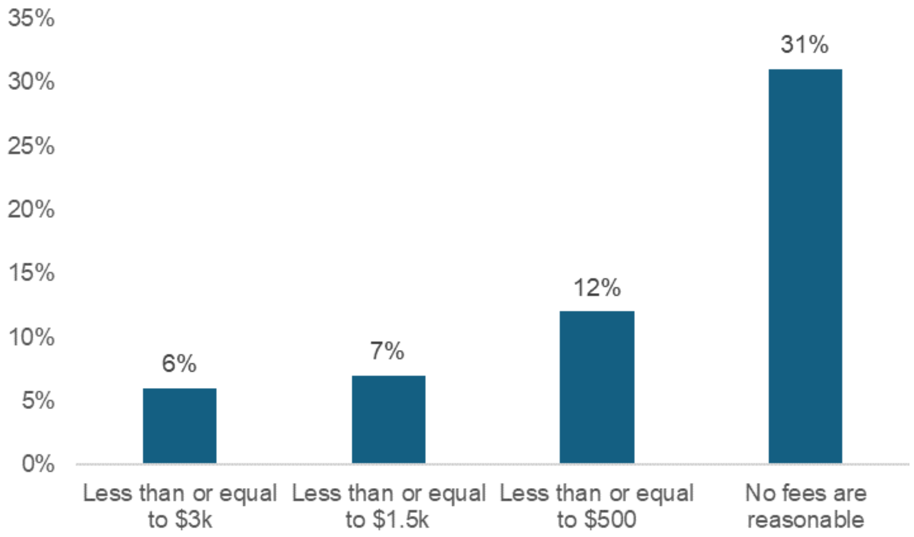


Fig. 8. Percent of respondents' comments coded for *Quality or Rigor* viewed by the amount respondents' perceive is reasonable to pay for APCs.

**Formatted:** Font: Bold

**Formatted:** Font: Bold, English (United States)

## Cross-tabulation of the APC Trap elements

In our analysis, we noted that some codes occurred together more often than others. Table 5 lists and highlights codes with a co-occurrence rate higher than 20% – in other words, out of responses coded with either of two elements, the shaded boxes in the table indicate the percent of those which coded for both exceeded 20%.

Table 5. Correlation matrix among APC Trap elements

**Formatted:** Font: Bold

**Formatted:** Space Before: 12 pt

| | Financial Concerns | Restriction of Decisions | Diversity or Equity | Grants Don't Cut It | Academic Obligation / Expecta-tion | Quality or Rigor | Threshold of Reasonabl eness | Academic Labor |
|---|---|---|---|---|---|---|---|---|
| Financial Concerns | 100% | 37% | 36% | 18% | 27% | 8% | 15% | 8% |
| Restriction of Decisions | | 100% | 13% | 25% | 24% | 7% | 16% | 10% |

| | | | | | | | | |
|---|---|---|---|---|---|---|---|---|
| Diversity or Equity | | | 100% | 13% | 14% | 21% | 10% | 11% |
| Grants Don’t Cut It | | | | 100% | 21% | 6% | 11% | 3% |
| Academic Obligation / Expectation | | | | | 100% | 8% | 18% | 7% |
| Quality or Rigor | | | | | | 100% | 12% | 9% |
| Threshold of Reasonableness | | | | | | | 100% | 21% |
| Academic Labor | | | | | | | | 100% |

~~Table 5. Correlation matrix among APC Trap elements~~

*Financial Concern* and *Restriction of Decisions* were the most likely to co-occur within a given response, with a 37% co-occurrence rate. This is not surprising considering these are the two most “personal” codes, with responses most likely to provide personal anecdotes about difficulties with publishing fees. Here are a couple of examples of this cross-section of *Financial Concern* with *Restriction of Decisions*:

> *“I had to cut my salary in order to pay the increased fee for "mandatory" open access”* (#133, UCB)

> *“I wrote an Opinion Piece, which cost about $400. The price determined what I wrote. I could not have afforded to submit an article.”* (#163, UCB)

Interestingly, *Financial Concern* and *Diversity or Equity* responses co-occurred at a similar rate (36%). This is likely due to the fact that our codebook indicates that *Financial Concern* responses may involve the respondent identifying as a class or category of people with financial strain, while the codebook’s instructions for *Diversity or Equity* are quite expansive and include

references to any number of imbalances between different parties or stakeholders. Both of these codes, in other words, deal with delineation between the self and others, of *me* and *them* (typically more or less privileged researchers). Respondents with this co-occurrence were adept at understanding the wide variation of ability and need in funding OA publications. The following responses are examples of this unique crosstabulation:

> “*The proportion of grant money that has to be set aside for APCs feels ridiculous, and unfair when that funding comes from public tax dollars and then is just shuttled straight to wildly profitable publishers. Also, publications in "good journals" are required to get funding in the first place, and this can place a strain on researchers who don't yet have funding, but need to publish.”* (#232, UTK)

> *"Open access fees really just fuel inequities in publications AND contribute to a lack of diversity in fully promoted and tenured professors at universities nationally. Those who have money can pay, be published, and get more citations, and those who don't...well don't. And then they are forced to move on to a different institution or career path. It's a huge problem from a DEI perspective in academia."* (#120, Pitt)

*Financial Concern* and *Academic Obligation/Expectation* also had a high co-occurrence rate (27%). Responses invoke both personal financial strain as well as a sense of professional expectation or pressure to publish in particular journals, whether for the sake of the respondent’s academic tenure, promotion or job retention, or for the professional recognition of students who hope to become full-fledged researchers in the future. This co-occurrence is a great demonstration of the APC Trap at work, whereby a sense of obligation is strong enough to override conflicting and often unsustainable financial strain. This is exemplified in the following response:

*"I would estimate that I'm paying $10-15k per year in publication fees because so many journals in my field have switched to open access and I want to make sure students are publishing in places that keep them competitive for future research careers. UMass right now will partially subsidize one of those publications (but I need help with 4-5 pubs per year). It is completely unsustainable and I have no idea how I will continue to pay for publications."* (#257, UMass)

In the last two high co-occurring pairs, *Restriction of Decisions* is a recurring theme. The combination of *Restriction of Decisions* and *Grants Don't Cut It*, which co-occur in 25% of responses, points to the strain multiple grant recipients expressed in their responses – even with grant funding, publishing decisions may feel forced, unsustainable, or, in the words of this researcher below, "absurd."

*"Fees of $6000 to $10000 to publish after slow reviews and turnaround times by the journal are absurd. The system is broken yet we are often required or encouraged to publish in open access journals by NIH et al but we do not have the funds in our limited budget to cover this. The fees are not sustainable with the current level of support by NIH and non-profits. These fees may eat up as much as 10-20% of a small non profit grant. How are we supposed to cover our salaries, our lab salaries, pay for space and supplieds [sic], and then also pay these absurd fees?"* (#5, Pitt)

Taking up one last example, *Restriction of Decisions* and *Academic Obligation/Expectation* – which co-occur in 24% of responses – overlap in the codebook in the sense that academic obligations often create real or perceived restrictions in publication decisions by authors. When there is an expectation within the field of study or the institution to publish OA or within certain

high-impact journals, some of which charge APCs, this creates layered tension and frustration. Authors might feel obligated to pay fees to advance their careers, even if they might not agree on moral grounds or if it limits their decision-making. The following response demonstrates this quandary:

> *Ability to pay fluctuates greatly depending on grant funding. But so long as the "prestige" associated with these journals is a key factor in evaluating faculty members, its [sic] hard not to [publish] there.* (#82, Pitt)

## The messy knot of the APC Trap

Our analysis and the examples provided show that the APC Trap is not a simple or straightforward phenomenon. The extent of its impact and the level at which any one researcher might experience any number of APC Trap elements is highly dependent on an individual's positionality. This includes their positionality within their discipline, within their institution, as well as their perceived positionality within a global and national context. There are likely many other points of positionality which could influence a researcher's experience of the APC Trap, such as being tenured or non-tenured, which were not variables in this study.

In other words, one researcher's experience of the APC Trap may look entirely different from another, depending on their positionality and their unique constraints and privileges. The key, unifying feature of the APC Trap is its resulting dissonance or "bind" – a frustration and acute sense of injustices within a system coupled with a need, obligation, or sense of moral responsibility to participate in such a system. This "bind" creates a sense of powerlessness, which only serves to pull the knot tighter.

# Limitations

The most prominent limitation of this study is that the survey instrument was not intentionally designed to facilitate a robust qualitative analysis. The initial aim of the study was to identify funding sources for APCs and perception of authors' ability to pay related to peers and to authors at less advantaged institutions, and the open-ended questions at the end of the survey were intended to capture additional thoughts regarding authors' ability to pay these fees to supplement the quantitative analysis. However, once the survey was completed, it was clear that many of the 48% of respondents who provided usable open-ended responses were expressing frustrations and anxiety about their experiences with OA publishing which went far beyond the original scope of the study. In other words, the survey was not designed from the outset to measure the APC Trap. Future research might find ways of better targeting and measuring the experiences which we observed obliquely.

Additionally, while many respondents used the open-ended questions to suit their own purposes, the framing of the questions themselves undoubtedly led many survey participants to respond in a particular manner. The two open-ended questions asked were:

> *Do you have any additional comments regarding authors' ability to pay publishing fees for open access?*
>
> and
>
> *Do you have any additional comments about the topics in this survey?*

The first question asks respondents specifically to speak to financial concerns related to APCs, and the second question also favors commentary about payment and cost because those were the topics of the previous multiple-choice questions in the survey. Questions targeting other aspects of OA publishing likely would have revealed additional APC Trap elements not captured by this study, and may have impacted the degree to which financial concern and other themes

related to finances (*Grants Don't Cut It*, *Threshold of Reasonableness*) were observed in the analysis. As noted previously, this concern about leading questions is also the reason we opted to exclude *Presupposition of Cost* from the final analysis.

Lastly, this study was designed to capture the sentiments of authors at very research-intensive (R1) institutions based in the ~~United States~~U.S., and so its implications for smaller and less well-resourced institutions, as well as global contexts, are limited. However, some limited, broader implications of the study are detailed in the following section.

## Conclusion and Broader Implications

Since APCs started gaining traction as a financial model for OA publishing at the beginning of the 20th century, scholars, librarians, authors, and some publishers have raised serious concerns about the long-term sustainability of the model. There have also been concerns about how APCs re-entrench uneven and inequitable participation in knowledge sharing and distribution.

This study confirms these concerns in ~~phenomenon appears in~~ the U.S. but may have global implications which could be explored in future studies. These concerns manifest in the privileged researchers of this study in the form of a phenomenon we coin the APC Trap and are characterized by eight overlapping and interacting characteristics. The eight elements of the APC Trap identified in this study mean that its actualization is unique from one researcher to the next. The limited scope of this study to four R1 institutions in the U.S. also means that there are undoubtedly valences of the APC Trap which are not captured in this study. However, our findings demonstrate how this phenomenon surfaces in the lived experiences of this niche set of researchers.

There are broader implications to be drawn from this study. First, whether the APC Trap is observed in wider, global contexts or not, its pervasiveness even within this relatively small

sample suggests the APC funding model faces many challenges to adoption and acceptance in the U.S. even when a strong ethical and ideological drive to publish open access is present. Second, if privileged researchers from some of the most well-resourced universities in the United States have such an agonized viewpoint of APCs, it accentuates the pain and hardship this model presents in other regions and socioeconomic contexts.

It is true that APCs are not the only model of sustainability for open access publishing, and a variety of alternative models have sought to repair concerns around cost and equity.

~~These concerns, coupled with innovations in publishing, have spawned a variety of alternative funding models for OA.~~ Plan S attempted to propose an APC cap as a form of price control. Transformative agreements were proposed as a way to ~~bridge from~~bridge subscriptions to OA publishing models, and as a way to formalize libraries' support of OA publishing on behalf of authors. SCOAP3 created a collective governance model of over 3,000 participating institutions to shift cost barriers away from individual authors. Subscribe to Open (S2O) models rely on reaching a threshold number of subscribing institutions to unlock research for everyone. While there are too many models to enumerate in full here, what is important to note is that the vast majority of proposed alternatives do not substantially reduce or offset the costs of OA publishing, but merely obscure the costs of publishing from authors, hiding them behind a new (but not altogether different) administrative veneer. We would go so far as to suggest that many of these alternative models do not solve the APC Trap, but merely redirect its impact onto other academic and administrative bodies, including librarians.

For all their faults, APCs – as opposed to the alternative approaches above - render the costs of OA publishing visible and palpable to authors. Through APCs we can directly observe the cognitive dissonance caused by the current scholarly publishing landscape:

> *"[T]he system is obviously bonkers, in that we create the products, review others' products for free, and then pay to get the products published. What a world!"* (#165, UCB)

> *"The whole system is a cash cow, and we pay and pay and pay. And worse, it's literally a requirement for our academic success. And that is before you investigate equity concerns…"* (#254, UMass)

> *"It feels like legalized extortion. I am a captive audience. I need to publish my work both because that is how I get promoted and remain competitive for grant funding. . . It just feels wrong."* (#52, Pitt)

To alleviate the experience of the APC Trap for authors, it may seem like an obvious solution to subsume those costs into new subscriptions, transformative agreements, and collective funding initiatives. While these models may temporarily avert the APC Trap insofar as they obscure the costs of publishing from authors, we should not assume they solve the problem of the APC Trap. Rather, they may simply be delaying the reckoning and entrenching the inequities by redirecting the ethical and financial burdens. When we must finally confront the trap, will we be bound too tight to break free?


# Acknowledgements

We thank Jessica Pearlman, Director of Research Methods Programs at University of Massachusetts Amherst's Institute for Social Science Research, for assistance with data analysis.

# References


1. Beasley G. Article processing charges: a new route to open access? Inf Serv Use. 2016; 36(3-4): 163-170. doi: ~~https://doi.org/10.3233/ISU-160815~~https://doi.org/10.3233/ISU-160815.

2. Khoo SYS. Article processing charge hyperinflation and price insensitivity: an open access sequel to the serials crisis. LIBER Q. 2019 May 9;29(1).doi:10.18352/lq.10280.

3. Klebel T, Ross-Hellauer T. The APC-Barrier and its effect on stratification in open access publishing. Quant Sci Stud. 2023; 4(1): 22-43. https://doi.org/10.1162/qss_a_00245.

4. Morrison HG. MDPI pricing. [Internet]. 2018 [cited 2019 April]. Available from: https://web.archive.org/web/20180817052832/https://sustainingknowledgecommons.org/2018/05/16/mdpi-pricing-thanks-to-mdpi-ceo-franck-vazquez-phd/.

5. Jain VK, Iyengar KP, Vaishya R. Article processing charges may be a barrier to publishing. J Clin Orthop Trauma. 2021;14: 14-16. https://doi.org/10.1016/j.jcot.2020.10.039

6. Maggio LA, Chtena N, Alperin JP, Moorhead L, Willinsky JM. (2024). “The best home for this paper”: A qualitative study of how authors select where to submit manuscripts. Perspect Med Educ. 2024 Sep 9;13(1). doi:10.5334/pme.1517

7. Warlick SE, Vaughan K. Factors influencing publication choice: why faculty choose open access. Biomed Digit Libr. 2007 Mar 9;4(1). doi:10.1186/1742-5581-4-1.

8. Demeter M, Istratii R. Scrutinising what open access journals mean for global inequalities. Pub Res Q. 2020;36: 505–522. https://doi.org/10.1007/s12109-020-09771-9

9. Borchardt R, Schultz T, Dawson D. Financial and other perceived barriers to transitioning to an equitable no-publishing fee open access model: A survey of LIS journal editors. Coll Res Libr. 2024; 85(1): 96. https://doi.org/10.5860/crl.85.1.96

10. Cantrell MH, Mezick J, Estill M, Caldwell R, Collister LB. An APC Trap? Privilege and the perception of reasonableness in open access publishing. J Libr Sch Commun. 2025; 13(1): eP18184. https://doi.org/10.31274/jlsc.18184

11. Dimensions. Definitions of Publications data fields in Dimensions [Internet] 2025. [cited 2025 October]. Available from: https://dimensions.freshdesk.com/support/solutions/articles/23000018864-definitions-of-publications-data-fields-in-dimensions-details-page-and-export-

12. Crawford W. Gold open access 2013-2018: Articles in journals (GOA4). [Internet]. Cities & Insights Books. 2019. Available from: https://waltcrawford.name/goa4.pdf

13. Cantrell, MH, Collister, LB. The status quo bias and the uptake of open access. First Monday. 2019; 24(7). https://doi.org/10.5210/fm.v24i7.10089

14. Pieper D. et al. OpenAPC data [Internet] 2025. [cited 2025 July]. Available from: https://treemaps.openapc.net/apcdata/openapc/.

15. Thelwall M, Buckley K, Paltoglou G. Sentiment strength detection for the social web. J Assoc Inf Sci Technol. 2011 Oct 13;61(12). doi:10.1002/asi.21662.

Melissa H. Cantrell
Scholarly Communication Librarian
Associate Professor
University of Colorado Boulder
melissa.cantrell@colorado.edu

Dear *PLOS One* Editors and Reviewers,

Thank you for the feedback and suggested revisions for our paper "'The System is Obviously Bonkers': The APC Trap and the Bind of Scholarly Publishing across Four R1 Institutions" for consideration in *PLOS One*.

Below you will find a table detailing each suggested revision as well as the action that was taken to address the revision. We would be happy to address any questions you may have about these revisions.

| **Issue Number** | **Reviewer/Editor** | **Issue Explanation** | **Completion Notes** |
|---|---|---|---|
| 1 | Editor | Please ensure that your manuscript meets PLOS ONE's style requirements, including those for file naming. The PLOS ONE style templates can be found at https://journals.plos.org/plosone/s/file?id=wjVg/PLOSOne_formatting_sample_main_body.pdf and https://journals.plos.org/plosone/s/file?id=ba62/PLOSOne_formatting_sample_title_authors_affiliations.pdf | Changed all references to figures in text to "Fig" as listed in instructions. Removed period after "Fig" in figure titles, bolded text, and moved title to be before figure as listed in instructions. Moved two figures so they appear after the text citations, as seems to be the preference per instructions. Confirmed that all headings are in appropriate font size. Bolded text for table titles and moved titles to be before table. Added space before table and figures as illustrated in doc. Also added the pilcrow symbol after all names, as this is how we are interpreting the instructions for signifying co-authorship. |
| 2 | Editor | Please include your full ethics statement in the 'Methods' section of your manuscript file. In your statement, please include the full name of the IRB or ethics committee who approved or waived your study, as well as whether or not you obtained informed written or verbal consent. If consent was waived for your study, please include this information in your statement as well. | Provided this at start of Methods section |

| | | | |
|---|---|---|---|
| 3 | Editor | We note you have included a table to which you do not refer in the text of your manuscript. Please ensure that you refer to Table 3 and 4 in your text; if accepted, production will need this reference to link the reader to the Table. | Corrected formatting; this is line 494 |
| 4 | Reviewer #1 | The introduction would benefit from a paragraph that introduces the topic to the more generalist reader – i.e. before getting into what this paper examines. In fact, this could be achieved by moving lines 39 to 50 to later in the introduction, and starting the manuscript with lines 51 onwards. | We have completely re-organized the introduction to provide context about the APC model, its origins, and its criticisms initially, ending the introduction with background about the previous study. |
| 5 | Reviewer #1 | Lines 81-88 should be removed from the introduction. This text covers results and discussion and so does not belong here. However, potentially lines 39-50 could be moved here to replace it | Addressed by introduction reorganization |
| 6 | Reviewer #1 | Line 91: citation missing | Added citation to our previous paper |
| 7 | Reviewer #1 | Table 2: The acronyms (HSS, NSE and HM) need to be explained in full | These acronyms are fully explained in Materials and Methods section. We wrote them out in full in this table as well. |
| 8 | Reviewer #1 | Table 3: The acronyms (e.g. UCB) need to be explained in full | These are explicated in materials and methods, but we have added them to the table |
| 9 | Reviewer #1 | Table 4: The acronym (OA) needs to be explained in full | Acronym spelled out in the table. |
| 10 | Reviewer #1 | Lines 717-723: A comment seems to have been left here. Remove. | Comment removed |
| 11 | Reviewer #1 | Limitations: I feel like the limitations need to be expanded. This is a relatively small sample from a very limited number of institutions. How generalisable are the findings? More needs to be said about this to make the paper more well rounded. | Provided a more explicit statement about limitations of global implications at the end of the Limitations section |
| 12 | Reviewer #2 | Abstract – redraft the abstract so that it makes sense on its own. It currently uses abbreviations and assumes implicit information about the US research landscape. | Significant edits to the abstract to make it more approachable to an international audience and to avoid using acronyms. |

| | | | |
|---|---|---|---|
| 13 | Reviewer #2 | Although this paper does not coin the term ‘APC Trap’, it draws on and elaborates on the idea – one which could become very influential. I think there is more potential to explain how this idea is more important across global academia and having tested the approach in a select group of US universities, these ideas should be expanded to consider global academic publshing. The paper arrives at this in the end. | While this paper does in fact coin the term "APC Trap," we have provided significant revisions throughout to situate this concept and its significance in a global context |
| 14 | Reviewer #2 | The questionnaire survey strategy focuses on 4 US research-intensive institutions and so the population from which the participants are drawn are more likely to have access to funds to cover the cost of APCs. The implications of this evidence base could be more clearly articulated. | Addressed in lines 112-113 (line numbers might change slightly) |
| 15 | Reviewer #2 | I suggest that the authors’ late argument about the motivation for the publication of this dataset in a separate paper (lines 754-762) should be brought more to a much earlier part of the manuscript to strengthen the introduction. | We could not identify the section of the paper the reviewer was referring to. We are happy to address this issue if further details can be provided. |
| 16 | Reviewer #2 | Avoid using ‘R1’ until it is explained for an international audience. Certainly do not use this terminology in the title or the abstract. Plos ONE is an international journal, so this needs to be made explicitly clear at the outset and I recommend the authors reflect on the wider global implications of what they have found in the specific context of US R1 institutions and their authors not just for other US-based researchers, but for researchers more widely. The manuscript does refer to the wider context later, so this is a relatively simple edit on the early part of the manuscript to make clear the scope of the paper. It needs to elaborate on the global implications of a study that focuses on a particular category of researcher that seems to | Used "research intensive institutions in the US" |

| | | | |
|---|---|---|---|
| | | be in a privileged position. | |
| 17 | Reviewer #2 | The manuscript does refer to the wider context later, so this is a relatively simple edit on the early part of the manuscript to make clear the scope of the paper. It needs to elaborate on the global implications of a study that focuses on a particular category of researcher that seems to be in a privileged position. | Added reference to impact globally on line 50 (lines may change slightly) |
| 18 | Reviewer #2 | p.2-3 I recommend a general contextual statement about the OA publishing model trends and the importance of APCs to that, rather than a previous publication the authors have written. This elevates the findings of the paper to addressing issues wider than a continuation of a previous paper published elsewhere, which seems to be how the first paragraph positions the paper and undersells its significance | This has been addressed in the re-write of the introduction. |

| | | | |
|---|---|---|---|
| 19 | Reviewer #2 | The paper should stand on its own terms, although I do understand that in some disciplines and publications this is less important where the methods are less contentious and more standardised. However, as I understand it PLOS One encourages more transparency, and this paper is in a branch of the research on publication that does traditionally include transparent discussion and justification of methods. For guidance, I refer the authors to the PLOS One policy on statistical reporting in the Submission Guidelines in which this useful general principle is worth considering: "The Materials and Methods section should provide enough detail to allow suitably skilled investigators to fully replicate your study." This does not have to be detailed, but basic explanation of the recruitment process, the demographic background of the respondents. Note that your methods section has only 2 citations, both of which are for your own previous paper which raises a question about how well embedded in existing research your methods are. | Line 147-154: added citations to other studies with similar methods |
| 20 | Reviewer #2 | p.8 This manuscript does not report the total number of cases (ie academic researchers) included in the dataset. The manuscript mentions 136 responses (p.8, line 170) and 321 responses of which 154 'had a usable response' (p.18, line 428), but I was unclear if this meant 136 researchers or 136 answers to the two questions. | 18 responses did not contain enough content to do a sentiment analysis - included note of this |

| | | | |
|---|---|---|---|
| 21 | Reviewer #2 | P.7 Sentiment analysis. This whole section is weakened at the outset with the statement to the effect that the authors did not expect it to yield much. The rest of the section describes how this function in a piece of software lived up to those low expectations. Although there is some reporting of the outcome, the findings from this is an insufficient basis to merit the attention it is given. Although the authors explain the criticisms of autocoding, they do not provide any sources for this. I recommend this section is removed from the manuscript and a summarised account of the useful findings are incorporated as a minor element of the analysis of the evidence. | Added this as a short section above the "APC Trap based on responses to other survey questions" section, highlighting how the interaction of responses can provide some indicator of sentiment that the sentiment analysis tools failed to provide. |
| 22 | Reviewer #2 | p.15 The section entitled Code Book, seems to actually be the findings or results section. This presents interesting selection of the responses gathered for this research. However, the discussion section presents the most important contribution. This is the discussion of the codes, though the paper is not as clear as it could be how these codes are arrived at. The discussion mentions discussion amongst the research team and conclusions. The manuscript provides no contextual description of the four institutions, so it is unclear why outcomes were interesting. | New title of section so the codebook is now the "Results" |

| | | | |
|---|---|---|---|
| 23 | Reviewer #2 | I therefore recommend that the manuscript provides:<br>1. A brief description of each of the four institutions and how they are positions as research institutions in the US. A revision should take account of an international audience that does not have the implicit knowledge of the research landscape and that explains the wider significance. - Jennie doing this<br>2. An explanation of how the authors view the focus on these four institutions as providing useful evidence that enables wider interpretations to be drawn.<br>3. A stronger justification of the rationale for writing this paper that draws on wider arguments.<br>4. A re-drafted methods section that clarifies the methods used and explains and justifies how this limited and purposive sample provide a basis for the manuscript to make broader interpretations. | This is addressed throughout by other comments; #4 is addressed in Methods lines 159-166 |
| 24 | Reviewer #2 | p.5 You refer to categorisation of publications based on 'reporting in Dimensions' without explaining this. A citation and brief explanation is required to clarify this for an international audience. | Explained what Dimensions was and provided citation to publications categories. Also shifted numbering for remaining citations |
| 25 | Reviewer #2 | p.11 The institutions in the survey are only mentioned in the introduction. However, there are some useful insights about the differences in the responses between academics from the institutions. It may be useful to have a brief description of each institution (even if just in a table) from the context of their research identity. There appear to be points being made the required implicit knowledge or unstated assumptions about these institutions that may be obvious to a US academic that could be made explicit. | Summarized the institions briefly in the first paragraph of Materials and Methods. Tried to make a table that lists 2021-2022 student FTE, employee FTE, research expenditures, and research income from grants. However, not every institution has a public factbook for this historical data and the data would take work to pull out of IPEDS. For example, UTK data includes the medical school yet we excluded the medical school from the survey. |

| | | | |
|---|---|---|---|
| 26 | Reviewer #2 | P. 13 & multiple – you have used abbreviations such as NIH (p. 13, 33, 34) and NSF (p.13). These should be in-full when first used. | Explication provided in brackets at first mention |
| 27 | Reviewer #2 | p. 36 The conclusion brings into the discussion Plan S, SCOAP3 and S20 none of which have been discussed in the main part of the paper – nor were any of the three direct quotes from participants. I suggest these elements of the conclusion be moved o the discussion and the conclusion then rests only on the contents of the paper. | We don't think this belongs in the Discussion because, as the reviewer states, these initiatives are not brought up by participants, but we renamed the conclusion to include broader implications. Also added a paragraph in the conclusion tying the findings and discussion into the borader context of OA publishing. Added section 883-900 |

Sincerely,

Melissa H. Cantrell (University of Colorado Boulder co-PI), Rachel Caldwell (University of Massachusetts Amherst co-PI), Jennifer Mezick (University of Tennessee Knoxville co-PI), Matthew Estill (University of Pittsburgh co-PI), and Lauren B. Collister (Invest in Open Infrastructure, co-PI and advisor)
