# Supplementary material for: “The system is obviously bonkers”: The APC Trap and the bind of scholarly publishing across four research intensive institutions in the U.S
Source: PLoS One. 2026 Jul 1;21(7):e0351430. doi: 10.1371/journal.pone.0351430 (PMC13322547; doi:10.1371/journal.pone.0351430)
Supplement: Attachment — Submitted filename: Round2_PLOSOne_ResponsetoReviewers.pdf [file pone.0351430.s006.pdf]

Melissa H. Cantrell
Scholarly Communication Librarian
Associate Professor
University of Colorado Boulder
melissa.cantrell@colorado.edu

Dear *PLOS One* Editors and Reviewers,

Thank you for the additional feedback and suggested revisions for our paper "'The System is Obviously Bonkers': The APC Trap and the Bind of Scholarly Publishing across Four Research Intensive Institutions in the U.S." for consideration in *PLOS One*.

We conducted significant revisions for this second round of review, including a complete reorganization and partial re-write of the Results and Discussion sections; moving the sub-section on sentiment analysis to the Limitations section; and incorporating several new citations to back up claims made in the paper.

Below you will find a table detailing each suggested revision as well as the action that was taken to address the revision. We would be happy to address any questions you may have about these revisions.

Issue Number	Section	Issue Explanation	Completion Notes
1	Abstract	Line 26 – you use the acronym US before introducing the full spelling.	Spelled out United States in the abstract
2	Introduction	• Beyond your own previous study, how does your study build or compliment existing studies that survey author behaviour as it relates to APCs, specifically in the US? Is there anything of use in the following study, for example: Olejniczak, A. J., & Wilson, M. J. (2020). Who's writing open access (OA) articles? Characteristics of OA authors at Ph.D.-granting institutions in the United States. Quantitative Science Studies, 1(4), 1429–1450. https://doi.org/10.1162/qss_a_00091.<br>o You could cite this study somewhere around lines 54-56, providing supporting evidence of attitudes and trends of the demographic makeup of who pays APCs in the US.<br>o Or perhaps even around lines 77 or 78, further strengthening the argument not only of cognitive dissonance, but the	Added a mention of a study of US authors that found a correlation between APC OA and privilege, gender, career level, and prestige of uni. Added citation to this article: Olejniczak, A. J., & Wilson, M. J. (2020). Who's writing open access (OA) articles? Characteristics of OA authors at Ph.D.-granting institutions in the United States. Quantitative Science Studies, 1(4), 1429–1450. https://doi.org/10.1162/qss_a_00091.

		socio-cultural impacts the influence author behaviour (e.g., gender).	
3	Introduction	• Lines 56 – 57: You argue that pricing APCs is based on prestige rather than production costs, which is an important argument. Before citing the literature that looks at prestige and hyperinflation, you may want to consider citing studies that examine this particular pricing strategy (prestige), then cite the effects of hyperinflation. For example,<br>o Demeter, M., & Istratii, R. (2020). Scrutinising what Open Access Journals Mean for Global Inequalities. Publishing Research Quarterly, 36(4), 505–522. https://doi.org/10.1007/s12109-020-09771-9<br>o Schönfelder, N. (2020). Article processing charges: Mirroring the citation impact or legacy of the subscription-based model? Quantitative Science Studies, 1(1), 6–27. https://doi.org/10.1162/qss_a_00015	Added citation to Schönfelder, N. (2020). Article processing charges: Mirroring the citation impact or legacy of the subscription-based model? Quantitative Science Studies, 1(1), 6–27. https://doi.org/10.1162/qss_a_00015
4	Introduction	· In your methods, you state that you group the surveys by three disciplines. This is a particularly important approach since disciplinary practices are so unique, and studies have shown APC pricing can differ across disciplines. You may want to consider citing this factor, you do not necessarily need to be exhaustive but pointing out to a relevant study or two can further strengthen your methodological choice.	Added a mention in line 45, still references reference 3 (Klebel & Ross-Hellauer)
5	Introduction	· Line 62: starting with “Journal editors…” reads as though ALL journal editors share this view, but I would caution not to generalize that statement as editorship functions differently across journals.	Revised to indicate the editors who responded to this particular survey referenced at the end of the sentence.

6	Materials and Methods	· I think a bit of restructuring in the methods section, with clearer subheadings, could aid with readability. A good example of a study using surveys with nice subsections: https://doi.org/10.1371/journal.pone.0301251 and https://doi.org/10.1162/qss_a_00264 . For example, in line 171 of your paper: “While there were 321 completed surveys, 154 of those also included usable…”, is really about response rates, which you could expand upon. You can move this sentence to a new section (something like Survey Responses, or Response Rates), and you can expand upon the response rate to include details like the rate of completion for the two open-ended questions, or perhaps whether any respondents in the quantitative part of the survey dropped out and did not respond to the open-ended questions? Did you receive more responses for the gold than hybrid (or vice versa) surveys, or did respondents evenly complete both? What about responses by field – are respondents proportionally represented across the disciplines, or did you receive more responses from a certain field? Table 1 could be moved here as well, since the table does not really expand on the discussion, but rather visualizes a result.	subheadings and response rates added. Decided this worked better under Results
7	Materials and Methods	· Lines 125 – 127: expand upon the funding profile of these institutions (and my apologies if this is convered elsewhere) – are researchers typically well-funded? In your intro, you argue about under-resourced institutions and certain geographic areas being disadvantaged by the APC trap; did you look at this variable in your study? If yes, this should be made clearer in the methods section.	Updated - citation added
8	Materials and Methods	· Can you expand a bit on your coding method? It was done inductively,	Updated to further explain coding method

		correct? Lines 193-195 hint at this (through discussion).	
9	Materials and Methods	*· Line 199: you could add a bit of detail on why you chose not to analyze the additional theme Presupposition of Cost.*	Added more detail for rationale of not including this theme
10	Materials and Methods	· Lines 199 – 202: "Respondents also conveyed broader sentiments, including frustration…" reads more like a result than a method, but follows your description of coding methods. Suggest moving this statement to your results section, and instead discuss here how you coded for the themes of frustration and appreciation.	Removed sentence - we did not code for frustration or appreciation so it is not significant to the overall analysis or purpose of the paper.
11	Materials and Methods	• Lines 658 -682 (Section on Sentiment Analysis) –<br>o Reviewer 2 suggests a citation, and I still do not see one added. I agree this is important to support your critiques of this method. You state in line 677 that these tools "often fail to grasp…"; the word often implies knowledge of this challenge elsewhere. I am curious if other studies have also experience similar challenges? This would be helpful information for future studies aiming to use this method.<br>o Additionally, this section seems awkward here. Since you mention, in line 676, how the results highlight a limitation, is this better in the limitation section?	Moved sentiment analysis to limitations and added citation supporting our claims about its limitations.
12	Results	· I understand Reviewer 2 suggested the section previously titled "Code book" seems more like findings. I would still suggest you nevertheless provide a brief overview in your methods section on the 8 categories themselves, perhaps after you introduce your approach to manual coding.	Chose not to implement this for two reasons: 1) It seemed duplicative, and 2) introducing the codes along with the approach to manual coding makes it seem like the codes informed the coding, which was not the case. Rather, the codes were a result that arose from the iterative process of manual coding.

13	Results	In your results section, you could provide a table that includes the 8 codes and the number of coded responses per category, to visualize how influential the codes are on author behaviour. Then this could be further unpacked in the discussion section – not surprising that your code “financial concerns” is the most frequent, and interesting that 30% of the results include aspects of the code Diversity of equity.	See comment below on the same issue - this table was moved up to the results section
14	Results	Your section on “Codes” can be framed in a way that emphasizes the results – see comment on page 61, lines 272 in PDF. What about the specific responses influenced our decision to showcase?	Addressed why specific quotes were included in the code book as "exemplary"
15	Results	· See comment in PDF, line 303 – follow same format for results (n=46; 28%) as the former sections.	Addressed this formatting throughout
16	Results	· Line 420 – Presupposition of cost – is this a 9th code? I’m confused, since you specified 8. Why is this not considered the 9th if you coded responses according to this categorical topic? See my comment in PDF, around line 429-430.	Clarified and emphasized this point
17	Results	· Line 471 – this argument is unclear to me – I am not grasping what you mean by “a certain instance of that model is to blame”. What do you mean by “instance”?	Clarified that "instance" means a specific journal or publisher, rather than the model of publishing itself
18	Discussion	I am struggling with this section as much of the detail reads more like results than a discussion. I suggest revisiting and moving any findings up to your results section and/or cutting any duplication	Findings were all moved up to results and duplication was addressed
19	Discussion	· The Discussion section should stick to exploring your findings (not restating them) - what factors are influencing the behaviour - why do you think so many respondents touched on diversity and equity themes? What is the impact of this for institutions and, more	Completely re-worked the discussion section to focus on these aspects and more

		importantly, R1 institutions who are well-resourced? Etc…	
20	Discussion	· Line 488-492 is repeated information already detailed in your methods section, although you can move up the part about “usable” if not discussed earlier.	Removed duplication
21	Discussion	· Lines 500-502 could serve as the start of this section, since it reads more like a summary of findings.	Moved these lines up to the top of the Results section
22	Discussion	· Line 514 – what are APC elements? This is a new concept being introduced. Are “elements” the “codes”? If yes, please clarify in the manuscript. If they differ from codes, please better define elements. Even more, as a reader, I am left struggling to understand why the results from this analysis are in the Discussion section, while the results from the codes are in the Results section?	Revised the document so that "codes" is used consistently throughout, to avoid confusion
23	Discussion	· In my comments under the Results section, I suggest tabling the number of responses by code, so it is great that you chose to include a figure visualizing the elements (although again, how does this differ from codes?). However, this figure could move to your Results section, if you choose to expand upon response rates as suggested.	Table has been moved to results section under "APC Trap overview"
24	Discussion	· Line 542 – 547 – this is the type of information I would expect to read in a discussion section. What about the other codes? Can you point to other literature that supports or extends possibilities for your findings?	Moved information like this to the discussion and expanded
25	Discussion	· Line 549 - 555 and Tables 2 & 3 belongs more in Results section, can even be situated in a section on Survey Responses, providing valuable information on the demographics of respondents.	Moved to Results

26	Discussion	· Much of the information in Lines 582 until the end of discussion belong in the Results section. I suggest the authors review this section again, pull up any data to Results, and then summarize key points in your Discussion, and make claims about the results themselves, citing relevant studies where relevant that may support your claims, or where you are extending ideas from other studies. Section – “The messy knot of the APC Trap” – line 842 provides a nice example where you do discuss the findings.	All moved to the results section
27	Discussion	· While I understand you used your 2022 dataset, Transformative Agreements / Read and Publish agreement have continued to increase in popularity. The fact that these often include full waivers for hybrid journals, over gold, make me wonder if they are influencing author behaviour, whereby we see authors selecting journals based on the 100% discount. How does this influence your results? Is it possible that a new type of “trap” includes authors preferring prestigious journals with full waivers owing to their institutions TAs? Do the institutions you study have TAs in place? Particular relevant since your initial dataset cleans the data by the corresponding author field.	Addressed this in the the Conclusion
28	Limitations	· Lines 887 – 888 – “and so its implications for smaller and less well-resources institutions…are limited”. What exactly do you mean by implications, here? Whether the results can provide any meaning for smaller institutions? I would argue that because you used purposive sampling, your results cannot be generalized beyond your dataset, so you may want to tweak this sentence a bit.	Added: "As a study with a purposive sample, findings are not representative..."

29	Conclusion	Line 896 – I would suggest softening the language in line 896, specifically the use of the word “confirm”. Can the study confirm the concerns in the U.S.? You used purposive sampling, so your results can only be generalized to the respondents in your dataset. You state in your limitations that your study focused on authors are very research-intensive institutions, so its implications for smaller and less-resourced institutions are limited. You could instead allude that the results support other findings regarding inequities, and then cite a few studies.	Removed "confirmed" and replaced with "demonstrates". Reworded some of the global implications paragraph to soften the language. Reframed the beginning of that paragraph with "raises questions" where it did state that the study draws broader implications.
30	Conclusion	Line 901-902 – the limited scope is also that you used purposive sampling, and perhaps there are authors outside the dataset with other tendencies and behaviours.	Included the sampling method and reworded to directly state experiences by authors outside this study.
31	Throughout	A bit more work is needed to streamline and make the writing more concise	Removed all "we" and "us" references in our text of the paper (not quotes) with exception to the last sentence of the conclusion, which WE is appropriate as its all researchers. Also read through the entire thing and made lots of suggestions to make parts more understandable or more concise.

Sincerely,

Melissa H. Cantrell (University of Colorado Boulder co-PI), Rachel Caldwell (University of Massachusetts Amherst co-PI), Jennifer Mezick (University of Tennessee Knoxville co-PI), Matthew Estill (University of Pittsburgh co-PI), and Lauren B. Collister (Invest in Open Infrastructure, co-PI and advisor)
