# Supplementary material for: “The system is obviously bonkers”: The APC Trap and the bind of scholarly publishing across four research intensive institutions in the U.S
Source: PLoS One. 2026 Jul 1;21(7):e0351430. doi: 10.1371/journal.pone.0351430 (PMC13322547; doi:10.1371/journal.pone.0351430)
Supplement: Attachment — Submitted filename: Review Report of PONE-D-25-47145.docx [file pone.0351430.s001.docx]

# Review Report of PONE-D-25-47145

- This is a highly topical paper that focuses on an area of publication in science and social science disciplines that is undergoing considerable change but with mixed views about the impacts and implications. This paper offers some evidence that provides some insight into the topic.
- The writing is clear and focused.
- The evidence base is substantial drawing on open text responses to an online questionnaire survey of a moderate size

## Major issues

- Abstract – redraft the abstract so that it makes sense on its own. It currently uses abbreviations and assumes implicit information about the US research landscape.
- Although this paper does not coin the term ‘APC Trap’, it draws on and elaborates on the idea – one which could become very influential. I think there is more potential to explain how this idea is more important across global academia and having tested the approach in a select group of US universities, these ideas should be expanded to consider global academic publshing. The paper arrives at this in the end.
- The questionnaire survey strategy focuses on 4 US research-intensive institutions and so the population from which the participants are drawn are more likely to have access to funds to cover the cost of APCs. The implications of this evidence base could be more clearly articulated.
- The article makes the reasonable argument that if these relatively privileged academic respondents in an affluent society provide evidence of their concerns and criticism of this model, then there is something to be further analysed.
- I suggest that the authors’ late argument about the motivation for the publication of this dataset in a separate paper (lines 754-762) should be brought more to a much earlier part of the manuscript to strengthen the introduction.

Avoid using ‘R1’ until it is explained for an international audience. Certainly do not use this terminology in the title or the abstract. *Plos ONE* is an international journal, so this needs to be made explicitly clear at the outset and I recommend the authors reflect on the wider global implications of what they have found in the specific context of US R1 institutions and their authors not just for other US-based researchers, but for researchers more widely. The manuscript does refer to the wider context later, so this is a relatively simple edit on the early part of the manuscript to make clear the scope of the paper. It needs to elaborate on the global implications of a study that focuses on a particular category of researcher that seems to be in a privileged position.

p.2-3 I recommend a general contextual statement about the OA publishing model trends and the importance of APCs to that, rather than a previous publication the authors have written. This elevates the findings of the paper to addressing issues wider than a continuation of a previous paper published elsewhere, which seems to be how the first paragraph positions the paper and undersells its significance.

The paper should stand on its own terms, although I do understand that in some disciplines and publications this is less important where the methods are less contentious and more standardised. However, as I understand it *PLOS One* encourages more transparency, and this paper is in a branch of the research on publication that does traditionally include transparent discussion and justification of methods. For guidance, I refer the authors to the *PLOS One* policy on statistical reporting in the Submission Guidelines in which this useful general principle is worth considering: “The Materials and Methods section should provide enough detail to allow suitably skilled investigators to fully replicate your study.” This does not have to be detailed, but basic explanation of the recruitment process, the demographic background of the respondents. Note that your methods section has only 2 citations, both of which are for your own previous paper which raises a question about how well embedded in existing research your methods are.

p.8 This manuscript does not report the total number of cases (ie academic researchers) included in the dataset. The manuscript mentions 136 responses (p.8, line 170) and 321 responses of which 154 ‘had a usable response’ (p.18, line 428), but I was unclear if this meant 136 researchers or 136 answers to the two questions.

P.7 Sentiment analysis. This whole section is weakened at the outset with the statement to the effect that the authors did not expect it to yield much. The rest of the section describes how this function in a piece of software lived up to those low expectations. Although there is some reporting of the outcome, the findings from this is an insufficient basis to merit the attention it is given. Although the authors explain the criticisms of autocoding, they do not provide any sources for this. **I recommend this section is removed** from the manuscript and a summarised account of the useful findings are incorporated as a minor element of the analysis of the evidence.

p.15 The section entitled **Code Book**, seems to actually be the findings or results section. This presents interesting selection of the responses gathered for this research. However, the discussion section presents the most important contribution. This is the discussion of the codes, though the paper is not as clear as it could be how these codes are arrived at. The discussion mentions discussion amongst the research team and conclusions. The manuscript provides no contextual description of the four institutions, so it is unclear why outcomes were interesting.

**I therefore recommend that the manuscript provides**:

1. A brief description of each of the four institutions and how they are positions as research institutions in the US. A revision should take account of an international audience that does not have the implicit knowledge of the research landscape and that explains the wider significance.
2. An explanation of how the authors view the focus on these four institutions as providing useful evidence that enables wider interpretations to be drawn.
3. A stronger justification of the rationale for writing this paper that draws on wider arguments.
4. A re-drafted methods section that clarifies the methods used and explains and justifies how this limited and purposive sample provide a basis for the manuscript to make broader interpretations.

## Minor revisions

p.5 You refer to categorisation of publications based on ‘reporting in Dimensions’ without explaining this. A citation and brief explanation is required to clarify this for an international audience.

p.11 The institutions in the survey are only mentioned in the introduction. However, there are some useful insights about the differences in the responses between academics from the institutions. It may be useful to have a brief description of each institution (even if just in a table) from the context of their research identity. There appear to be points being made the required implicit knowledge or unstated assumptions about these institutions that may be obvious to a US academic that could be made explicit.

P. 13 & multiple – you have used abbreviations such as NIH (p. 13, 33, 34) and NSF (p.13). These should be in-full when first used.

p. 36 The conclusion brings into the discussion Plan S, SCOAP3 and S20 none of which have been discussed in the main part of the paper – nor were any of the three direct quotes from participants. I suggest these elements of the conclusion be moved o the discussion and the conclusion then rests only on the contents of the paper.
