# Supplementary material for: “The system is obviously bonkers”: The APC Trap and the bind of scholarly publishing across four research intensive institutions in the U.S
Source: PLoS One. 2026 Jul 1;21(7):e0351430. doi: 10.1371/journal.pone.0351430 (PMC13322547; doi:10.1371/journal.pone.0351430)
Supplement: Attachment — Submitted filename: PLOS One Revisions Letter.docx [file pone.0351430.s005.docx]

Melissa H. Cantrell
Scholarly Communication Librarian
Associate Professor
University of Colorado Boulder	[melissa.cantrell@colorado.edu](mailto:melissa.cantrell@colorado.edu)

Dear *PLOS One* Editors and Reviewers,

Thank you for the feedback and suggested revisions for our paper “‘The System is Obviously Bonkers’: The APC Trap and the Bind of Scholarly Publishing across Four R1 Institutions" for consideration in *PLOS One*.

Below you will find a table detailing each suggested revision as well as the action that was taken to address the revision. We would be happy to address any questions you may have about these revisions.

| **Issue Number** | **Reviewer/Editor** | **Issue Explanation** | **Completion Notes** |
| --- | --- | --- | --- |
| 1 | Editor | Please ensure that your manuscript meets PLOS ONE's style requirements, including those for file naming. The PLOS ONE style templates can be found at  <https://journals.plos.org/plosone/s/file?id=wjVg/PLOSOne_formatting_sample_main_body.pdf> and  <https://journals.plos.org/plosone/s/file?id=ba62/PLOSOne_formatting_sample_title_authors_affiliations.pdf> | Changed all references to figures in text to "Fig" as listed in instructions. Removed period after "Fig" in figure titles, bolded text, and moved title to be before figure as listed in instructions. Moved two figures so they appear after the text citations, as seems to be the preference per instructions. Confirmed that all headings are in appropriate font size. Bolded text for table titles and moved titles to be before table. Added space before table and figures as illustrated in doc. Also added the pilcrow symbol after all names, as this is how we are interpreting the instructions for signifying co-authorship. |
| 2 | Editor | Please include your full ethics statement in the ‘Methods’ section of your manuscript file. In your statement, please include the full name of the IRB or ethics committee who approved or waived your study, as well as whether or not you obtained informed written or verbal consent. If consent was waived for your study, please include this information in your statement as well. | Provided this at start of Methods section |
| 3 | Editor | We note you have included a table to which you do not refer in the text of your manuscript. Please ensure that you refer to Table 3 and 4 in your text; if accepted, production will need this reference to link the reader to the Table. | Corrected formatting; this is line 494 |
| 4 | Reviewer #1 | The introduction would benefit from a paragraph that introduces the topic to the more generalist reader – i.e. before getting into what this paper examines. In fact, this could be achieved by moving lines 39 to 50 to later in the introduction, and starting the manuscript with lines 51 onwards. | We have completely re-organized the introduction to provide context about the APC model, its origins, and its criticisms initially, ending the introduction with background about the previous study. |
| 5 | Reviewer #1 | Lines 81-88 should be removed from the introduction. This text covers results and discussion and so does not belong here. However, potentially lines 39-50 could be moved here to replace it | Addressed by introduction reorganization |
| 6 | Reviewer #1 | Line 91: citation missing | Added citation to our previous paper |
| 7 | Reviewer #1 | Table 2: The acronyms (HSS, NSE and HM) need to be explained in full | These acronyms are fully explained in Materials and Methods section. We wrote them out in full in this table as well. |
| 8 | Reviewer #1 | Table 3: The acronyms (e.g. UCB) need to be explained in full | These are explicated in materials and methods, but we have added them to the table |
| 9 | Reviewer #1 | Table 4: The acronym (OA) needs to be explained in full | Acronym spelled out in the table. |
| 10 | Reviewer #1 | Lines 717-723: A comment seems to have been left here. Remove. | Comment removed |
| 11 | Reviewer #1 | Limitations: I feel like the limitations need to be expanded. This is a relatively small sample from a very limited number of institutions. How generalisable are the findings? More needs to be said about this to make the paper more well rounded. | Provided a more explicit statement about limitations of global implications at the end of the Limitations section |
| 12 | Reviewer #2 | Abstract – redraft the abstract so that it makes sense on its own. It currently uses abbreviations and assumes implicit information about the US research landscape. | Significant edits to the abstract to make it more approachable to an international audience and to avoid using acronyms. |
| 13 | Reviewer #2 | Although this paper does not coin the term ‘APC Trap’, it draws on and elaborates on the idea – one which could become very influential. I think there is more potential to explain how this idea is more important across global academia and having tested the approach in a select group of US universities, these ideas should be expanded to consider global academic publshing. The paper arrives at this in the end. | While this paper does in fact coin the term "APC Trap," we have provided significant revisions throughout to situate this concept and its significance in a global context |
| 14 | Reviewer #2 | The questionnaire survey strategy focuses on 4 US research-intensive institutions and so the population from which the participants are drawn are more likely to have access to funds to cover the cost of APCs. The implications of this evidence base could be more clearly articulated. | Addressed in lines 112-113 (line numbers might change slightly) |
| 15 | Reviewer #2 | I suggest that the authors’ late argument about the motivation for the publication of this dataset in a separate paper (lines 754-762) should be brought more to a much earlier part of the manuscript to strengthen the introduction. | We could not identify the section of the paper the reviewer was referring to. We are happy to address this issue if further details can be provided. |
| 16 | Reviewer #2 | Avoid using ‘R1’ until it is explained for an international audience. Certainly do not use this terminology in the title or the abstract. Plos ONE is an international journal, so this needs to be made explicitly clear at the outset and I recommend the authors reflect on the wider global implications of what they have found in the specific context of US R1 institutions and their authors not just for other US-based researchers, but for researchers more widely. The manuscript does refer to the wider context later, so this is a relatively simple edit on the early part of the manuscript to make clear the scope of the paper. It needs to elaborate on the global implications of a study that focuses on a particular category of researcher that seems to be in a privileged position. | Used "research intensive institutions in the US" |
| 17 | Reviewer #2 | The manuscript does refer to the wider context later, so this is a relatively simple edit on the early part of the manuscript to make clear the scope of the paper. It needs to elaborate on the global implications of a study that focuses on a particular category of researcher that seems to be in a privileged position. | Added reference to impact globally on line 50 (lines may change slightly) |
| 18 | Reviewer #2 | p.2-3 I recommend a general contextual statement about the OA publishing model trends and the importance of APCs to that, rather than a previous publication the authors have written. This elevates the findings of the paper to addressing issues wider than a continuation of a previous paper published elsewhere, which seems to be how the first paragraph positions the paper and undersells its significance | This has been addressed in the re-write of the introduction. |
| 19 | Reviewer #2 | The paper should stand on its own terms, although I do understand that in some disciplines and publications this is less important where the methods are less contentious and more standardised. However, as I understand it PLOS One encourages more transparency, and this paper is in a branch of the research on publication that does traditionally include transparent discussion and justification of methods. For guidance, I refer the authors to the PLOS One policy on statistical reporting in the Submission Guidelines in which this useful general principle is worth considering: “The Materials and Methods section should provide enough detail to allow suitably skilled investigators to fully replicate your study.” This does not have to be detailed, but basic explanation of the recruitment process, the demographic background of the respondents. Note that your methods section has only 2 citations, both of which are for your own previous paper which raises a question about how well embedded in existing research your methods are. | Line 147-154: added citations to other studies with similar methods |
| 20 | Reviewer #2 | p.8 This manuscript does not report the total number of cases (ie academic researchers) included in the dataset. The manuscript mentions 136 responses (p.8, line 170) and 321 responses of which 154 ‘had a usable response’ (p.18, line 428), but I was unclear if this meant 136 researchers or 136 answers to the two questions. | 18 responses did not contain enough content to do a sentiment analysis - included note of this |
| 21 | Reviewer #2 | P.7 Sentiment analysis. This whole section is weakened at the outset with the statement to the effect that the authors did not expect it to yield much. The rest of the section describes how this function in a piece of software lived up to those low expectations. Although there is some reporting of the outcome, the findings from this is an insufficient basis to merit the attention it is given. Although the authors explain the criticisms of autocoding, they do not provide any sources for this. I recommend this section is removed from the manuscript and a summarised account of the useful findings are incorporated as a minor element of the analysis of the evidence. | Added this as a short section above the "APC Trap based on responses to other survey questions" section, highlighting how the interaction of responses can provide some indicator of sentiment that the sentiment analysis tools failed to provide. |
| 22 | Reviewer #2 | p.15 The section entitled Code Book, seems to actually be the findings or results section. This presents interesting selection of the responses gathered for this research. However, the discussion section presents the most important contribution. This is the discussion of the codes, though the paper is not as clear as it could be how these codes are arrived at. The discussion mentions discussion amongst the research team and conclusions. The manuscript provides no contextual description of the four institutions, so it is unclear why outcomes were interesting. | New title of section so the codebook is now the "Results" |
| 23 | Reviewer #2 | I therefore recommend that the manuscript provides:  1. A brief description of each of the four institutions and how they are positions as research institutions in the US. A revision should take account of an international audience that does not have the implicit knowledge of the research landscape and that explains the wider significance. - Jennie doing this  2. An explanation of how the authors view the focus on these four institutions as providing useful evidence that enables wider interpretations to be drawn.  3. A stronger justification of the rationale for writing this paper that draws on wider arguments.  4. A re-drafted methods section that clarifies the methods used and explains and justifies how this limited and purposive sample provide a basis for the manuscript to make broader interpretations. | This is addressed throughout by other comments; #4 is addressed in Methods lines 159-166 |
| 24 | Reviewer #2 | p.5 You refer to categorisation of publications based on ‘reporting in Dimensions’ without explaining this. A citation and brief explanation is required to clarify this for an international audience. | Explained what Dimensions was and provided citation to publications categories. Also shifted numbering for remaining citations |
| 25 | Reviewer #2 | p.11 The institutions in the survey are only mentioned in the introduction. However, there are some useful insights about the differences in the responses between academics from the institutions. It may be useful to have a brief description of each institution (even if just in a table) from the context of their research identity. There appear to be points being made the required implicit knowledge or unstated assumptions about these institutions that may be obvious to a US academic that could be made explicit. | Summarized the institions briefly in the first paragraph of Materials and Methods. Tried to make a table that lists 2021-2022 student FTE, employee FTE, research expenditures, and research income from grants. However, not every institution has a public factbook for this historical data and the data would take work to pull out of IPEDS. For example, UTK data includes the medical school yet we excluded the medical school from the survey. |
| 26 | Reviewer #2 | P. 13 & multiple – you have used abbreviations such as NIH (p. 13, 33, 34) and NSF (p.13). These should be in-full when first used. | Explication provided in brackets at first mention |
| 27 | Reviewer #2 | p. 36 The conclusion brings into the discussion Plan S, SCOAP3 and S20 none of which have been discussed in the main part of the paper – nor were any of the three direct quotes from participants. I suggest these elements of the conclusion be moved o the discussion and the conclusion then rests only on the contents of the paper. | We don't think this belongs in the Discussion because, as the reviewer states, these initiatives are not brought up by participants, but we renamed the conclusion to include broader implications. Also added a paragraph in the conclusion tying the findings and discussion into the borader context of OA publishing. Added section 883-900 |

Sincerely,

Melissa H. Cantrell (University of Colorado Boulder co-PI), Rachel Caldwell (University of Massachusetts Amherst co-PI), Jennifer Mezick (University of Tennessee Knoxville co-PI), Matthew Estill (University of Pittsburgh co-PI), and Lauren B. Collister (Invest in Open Infrastructure, co-PI and advisor)
