# Supplementary material for: “The system is obviously bonkers”: The APC Trap and the bind of scholarly publishing across four research intensive institutions in the U.S
Source: PLoS One. 2026 Jul 1;21(7):e0351430. doi: 10.1371/journal.pone.0351430 (PMC13322547; doi:10.1371/journal.pone.0351430)
Supplement: Attachment — Submitted filename: Review_Cantrell et al_PLOS.docx [file pone.0351430.s004.docx]

Overall, this study provides relevant and timely findings that advance our understanding of the attitudes of authors in regard to APCs. However, I do find the paper could be better structured to ease the reader’s ability to understand the methods and findings, and a bit more finessing of language for accuracy. A bit more work is needed to streamline and make the writing more concise, and another read through to ensure acronyms are consistent (e.g., open access (OA)) throughout.

I am therefore recommending the authors revise and resubmit.

Below, you will find the bulk of my suggestions, and there are a few comments also added directly to the attached PDF.

**Abstract**

- Line 26 – you use the acronym US before introducing the full spelling.

**Introduction**

- Beyond your own previous study, how does your study build or compliment existing studies that survey author behaviour as it relates to APCs, specifically in the US? Is there anything of use in the following study, for example: Olejniczak, A. J., & Wilson, M. J. (2020). Who’s writing open access (OA) articles? Characteristics of OA authors at Ph.D.-granting institutions in the United States. *Quantitative Science Studies*, *1*(4), 1429–1450. <https://doi.org/10.1162/qss_a_00091>.
  - You could cite this study somewhere around lines 54-56, providing supporting evidence of attitudes and trends of the demographic makeup of who pays APCs in the US.
  - Or perhaps even around lines 77 or 78, further strengthening the argument not only of cognitive dissonance, but the socio-cultural impacts the influence author behaviour (e.g., gender).
- Lines 56 – 57: You argue that pricing APCs is based on prestige rather than production costs, which is an important argument. Before citing the literature that looks at prestige and hyperinflation, you may want to consider citing studies that examine this particular pricing strategy (prestige), then cite the effects of hyperinflation. For example,
  - Demeter, M., & Istratii, R. (2020). Scrutinising what Open Access Journals Mean for Global Inequalities. *Publishing Research Quarterly*, *36*(4), 505–522. <https://doi.org/10.1007/s12109-020-09771-9>
  - Schönfelder, N. (2020). Article processing charges: Mirroring the citation impact or legacy of the subscription-based model? *Quantitative Science Studies*, *1*(1), 6–27. <https://doi.org/10.1162/qss_a_00015>
- In your methods, you state that you group the surveys by three disciplines. This is a particularly important approach since disciplinary practices are so unique, and studies have shown APC pricing can differ across disciplines. You may want to consider citing this factor, you do not necessarily need to be exhaustive but pointing out to a relevant study or two can further strengthen your methodological choice.
- Line 62: starting with “Journal editors…” reads as though ALL journal editors share this view, but I would caution not to generalize that statement as editorship functions differently across journals.

**Materials and methods:**

- I think a bit of restructuring in the methods section, with clearer subheadings, could aid with readability. A good example of a study using surveys with nice subsections: <https://doi.org/10.1371/journal.pone.0301251> and <https://doi.org/10.1162/qss_a_00264> . For example, in line 171 of your paper: “While there were 321 completed surveys, 154 of those also included usable…”, is really about response rates, which you could expand upon. You can move this sentence to a new section (something like Survey Responses, or Response Rates), and you can expand upon the response rate to include details like the rate of completion for the two open-ended questions, or perhaps whether any respondents in the quantitative part of the survey dropped out and did not respond to the open-ended questions? Did you receive more responses for the gold than hybrid (or vice versa) surveys, or did respondents evenly complete both? What about responses by field – are respondents proportionally represented across the disciplines, or did you receive more responses from a certain field? Table 1 could be moved here as well, since the table does not really expand on the discussion, but rather visualizes a result.
- Lines 125 – 127: expand upon the funding profile of these institutions (and my apologies if this is convered elsewhere) – are researchers typically well-funded? In your intro, you argue about under-resourced institutions and certain geographic areas being disadvantaged by the APC trap; did you look at this variable in your study? If yes, this should be made clearer in the methods section.
- Can you expand a bit on your coding method? It was done inductively, correct? Lines 193-195 hint at this (through discussion).
- Line 199: you could add a bit of detail on *why* you chose not to analyze the additional theme *Presupposition of Cost.*
- Lines 199 – 202: “Respondents also conveyed broader sentiments, including frustration…” reads more like a result than a method, but follows your description of coding methods. Suggest moving this statement to your results section, and instead discuss here how you coded for the themes of frustration and appreciation.
- Lines 658 -682 (Section on Sentiment Analysis) –
  - Reviewer 2 suggests a citation, and I still do not see one added. I agree this is important to support your critiques of this method. You state in line 677 that these tools “often fail to grasp…”; the word *often* implies knowledge of this challenge elsewhere. I am curious if other studies have also experience similar challenges? This would be helpful information for future studies aiming to use this method.
  - Additionally, this section seems awkward here. Since you mention, in line 676, how the results highlight a limitation, is this better in the limitation section?

**Results section**

- I understand Reviewer 2 suggested the section previously titled “Code book” seems more like findings. I would still suggest you nevertheless provide a brief overview in your methods section on the 8 categories themselves, perhaps after you introduce your approach to manual coding.
- In your results section, you could provide a table that includes the 8 codes and the number of coded responses per category, to visualize how influential the codes are on author behaviour. Then this could be further unpacked in the discussion section – not surprising that your code “financial concerns” is the most frequent, and interesting that 30% of the results include aspects of the code Diversity of equity.
- Your section on “Codes” can be framed in a way that emphasizes the results – see comment on page 61, lines 272 in PDF.
- See comment in PDF, line 303 – follow same format for results (n=46; 28%) as the former sections.
- Line 420 – Presupposition of cost – is this a 9^th^ code? I’m confused, since you specified 8. Why is this not considered the 9^th^ if you coded responses according to this categorical topic? See my comment in PDF, around line 429-430.
- Line 471 – this argument is unclear to me – I am not grasping what you mean by “a certain instance of that model is to blame”. What do you mean by “instance”?

**Discussion**

- I am struggling with this section as much of the detail reads more like results than a discussion. I suggest revisiting and moving any findings up to your results section and/or cutting any duplication.
- The Discussion section should stick to exploring your findings (not restating them) - what factors are influencing the behaviour - why do you think so many respondents touched on diversity and equity themes? What is the impact of this for institutions and, more importantly, R1 institutions who are well-resourced? Etc…
- Line 488-492 is repeated information already detailed in your methods section, although you can move up the part about “usable” if not discussed earlier.
- Lines 500-502 could serve as the start of this section, since it reads more like a summary of findings.
- Line 514 – what are APC elements? This is a new concept being introduced. Are “elements” the “codes”? If yes, please clarify in the manuscript. If they differ from codes, please better define elements. Even more, as a reader, I am left struggling to understand why the results from this analysis are in the Discussion section, while the results from the codes are in the Results section?
- In my comments under the Results section, I suggest tabling the number of responses by code, so it is great that you chose to include a figure visualizing the elements (although again, how does this differ from codes?). However, this figure could move to your Results section, if you choose to expand upon response rates as suggested.
- Line 542 – 547 – this is the type of information I would expect to read in a discussion section. What about the other codes? Can you point to other literature that supports or extends possibilities for your findings?
- Line 549 - 555 **and** Tables 2 & 3 belongs more in Results section, can even be situated in a section on Survey Responses, providing valuable information on the demographics of respondents.
- Much of the information in Lines 582 until the end of discussion belong in the Results section. I suggest the authors review this section again, pull up any data to Results, and then summarize key points in your Discussion, and make claims about the results themselves, citing relevant studies where relevant that may support your claims, or where you are extending ideas from other studies. Section – “The messy knot of the APC Trap” – line 842 provides a nice example where you do discuss the findings.
- While I understand you used your 2022 dataset, Transformative Agreements / Read and Publish agreement have continued to increase in popularity. The fact that these often include full waivers for hybrid journals, over gold, make me wonder if they are influencing author behaviour, whereby we see authors selecting journals based on the 100% discount. How does this influence your results? Is it possible that a new type of “trap” includes authors preferring prestigious journals with full waivers owing to their institutions TAs? Do the institutions you study have TAs in place? Particular relevant since your initial dataset cleans the data by the corresponding author field.

**Limitations**

- Lines 887 – 888 – “and so its implications for smaller and less well-resources institutions…are limited”. What exactly do you mean by implications, here? Whether the results can provide any meaning for smaller institutions? I would argue that because you used purposive sampling, your results cannot be generalized beyond your dataset, so you may want to tweak this sentence a bit.

**Conclusion**

- Line 896 – I would suggest softening the language in line 896, specifically the use of the word “confirm”. Can the study confirm the concerns in the U.S.? You used purposive sampling, so your results can only be generalized to the respondents in your dataset. You state in your limitations that your study focused on authors are very research-intensive institutions, so its implications for smaller and less-resourced institutions are limited. You could instead allude that the results support other findings regarding inequities, and then cite a few studies.
- Line 901-902 – the limited scope is also that you used purposive sampling, and perhaps there are authors outside the dataset with other tendencies and behaviours.
