# Supplementary material for: “The system is obviously bonkers”: The APC Trap and the bind of scholarly publishing across four research intensive institutions in the U.S
Source: PLoS One. 2026 Jul 1;21(7):e0351430. doi: 10.1371/journal.pone.0351430 (PMC13322547; doi:10.1371/journal.pone.0351430)
Supplement: Peer review history

# Peer review: Decision Letter 0

**DOI:** 10.1371/journal.pone.0351430.r001

**Authors:** Alison Parker

21 Oct 2025

PONE-D-25-47145

"The system is obviously bonkers": The APC Trap and the bind of scholarly publishing across four R1 institutions

PLOS ONE

Dear Dr. Cantrell,

Thank you for submitting your manuscript to PLOS ONE. After careful consideration, we feel that it has merit but does not fully meet PLOS ONE’s publication criteria as it currently stands. Therefore, we invite you to submit a revised version of the manuscript that addresses the points raised during the review process.

Both reviewers recognise the importance of the manuscript but are concerned that it is too hard to understand by non-US readers, which was my concern when sending it for review.   They provide some constructive feedback on how to address this, as well as other points.

Please submit your revised manuscript by Dec 05 2025 11:59PM. If you will need more time than this to complete your revisions, please reply to this message or contact the journal office at plosone@plos.org. When you're ready to submit your revision, log on to https://www.editorialmanager.com/pone/ and select the 'Submissions Needing Revision' folder to locate your manuscript file.

Please include the following items when submitting your revised manuscript:

- A rebuttal letter that responds to each point raised by the academic editor and reviewer(s). You should upload this letter as a separate file labeled 'Response to Reviewers'.
- A marked-up copy of your manuscript that highlights changes made to the original version. You should upload this as a separate file labeled 'Revised Manuscript with Track Changes'.
- An unmarked version of your revised paper without tracked changes. You should upload this as a separate file labeled 'Manuscript'.

If you would like to make changes to your financial disclosure, please include your updated statement in your cover letter. Guidelines for resubmitting your figure files are available below the reviewer comments at the end of this letter.

If applicable, we recommend that you deposit your laboratory protocols in protocols.io to enhance the reproducibility of your results. Protocols.io assigns your protocol its own identifier (DOI) so that it can be cited independently in the future. For instructions see: https://journals.plos.org/plosone/s/submission-guidelines#loc-laboratory-protocols. Additionally, PLOS ONE offers an option for publishing peer-reviewed Lab Protocol articles, which describe protocols hosted on protocols.io. Read more information on sharing protocols at https://plos.org/protocols?utm_medium=editorial-email&utm_source=authorletters&utm_campaign=protocols.

We look forward to receiving your revised manuscript.

Kind regards,

Alison Parker

Academic Editor

PLOS ONE

Journal Requirements:

When submitting your revision, we need you to address these additional requirements.

1. Please ensure that your manuscript meets PLOS ONE's style requirements, including those for file naming. The PLOS ONE style templates can be found at

https://journals.plos.org/plosone/s/file?id=wjVg/PLOSOne_formatting_sample_main_body.pdf and

https://journals.plos.org/plosone/s/file?id=ba62/PLOSOne_formatting_sample_title_authors_affiliations.pdf

2. Please include your full ethics statement in the ‘Methods’ section of your manuscript file. In your statement, please include the full name of the IRB or ethics committee who approved or waived your study, as well as whether or not you obtained informed written or verbal consent. If consent was waived for your study, please include this information in your statement as well.

3. We note you have included a table to which you do not refer in the text of your manuscript. Please ensure that you refer to Table 3 and 4 in your text; if accepted, production will need this reference to link the reader to the Table.

4. If the reviewer comments include a recommendation to cite specific previously published works, please review and evaluate these publications to determine whether they are relevant and should be cited. There is no requirement to cite these works unless the editor has indicated otherwise.

[Note: HTML markup is below. Please do not edit.]

Reviewers' comments:

Reviewer's Responses to Questions

**Comments to the Author**

1. Is the manuscript technically sound, and do the data support the conclusions?

The manuscript must describe a technically sound piece of scientific research with data that supports the conclusions. Experiments must have been conducted rigorously, with appropriate controls, replication, and sample sizes. The conclusions must be drawn appropriately based on the data presented.

Reviewer #1: Yes

Reviewer #2: Partly

**********

2. Has the statistical analysis been performed appropriately and rigorously?

Reviewer #1: I Don't Know

Reviewer #2: N/A

**********

3. Have the authors made all data underlying the findings in their manuscript fully available?

The PLOS Data policy requires authors to make all data underlying the findings described in their manuscript fully available without restriction, with rare exception (please refer to the Data Availability Statement in the manuscript PDF file). The data should be provided as part of the manuscript or its supporting information, or deposited to a public repository. For example, in addition to summary statistics, the data points behind means, medians and variance measures should be available. If there are restrictions on publicly sharing data—e.g. participant privacy or use of data from a third party—those must be specified.

Reviewer #1: Yes

Reviewer #2: Yes

**********

4. Is the manuscript presented in an intelligible fashion and written in standard English?

PLOS ONE does not copyedit accepted manuscripts, so the language in submitted articles must be clear, correct, and unambiguous. Any typographical or grammatical errors should be corrected at revision, so please note any specific errors here.

Reviewer #1: Yes

Reviewer #2: Yes

**********

5. Review Comments to the Author

Please use the space provided to explain your answers to the questions above. You may also include additional comments for the author, including concerns about dual publication, research ethics, or publication ethics. (Please upload your review as an attachment if it exceeds 20,000 characters)

Reviewer #1: This manuscript uses qualitative methods to provide insights into sentiment to APC. I am not an expert in qualitative analyses, but I have provided comments below:

The introduction would benefit from a paragraph that introduces the topic to the more generalist reader – i.e. before getting into what this paper examines. In fact, this could be achieved by moving lines 39 to 50 to later in the introduction, and starting the manuscript with lines 51 onwards.

Lines 81-88 should be removed from the introduction. This text covers results and discussion and so does not belong here. However, potentially lines 39-50 could be moved here to replace it

Line 91: citation missing.

Table 2: The acronyms (HSS, NSE and HM) need to be explained in full

Table 3: The acronyms (e.g. UCB) need to be explained in full

Table 4: The acronym (OA) needs to be explained in full

Lines 717-723: A comment seems to have been left here. Remove.

Limitations: I feel like the limitations need to be expanded. This is a relatively small sample from a very limited number of institutions. How generalisable are the findings? More needs to be said about this to make the paper more well rounded.

Reviewer #2: • This is a highly topical paper that focuses on an area of publication in science and social science disciplines that is undergoing considerable change but with mixed views about the impacts and implications. This paper offers some evidence that provides some insight into the topic.

• The writing is clear and focused.

• The evidence base is substantial drawing on open text responses to an online questionnaire survey of a moderate size

Major issues

• Abstract – redraft the abstract so that it makes sense on its own. It currently uses abbreviations and assumes implicit information about the US research landscape.

• Although this paper does not coin the term ‘APC Trap’, it draws on and elaborates on the idea – one which could become very influential. I think there is more potential to explain how this idea is more important across global academia and having tested the approach in a select group of US universities, these ideas should be expanded to consider global academic publishing. The paper arrives at this in the end.

• The questionnaire survey strategy focuses on 4 US research-intensive institutions and so the population from which the participants are drawn are more likely to have access to funds to cover the cost of APCs. The implications of this evidence base could be more clearly articulated.

• The article makes the reasonable argument that if these relatively privileged academic respondents in an affluent society provide evidence of their concerns and criticism of this model, then there is something to be further analysed.

• I suggest that the authors’ late argument about the motivation for the publication of this dataset in a separate paper (lines 754-762) should be brought more to a much earlier part of the manuscript to strengthen the introduction.

Avoid using ‘R1’ until it is explained for an international audience. Certainly do not use this terminology in the title or the abstract. Plos ONE is an international journal, so this needs to be made explicitly clear at the outset and I recommend the authors reflect on the wider global implications of what they have found in the specific context of US R1 institutions and their authors not just for other US-based researchers, but for researchers more widely. The manuscript does refer to the wider context later, so this is a relatively simple edit on the early part of the manuscript to make clear the scope of the paper. It needs to elaborate on the global implications of a study that focuses on a particular category of researcher that seems to be in a privileged position.

p.2-3 - I recommend a general contextual statement about the OA publishing model trends and the importance of APCs to that, rather than a previous publication the authors have written. This elevates the findings of the paper to addressing issues wider than a continuation of a previous paper published elsewhere, which seems to be how the first paragraph positions the paper and undersells its significance.

The paper should stand on its own terms. I do understand that in some disciplines and publications this is less important where the methods are less contentious and more standardised. However, PLOS One encourages more transparency, and this paper is in a branch of the research on academic publication that does traditionally include transparent discussion and justification of methods. For guidance, I refer the authors to the PLOS One policy on statistical reporting in the Submission Guidelines in which this useful general principle is worth considering: “The Materials and Methods section should provide enough detail to allow suitably skilled investigators to fully replicate your study.” This does not have to be detailed, but basic explanation of the recruitment process, the demographic background of the respondents and the significance of the evidence base are all needed. Note that your methods section has only 2 citations, both of which are for your own previous paper which raises a question about how well embedded in existing research your methods are.

P.7 Sentiment analysis. - This whole section is weakened at the outset with the statement to the effect that the authors did not expect it to yield much. The rest of the section describes how this function in a piece of software lived up to those low expectations. Although there is some reporting of the outcome, the findings from this is an insufficient basis to merit the attention it is given. Although the authors explain the criticisms of autocoding, they do not provide any sources for this. I recommend this section is removed from the manuscript and a summarised account of the useful findings are incorporated as a minor element of the analysis of the evidence.

p.8 - This manuscript does not report the total number of cases (ie academic researchers) included in the dataset. The manuscript mentions 136 responses (p.8, line 170) and 321 responses of which 154 ‘had a usable response’ (p.18, line 428), but I was unclear if ‘responses’ meant researchers or answers to the two questions.

p.15 - The section entitled Code Book, seems to actually be the findings or results section. This presents interesting selection of the responses gathered for this research. However, the discussion section presents the most important contribution. This is the discussion of the codes, though the paper is not as clear as it could be how these codes are arrived at. The discussion mentions discussion amongst the research team and conclusions. The manuscript provides no contextual description of the four institutions, so it is unclear why outcomes were interesting.

I therefore recommend that the manuscript provides:

1. A brief description of each of the four institutions and how they are positions as research institutions in the US. A revision should take account of an international audience that does not have the implicit knowledge of the research landscape and that explains the wider significance.

2. An explanation of how the authors view the focus on these four institutions as providing useful evidence that enables wider interpretations to be drawn.

3. A stronger justification of the rationale for writing this paper that draws on wider arguments.

4. A re-drafted methods section that clarifies the methods used and explains and justifies how this limited and purposive sample provide a basis for the manuscript to make broader interpretations.

Minor revisions

p.5 You refer to categorisation of publications based on ‘reporting in Dimensions’ without explaining this. A citation and brief explanation is required to clarify this for an international audience.

P. 13 & multiple – you have used abbreviations such as NIH (p. 13, 33, 34) and NSF (p.13). These should be in-full when first used.

p. 36 The conclusion brings into the discussion Plan S, SCOAP3 and S20 none of which have been discussed in the main part of the paper – nor were any of the three direct quotes from participants. I suggest these elements of the conclusion be moved to the discussion and the conclusion then rests only on the contents of the paper.

**********

6. PLOS authors have the option to publish the peer review history of their article (what does this mean?). If published, this will include your full peer review and any attached files.

If you choose “no”, your identity will remain anonymous but your review may still be made public.

**Do you want your identity to be public for this peer review?**  For information about this choice, including consent withdrawal, please see our Privacy Policy.

Reviewer #1: No

Reviewer #2: **Yes:**  Kenneth Lynch

**********

[NOTE: If reviewer comments were submitted as an attachment file, they will be attached to this email and accessible via the submission site. Please log into your account, locate the manuscript record, and check for the action link "View Attachments". If this link does not appear, there are no attachment files.]

To ensure your figures meet our technical requirements, please review our figure guidelines: https://journals.plos.org/plosone/s/figures

You may also use PLOS’s free figure tool, NAAS, to help you prepare publication quality figures: https://journals.plos.org/plosone/s/figures#loc-tools-for-figure-preparation.

NAAS will assess whether your figures meet our technical requirements by comparing each figure against our figure specifications.

Attachment Submitted filename: Review Report of PONE-D-25-47145.docx

# Author comment: Author response to Decision Letter 1

**DOI:** 10.1371/journal.pone.0351430.r002

2 Dec 2025

A letter detailing the revisions required and our response to them is included in the attached documents for this submission.

Attachment Submitted filename: PLOS One Revisions Letter.docx

# Peer review: Decision Letter 1

**DOI:** 10.1371/journal.pone.0351430.r003

**Authors:** Alison Parker

18 Mar 2026

PONE-D-25-47145R1

"The system is obviously bonkers": The APC Trap and the bind of scholarly publishing across four research intensive institutions in the U.S.

PLOS One

Dear Dr. Cantrell,

Thank you for submitting your manuscript to PLOS ONE. After careful consideration, we feel that it has merit but does not fully meet PLOS ONE’s publication criteria as it currently stands. Therefore, we invite you to submit a revised version of the manuscript that addresses the points raised during the review process.

- **After some delay we haves the third reviewer's comments. Although they are quite extensive they are only minor in nature I think.**

-->Please submit your revised manuscript by May 02 2026 11:59PM. If you will need more time than this to complete your revisions, please reply to this message or contact the journal office at plosone@plos.org. When you're ready to submit your revision, log on to https://www.editorialmanager.com/pone/ and select the 'Submissions Needing Revision' folder to locate your manuscript file.

Please include the following items when submitting your revised manuscript:

- A letter that responds to each point raised by the academic editor and reviewer(s). You should upload this letter as a separate file labeled 'Response to Reviewers'.
- A marked-up copy of your manuscript that highlights changes made to the original version. You should upload this as a separate file labeled 'Revised Manuscript with Track Changes'.
- An unmarked version of your revised paper without tracked changes. You should upload this as a separate file labeled 'Manuscript'.

-->If you would like to make changes to your financial disclosure, please include your updated statement in your cover letter. Guidelines for resubmitting your figure files are available below the reviewer comments at the end of this letter.

If applicable, we recommend that you deposit your laboratory protocols in protocols.io to enhance the reproducibility of your results. Protocols.io assigns your protocol its own identifier (DOI) so that it can be cited independently in the future. For instructions see: https://journals.plos.org/plosone/s/submission-guidelines#loc-laboratory-protocols. Additionally, PLOS ONE offers an option for publishing peer-reviewed Lab Protocol articles, which describe protocols hosted on protocols.io. Read more information on sharing protocols at https://plos.org/protocols?utm_medium=editorial-email&utm_source=authorletters&utm_campaign=protocols.

We look forward to receiving your revised manuscript.

Kind regards,

Alison Parker

Academic Editor

PLOS One

**Journal Requirements:**

1. If the reviewer comments include a recommendation to cite specific previously published works, please review and evaluate these publications to determine whether they are relevant and should be cited. There is no requirement to cite these works unless the editor has indicated otherwise.

2. Please review your reference list to ensure that it is complete and correct. If you have cited papers that have been retracted, please include the rationale for doing so in the manuscript text, or remove these references and replace them with relevant current references. Any changes to the reference list should be mentioned in the rebuttal letter that accompanies your revised manuscript. If you need to cite a retracted article, indicate the article’s retracted status in the References list and also include a citation and full reference for the retraction notice.

[Note: HTML markup is below. Please do not edit.]

Reviewers' comments:

Reviewer's Responses to Questions

**Comments to the Author**

1. If the authors have adequately addressed your comments raised in a previous round of review and you feel that this manuscript is now acceptable for publication, you may indicate that here to bypass the “Comments to the Author” section, enter your conflict of interest statement in the “Confidential to Editor” section, and submit your "Accept" recommendation.

Reviewer #1: All comments have been addressed

Reviewer #3: (No Response)

**********

2. Is the manuscript technically sound, and do the data support the conclusions?

The manuscript must describe a technically sound piece of scientific research with data that supports the conclusions. Experiments must have been conducted rigorously, with appropriate controls, replication, and sample sizes. The conclusions must be drawn appropriately based on the data presented.

Reviewer #1: Yes

Reviewer #3: Yes

**********

3. Has the statistical analysis been performed appropriately and rigorously?

Reviewer #1: Yes

Reviewer #3: Yes

**********

4. Have the authors made all data underlying the findings in their manuscript fully available?

The PLOS Data policy requires authors to make all data underlying the findings described in their manuscript fully available without restriction, with rare exception (please refer to the Data Availability Statement in the manuscript PDF file). The data should be provided as part of the manuscript or its supporting information, or deposited to a public repository. For example, in addition to summary statistics, the data points behind means, medians and variance measures should be available. If there are restrictions on publicly sharing data—e.g. participant privacy or use of data from a third party—those must be specified.

Reviewer #1: Yes

Reviewer #3: Yes

**********

5. Is the manuscript presented in an intelligible fashion and written in standard English?

PLOS ONE does not copyedit accepted manuscripts, so the language in submitted articles must be clear, correct, and unambiguous. Any typographical or grammatical errors should be corrected at revision, so please note any specific errors here.

Reviewer #1: Yes

Reviewer #3: No

**********

6. Review Comments to the Author

Please use the space provided to explain your answers to the questions above. You may also include additional comments for the author, including concerns about dual publication, research ethics, or publication ethics. (Please upload your review as an attachment if it exceeds 20,000 characters)

**Reviewer #1:**  All my comments have been addressed. Thank you.

I am happy for this manuscript to be accepted and do not see any further modifications necessary

**Reviewer #3:**  Overall, this study provides relevant and timely findings that advance our understanding of the attitudes of authors in regard to APCs. However, I do find the paper could be better structured to ease the reader’s ability to understand the methods and findings, restructuring the Results & Discussions sections, and a bit more finessing of language for accuracy. A bit more work is needed to streamline and make the writing more concise, and another read through to ensure acronyms are consistent (e.g., open access (OA)) throughout.

I am therefore recommending the authors revise and resubmit.

I thank the authors for this work, as it does prove valuable for institutions and library investment planning.

**********

7. PLOS authors have the option to publish the peer review history of their article (what does this mean?). If published, this will include your full peer review and any attached files.

If you choose “no”, your identity will remain anonymous but your review may still be made public.

**Do you want your identity to be public for this peer review?**  For information about this choice, including consent withdrawal, please see our Privacy Policy.

Reviewer #1: No

Reviewer #3: No

**********

[NOTE: If reviewer comments were submitted as an attachment file, they will be attached to this email and accessible via the submission site. Please log into your account, locate the manuscript record, and check for the action link "View Attachments". If this link does not appear, there are no attachment files.]

To ensure your figures meet our technical requirements, please review our figure guidelines: https://journals.plos.org/plosone/s/figures

You may also use PLOS’s free figure tool, NAAS, to help you prepare publication quality figures: https://journals.plos.org/plosone/s/figures#loc-tools-for-figure-preparation.

NAAS will assess whether your figures meet our technical requirements by comparing each figure against our figure specifications.

Attachment Submitted filename: PONE-D-25-47145_R1_w. a few reviewer comments.pdf

Attachment Submitted filename: Review_Cantrell et al_PLOS.docx

# Author comment: Author response to Decision Letter 2

**DOI:** 10.1371/journal.pone.0351430.r004

4 May 2026

Thank you for the additional feedback and suggested revisions for our paper “‘The System is Obviously Bonkers’: The APC Trap and the Bind of Scholarly Publishing across Four Research Intensive Institutions in the U.S." for consideration in PLOS One.

We conducted significant revisions for this second round of review, including a complete reorganization and partial re-write of the Results and Discussion sections; moving the sub-section on sentiment analysis to the Limitations section; and incorporating several new citations to back up claims made in the paper. The letter responding to reviewers provides an itemized list of the changes made to address reviewer concerns.

Attachment Submitted filename: Round2_PLOSOne_ResponsetoReviewers.pdf

# Peer review: Decision Letter 2

**DOI:** 10.1371/journal.pone.0351430.r005

**Authors:** Alison Parker

28 May 2026

"The system is obviously bonkers": The APC Trap and the bind of scholarly publishing across four research intensive institutions in the U.S.

PONE-D-25-47145R2

Dear Dr. Cantrell,

We’re pleased to inform you that your manuscript has been judged scientifically suitable for publication and will be formally accepted for publication once it meets all outstanding technical requirements.

Within one week, you’ll receive an e-mail detailing the required amendments. When these have been addressed, you’ll receive a formal acceptance letter and your manuscript will be scheduled for publication.

An invoice will be generated when your article is formally accepted. Please note, if your institution has a publishing partnership with PLOS and your article meets the relevant criteria, all or part of your publication costs will be covered. Please make sure your user information is up-to-date by logging into Editorial Manager at Editorial Manager® and clicking the ‘Update My Information' link at the top of the page. For questions related to billing, please contact billing support.

If your institution or institutions have a press office, please notify them about your upcoming paper to help maximize its impact. If they’ll be preparing press materials, please inform our press team as soon as possible -- no later than 48 hours after receiving the formal acceptance. Your manuscript will remain under strict press embargo until 2 pm Eastern Time on the date of publication. For more information, please contact onepress@plos.org.

Kind regards,

Alison Parker

Academic Editor

PLOS One

Additional Editor Comments (optional):

Reviewers' comments:

Reviewer's Responses to Questions

**Comments to the Author**

1. If the authors have adequately addressed your comments raised in a previous round of review and you feel that this manuscript is now acceptable for publication, you may indicate that here to bypass the “Comments to the Author” section, enter your conflict of interest statement in the “Confidential to Editor” section, and submit your "Accept" recommendation.

Reviewer #3: All comments have been addressed

**********

2. Is the manuscript technically sound, and do the data support the conclusions?

The manuscript must describe a technically sound piece of scientific research with data that supports the conclusions. Experiments must have been conducted rigorously, with appropriate controls, replication, and sample sizes. The conclusions must be drawn appropriately based on the data presented.

Reviewer #3: Yes

**********

3. Has the statistical analysis been performed appropriately and rigorously?

Reviewer #3: Yes

**********

4. Have the authors made all data underlying the findings in their manuscript fully available?

The PLOS Data policy requires authors to make all data underlying the findings described in their manuscript fully available without restriction, with rare exception (please refer to the Data Availability Statement in the manuscript PDF file). The data should be provided as part of the manuscript or its supporting information, or deposited to a public repository. For example, in addition to summary statistics, the data points behind means, medians and variance measures should be available. If there are restrictions on publicly sharing data—e.g. participant privacy or use of data from a third party—those must be specified.

Reviewer #3: Yes

**********

5. Is the manuscript presented in an intelligible fashion and written in standard English?

PLOS ONE does not copyedit accepted manuscripts, so the language in submitted articles must be clear, correct, and unambiguous. Any typographical or grammatical errors should be corrected at revision, so please note any specific errors here.

Reviewer #3: Yes

**********

6. Review Comments to the Author

Please use the space provided to explain your answers to the questions above. You may also include additional comments for the author, including concerns about dual publication, research ethics, or publication ethics. (Please upload your review as an attachment if it exceeds 20,000 characters)

Reviewer #3: Thank you for addressing both mine and the other reviewer comments and suggestions. The authors have made significant improvements that make the paper clearer and more readable. I find this revised version acceptable for publication and thank the authors for their valuable work and contributions to this ever-evolving area of scholarly publishing.

**********

7. PLOS authors have the option to publish the peer review history of their article (what does this mean?). If published, this will include your full peer review and any attached files.

If you choose “no”, your identity will remain anonymous but your review may still be made public.

**Do you want your identity to be public for this peer review?**  For information about this choice, including consent withdrawal, please see our Privacy Policy.

Reviewer #3: No

**********

# Editor report: Acceptance letter

**DOI:** 10.1371/journal.pone.0351430.r006

**Authors:** Alison Parker

PONE-D-25-47145R2

PLOS One

Dear Dr. Cantrell,

I'm pleased to inform you that your manuscript has been deemed suitable for publication in PLOS One. Congratulations! Your manuscript is now being handed over to our production team.

At this stage, our production department will prepare your paper for publication. This includes ensuring the following:

* All references, tables, and figures are properly cited

* All relevant supporting information is included in the manuscript submission,

* There are no issues that prevent the paper from being properly typeset

You will receive further instructions from the production team, including instructions on how to review your proof when it is ready. Please keep in mind that we are working through a large volume of accepted articles, so please give us a few days to review your paper and let you know the next and final steps.

Lastly, if your institution or institutions have a press office, please let them know about your upcoming paper now to help maximize its impact. If they'll be preparing press materials, please inform our press team within the next 48 hours. Your manuscript will remain under strict press embargo until 2 pm Eastern Time on the date of publication. For more information, please contact onepress@plos.org.

You will receive an invoice from PLOS for your publication fee after your manuscript has reached the completed accept phase. If you receive an email requesting payment before acceptance or for any other service, this may be a phishing scheme. Learn how to identify phishing emails and protect your accounts at https://explore.plos.org/phishing.

If we can help with anything else, please email us at customercare@plos.org.

Thank you for submitting your work to PLOS ONE and supporting open access.

Kind regards,

PLOS ONE Editorial Office Staff

on behalf of

Dr. Alison Parker

Academic Editor

PLOS One
